# Constraining the population size estimates of the pre-Columbian Casarabe Culture of Amazonian Bolivia

**DOI:** 10.1371/journal.pone.0325104

**Published:** 2025-05-30

**Authors:** Joseph Hirst, Joy S. Singarayer, Umberto Lombardo, Francis Mayle

**Affiliations:** 1 Department of Geography and Environmental Science, School of Archaeology, Geography and Environmental Science, University of Reading, Whiteknights, Reading, United Kingdom; 2 Department of Meteorology, School of Mathematical, Physical, and Computational Sciences, Earley Gate, University of Reading, Reading, United Kingdom; 3 Department of Prehistory, Institut de Ciència i Tecnologia Ambientals (ICTA-UAB), Universitat Autònoma de Barcelona, Edifici ICTA-UAB, Cerdanyola de Vallès, Barcelona, Spain; University of Michigan, UNITED STATES OF AMERICA

## Abstract

The capacity of Amazonian environments to support large indigenous societies prior to European Contact has long been a contentious area of debate, particularly in regions where pre-Columbian cultures are known to have constructed large, spatially complex earthworks. Here, we provide the first range of supported population estimates for the Casarabe Culture of the Bolivian Llanos de Moxos – one of the most complex pre-Columbian societies yet documented in Amazonia. Between 400 and 1400 CE, the Casarabe Culture inhabited this forest-savanna mosaic landscape, where they constructed hundreds of monumental habitation mounds, integrated by a dense network of causeways and canals, suggesting the former presence of a large, sedentary society. To estimate the population size of this culture, we employed a multifaceted modelling approach – including architectural energetics, maximum carrying capacity, and agent-based modelling – which considers: (i) the number of people needed to build these earthworks; (ii) how many people the local environment could support; and (iii) how their population grew and spread over time. Our results indicate that the Casarabe Culture likely grew to a maximum population of between 10,000 and 100,000 people within a 5020 km^2^ quadrant of their former territory, representing a density of between 2 and 20 people km^-2^. These values are considerably larger than both the modern rural population density and the indigenous carrying capacity estimates made for Amazonia more widely, and they support previous interpretations that this culture practiced a form of low-density urbanism.

## Introduction

Scholars have long debated whether Amazonian environments were able to support large, complex societies prior to European Contact [1–4]. Traditionally, these environments were thought to restrict human occupation to small, semi-sedentary communities of <100 people [1,5,6], but multiple lines of evidence now challenge this perspective [7,8]. In particular, the documentation of numerous complex archaeological sites across the basin now suggests that much larger populations existed within certain localities [9–13]. One such locality is the *Llanos de Moxos* (LM), a vast (120,000 km^2^) seasonally flooded forest-savanna mosaic landscape in northern lowland Bolivia. Today, this region is sparsely populated at an average rural density of <1 person per km^-2^, with most of its 520,000 inhabitants concentrated in the towns of Trinidad and Riberalta [14]. However, the LM also contains a variety of earthworks which provide evidence to suggest that its pre-Columbian population was much larger [15,16]. Perhaps most well-known are the earthworks found in the LM’s southeastern sector, where the now-extinct Casarabe Culture constructed at least 189 earthen habitation mounds, interconnected by a dense network of causeways, canals, and lakes [17]. Reaching up to 20 ha in surface area and 20 m in height, the size and spatial complexity of these mound structures suggest that the Casarabe Culture engaged in a form of low-density agrarian urbanism [18]. Exploiting fertile sediments deposited in the southeastern LM during the late Holocene [19], palaeobotanical evidence shows that this culture utilised their causeway-canal system as a drainage and irrigation network to practice intensive maize monoculture in the open savannas [17,19–21]. Recent skeletal carbon isotope analyses indicate that this maize formed a central component of their diet [22].

The presence of these earthworks strongly suggests that the southeastern LM once supported a large, sedentary population. Many of the mounds are directly integrated within the causeway-canal network [18], indicating that they were contemporaneously occupied. Radiocarbon dates obtained from several of the mounds show that this culture continuously inhabited the southeastern LM for a whole millennium between 400 and 1400 CE [23–25]. This evidence implies that, despite reaching a sufficient population density to simultaneously occupy their network of earthworks, the Casarabe Culture was still able to support itself here in the long-term. Such a feat appears incompatible with the <1 person km^-2^ carrying capacity estimates proposed for Amazonia more widely [26–28]. If the population size of the Casarabe Culture was indeed large, it raises numerous questions about how they utilised the southeastern LM to support themselves, as opposed to the cattle ranching and mechanised rice agriculture strategies employed today [29–31].

**Fig 1:**
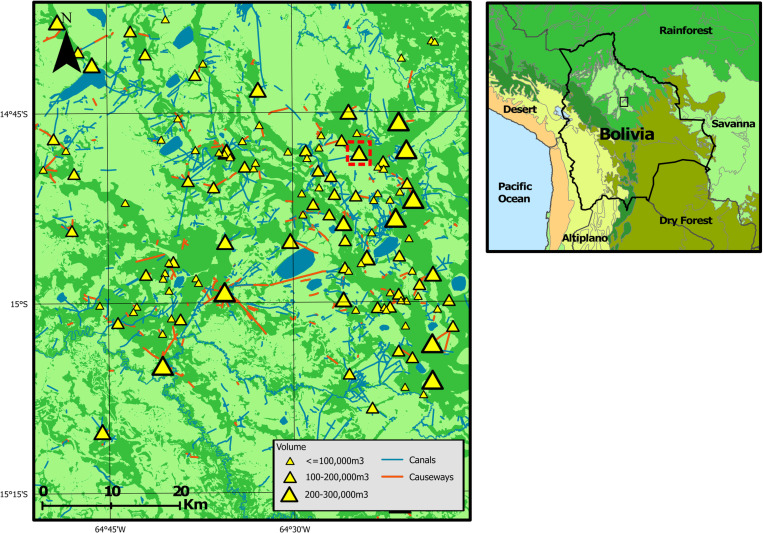
Map displaying the earthworks of the southeastern LM, set against a land cover classification demarcating areas of tropical forest (green), savanna (light green), and water (blue). Earthworks are a modified version of the dataset produced in Lombardo and Prümers [17]. The red box highlights the mound site for which population estimates have been proposed [32]. Inset map displays the location of the main map in relation to central South America, set against a map of terrestrial ecoregions [33], colour-coded into areas dominated by tropical forest (green); savanna (light green); yungas (dark green); tropical dry forest (gold); altiplano (yellow); and desert (orange). Modified from [34] under a CC BY license. Original copyright 2025.

To date, no systematic, rigorous attempt has been made to estimate the population size of the Casarabe Culture. The only published figure proposes that between 500 and 1000 people inhabited the medium-sized mound located close to Ibiato village (red box, Fig 1) [32]. However, while these figures align with the statements made in historical European texts, which claim that the LM once contained villages with as many as 2000 inhabitants [35,36], they are entirely speculative and contain no supporting evidence. Two unpublished estimates have also been made by co-author Lombardo [37], but these span multiple orders of magnitude, ranging between 3000 and 250,000 people for a 4500 km^2^ area of the southeastern LM where the Casarabe Culture’s earthworks have been mapped in detail [17]. Beyond these, the only available estimates are applicable to either the wider LM or Amazonia more generally (Table 1), and these further extend the lower end of Lombardo’s range down to just 750 people (0.15 people km^-2^) [40]. Such large uncertainties highlight the difficulties in constraining population estimates with limited archaeological evidence.

**Table 1:** Previous population estimates applicable to the Casarabe Culture sourced from the wider literature. The ‘Projected Population’ variable applies population density estimates to our study area, highlighted in Fig 1 and discussed below.

Author	Region	Method	Population Density Estimate	Projected Population for our study area
Meggers [28]	Amazonia	Carrying Capacity	0.3 km^-2^	1506
Meggers [27]	Amazonia	Carrying Capacity	0.2-1.0 km^-2^	1004 - 5020
Denevan [38]	Llanos de Moxos	Habitat Density	2.0 km^-2^	10,040
Métraux [39]	Llanos de Moxos	Jesuit Count	30−100 settlement^-1^	3570 - 11,900
Steward [40]	Llanos de Moxos	Jesuit Count	0.15 km^-2^	753
Steward and Faron [41]	Llanos de Moxos	Jesuit Count	0.23 km^-2^	1155
Erickson [32]	Southeastern LM	N/A	500−1000 settlement^-1^	59,500 − 119,000
Lombardo [37]	Region studied in Lombardo and Prümers [17]	Architectural Energetics	0.61 km^-2^	3062
Lombardo [37]	Region studied in Lombardo and Prümers [17]	Carrying Capacity	50 km^-2^	251,100

Despite the significant challenges involved in calculating palaeopopulation estimates, two unique characteristics can help constrain these calculations for the Casarabe Culture. Firstly, the atypical dominance (in comparison with most of Amazonia) of forest-savanna mosaic vegetation in this landscape greatly restricts the availability of various forest resources, many of which are necessary for survival (e.g., fuelwood). Secondly, unlike many other contemporaneous pre-Columbian (pre-1492 CE) indigenous groups, the Casarabe Culture intentionally chose to construct and settle atop earthen mounds, elevated above the seasonal flood waters. This distinctive practice enables us to pinpoint where on the landscape the members of this culture were once concentrated. Both of these characteristics can be combined to further parameterise and inform any attempt to model the population size of this culture.

In this article, we provide the first range of systematically derived population estimates for the Casarabe Culture. We obtain these estimates by employing a multifaceted modelling approach which combines three distinct methodologies: architectural energetics, maximum carrying capacity, and agent-based modelling. Our objectives are: (i) to generate initial upper and lower population boundaries for the Casarabe Culture using the architectural energetics and carrying capacity methods; (ii) to further constrain this range by comparing these boundary estimates with the results generated by our agent-based model, which we developed to explore the growth of the Casarabe Culture over time; and (iii) to identify which of the assumptions made during our model experiments most accurately recreate the real landscape. This multifaceted approach reduces the influence of limitations and assumptions associated with the above methods [4], allowing us to generate population estimates for the Casarabe Culture across a range of scenarios. Some of these limitations are introduced in the methods section, and we explore the limitations of our own approach in greater detail in the ‘Numbers from Nowhere’ subsection of the discussion.

## Materials and methods

### Study area

The LM is a seasonally-flooded forest-savanna mosaic landscape situated on the southwestern periphery of Amazonia. Up to 80,000 km^2^ of this landscape becomes inundated on a seasonal basis, predominantly from pluvial flooding [42,43]. This inhibits tree growth in low-lying areas, restricting the establishment of tropical forests to a dendritic network of palaeoriver levees extending across the landscape [44,45]. Taking advantage of this higher ground, the Casarabe Culture built a network of settlement mounds atop these levees. The mounds themselves consist of an elevated platform, normally circular or elliptical in shape, and usually topped by a pyramidal structure [17,18]. Archaeological excavations show these structures were built in stages from interwoven layers of clay and domestic refuse, and that their primary use was for habitation [46,47]. Large quantities of ceramics were incorporated into the refuse, which have subsequently been used to reconstruct a five-stage chronological sequence of occupation spanning from 400 to 1400 CE [23–25]. The mounds are directly integrated into the wider causeway-canal system, sometimes being encircled by canals and/or polygonal enclosures [17]. In general, the causeways and canals are constructed adjacent to one another [36], with the former being comprised of canal fill, and the latter typically reaching 1 m in depth [19]. Aside from interlinking the settlement mounds, the canals are connected to nearby rivers and lakes, and have been proposed to serve multiple functions that include drainage, irrigation, and providing water to the mound settlements [17,20,48].

The population estimates generated within this study explicitly apply to the 5020 km^2^ quadrant of the southeastern LM displayed in Fig 1. This quadrant fully encompasses the earthworks which have been mapped in the greatest detail [17], though note that the area previously occupied by the Casarabe Culture extends beyond these boundaries [18,36].

### Palaeopopulation models

The first stage of our multifaceted modelling approach is to generate minimum and maximum population boundary estimates for the Casarabe Culture. To calculate the minimum population estimate, we employ an architectural energetics approach. The labour resources required to construct the mounds, causeways, and canals still visible in the southeastern LM today [17] must have been directly extracted from members of the Casarabe Culture. Architectural energetics aims to quantify the effort involved in such a task [49,50], providing insight into both the size of the minimum requisite workforce and the population needed to sustain it. An important limitation of this method is its reliance on the estimated labour costs of construction activities, many of which vary between sites [49]. Nevertheless, while a complete range of site-specific cost estimates for the Casarabe Culture is unavailable, filling these gaps with suitable costs from the wider literature should suffice to produce an order-of-magnitude estimate of the regional population. Architectural energetics estimates calculated for the Casarabe Culture should be interpreted as a minimum population estimate because: (i) some earthworks are no longer visible, and (ii) building these structures represents additional labour on top of normal subsistence activities (e.g., cultivation).

We obtain our maximum population estimates using a maximum carrying capacity approach [51]; whereby we quantify the largest number of people that our study area could support through maize cultivation and fuelwood extraction. There are a number of limitations associated with such an approach, including the possibility of capacity growing over time through cultural and technological innovation [52], as well as the importance of choosing appropriate limiting factors [53]. We selected maize and fuelwood because maize is ubiquitously present across Casarabe Culture sites [20,47,54] and constitutes a dominant component of their diet [22], while fuelwood is relatively scarce on the landscape because it can only be extracted from the limited areas of forested land, spatially constrained by *terra firme* (non-flooded) micro-topography on palaeoriver levées. 

We further constrain these initial boundaries based on the outputs of our agent-based model, *MoundSim Population.* Agent-based models are characterised by their focus on the emergent behaviour of individual ‘agents’, resulting from their interactions with one another and their surrounding environment. In our model, these agents represent household units—comprising adults and children—that collectively form a representation of the Casarabe Culture within a gridded virtual landscape. Programmed to subsist (through maize-based agriculture), reproduce, and redistribute themselves across this virtual landscape, our household agents enable *MoundSim Population* to function like a ‘virtual laboratory’. The outputs of our model allow us to explore how the culture could have developed under a range of assumptions [55,56]. In doing so, we also determine which parameter assumptions most closely recreate the number and spatial distribution of settlement mounds on the real landscape.

### Architectural energetics

To produce energetics-based population estimates for the Casarabe Culture, we first revised previous estimates for the amount of earth required to build their earthworks [17] to account for: (i) new volumetric data obtained from recent LiDAR scans [18]; (ii) additional earthworks identified subsequent to previous mapping [17]; (iii) the infilling of canals and borrow pits; and (iv) earth lost from the settlement mounds due to erosion. Following previous work [17], we estimate the volume of each mound by using a surface area: volume ratio based on the sites with either existing LiDAR data or a high-resolution digital elevation model (DEM). For the causeway-canal network, we assumed that 5 m^3^ of earth was excavated to produce one linear metre of each earthwork [17]. The labour effort required to move this earth was assumed to be expended linearly over time, with the Casarabe Culture moving 1/1000^th^ of the total volume annually. We assume that the earth eroded from these structures represented a consistent proportion of the total earth comprising them.

To estimate the rate at which earth could be excavated, we utilised an experiment conducted in the LM [57] in which workers were asked to create pond features. The workers were not asked to transport the earth and were paid daily (in 8-hour workdays), according to the quantity of material excavated. These experiments showed that workers were able to excavate between 1.7 and 4.7 m^3^ of earth daily, rates that are well within the range for other experiments conducted on dense soils [49]. However, workers were allowed to complete this task using metal tools. Even stone tools were a scarce commodity for the Casarabe Culture [47], as the total absence of rock outcrops in the southeastern LM meant that any such tool needed to be obtained via long-distance trade. We therefore apply the 1:2.7 ratio of Erasmus [58] to account for the decrease in efficiency from metal tools to digging sticks, reducing the expected excavation efficiency to between 0.3 and 1.4 m^3^ person^-1^ day^-1^. To be comprehensive, we present energetics estimates across the entire range of these assumed values.

Any earth excavated for the causeway-canal network is assumed to have been transported only a short distance (5 m), as these features were constructed adjacent to one another [36]. For the mounds, we assume the earth was obtained from pits/canals adjacent to the feature, before being transported upslope and deposited on the main mound platform. These pits were then intentionally infilled as the mound grew. To calculate the earth transportation rate, we employed a modified version of the formula developed by the United Nations [59,60], which expresses transport rate (E_t_, m^3^ day^-1^) in relation to the transported load (Q, kg); distance travelled (L, km); time spent working (H, hours day^-1^); and speed of travel (V, km hr^-1^). Prior to calculation, we convert Q into an estimate for the volume of earth transported, dividing it by the average density of clay-dominated soil (1500 kg m^-3^; ρ). L and V are divided into portions to account for the effort involved in constructing the mounds (L_m_, V_m_) and causeway-canal network (L_c_, V_c_), as well as to account for the effort involved in transporting earth over flat ground (f), upslope (s), and on the return trip without transporting earth (‘):


$$\begin{array}{c} E_{t}=\left[\frac{Q}{\rho }*\frac{1}{\left(\frac{L_{mf}}{V_{mf}}+\frac{{L_{m}}^{\prime }}{{V_{m}}^{\prime }}+\frac{L_{ms}}{V_{ms}}\right)}*H\right]+\left[\frac{Q}{\rho }*\frac{1}{\left(\frac{L_{c}}{V_{cf}}+\frac{L_{c}}{{V_{c}}^{\prime }}\right)}*H\right] \end{array}$$ (1)

We assumed that workers travelled at an unloaded walking speed of 5 km hour^-1^, and that each expended a constant amount of energy irrespective of external conditions. As such, changes to these conditions (e.g., load carried, slope) are expected to influence travel speed. To quantify these changes, we employed the formula of Pandolf and colleagues [61,62], which calculates the metabolic rate of humans relative to body mass (kg, M), Q and V as described above, the slope gradient (%, G), and a terrain modifier representing the difficulty of traversing an environment (μ) [63].


$$\begin{array}{c} MetabolicRate=1.5M+2(M+Q){\left(\frac{Q}{M}\right)}^{2}+\mu (M+Q)(1.5V^{2}+0.35VG) \end{array}$$ (2)

To calculate the size of the required workforce, we converted the volume of earth needed to build the mounds (T_m_) and the causeway-canal network (T_c_, m^3^) into estimates of earth moved per year (T_md_, T_cd_). This was done by dividing total estimates by the assumed number of days worked per year (D; 18) and the duration of mound occupation (Y, years):


$$T_{md}=\frac{T_{m}}{\left(D*Y\right)}$$ (3)

$$T_{cd}=\frac{T_{c}}{\left(D*Y\right)}$$ (4)

Finally, workforce size (P_W_) is derived by dividing the total amount of earth needed to be moved daily by the excavation and transportation rates for both the mounds (E_mx_, E_mt_) and the causeway-canal network (E_cx_, E_ct_):


$$P_{w}=\left[\left(\frac{T_{md}}{E_{mx}}\right)+\left(\frac{T_{md}}{E_{mt}}\right)\right]+\left[\left(\frac{T_{cd}}{E_{cx}}\right)+\left(\frac{T_{cd}}{E_{ct}}\right)\right]$$ (5)

This result is converted to a total population estimate by assuming it constitutes between 20 and 50% of the population, following estimates made at other sites [64]. At lower ratios, this proportion assumes certain people were unable to participate (e.g., for being elderly or too young). Meanwhile, higher ratios assume that only adult males took part in earthwork construction. A full set of calculations for our estimates can be found in the supplementary information (S1 File).

### Carrying capacity

Our carrying capacity estimates are based on the *per capita* quantity of land required by the Casarabe Culture for maize cultivation and fuelwood extraction. While this culture is known to have utilised the open savannas, it remains unclear whether they also practiced cultivation in the forests similar to modern indigenous groups [13,17,20]. For this reason, we calculated the carrying capacity for maize and fuelwood under three different scenarios: (i) cultivation solely occurred in the open savannas; (ii) maize was grown in both the savannas and the forests; and for comparison, (iii) cultivation was restricted solely to forested areas. Although the Casarabe Culture may have employed these forests for other types of resource production (e.g., agroforestry), for simplicity, our carrying capacity model assumes any forested land used for cultivation was clear-cut and maize monoculture planted. Two sets of population estimates were produced for each of the above scenarios, one assuming all the resources on the landscape could be exploited, the other restricting accessible resources to those within daily walking distance of the settlement mounds, defined as 7 km based on the walking distances recorded for modern Amazonian indigenous groups [65]. Our estimates below assume that the Casarabe Culture cultivated maize as part of a swidden-fallow system. Please note that this assumption is made for the sake of simplicity, as little information exists about the Casarabe Culture’s farming practices, especially within the forests.

First, we calculate the *per capita* land requirement for maize cultivation (L_PM_) following the formula developed by Fearnside [53,66]. To use this formula, we make assumptions regarding the quantity of maize consumed by an individual per harvest cycle (Cons_P_), the yield of maize per hectare (L_R_), and the ‘Cultivation Factor’ (C), a value that varies depending upon the length of both the fallow period (Fal_yr_) and the crop cycle (Crop_yr_):


$$\mathrm{\backslash [}L_{PM}={Cons}_{P}*C/L_{R}\mathrm{\backslash ]}$$

$$C=({Fal}_{yr}/{Crop}_{yr}+1$$ (6)

We also calculate a *per capita* land requirement for fuelwood extraction (L_PF_), using a similar formula that omits C by assuming a one-year ‘cultivation period’ and no fallow time. To convert this into carrying capacity (k), we estimated the quantity of forest and savanna available to the Casarabe Culture based upon a land cover dataset that we produced when developing our agent-based model. The quantity of each land cover type was estimated using the ‘Calculate Geometry’ tool in ArcGIS 2.7.0. To produce k, the amount of available land (L_T_) was divided by the land required per person:


$$k=\frac{L_{T}}{\left(L_{PM}+L_{PF}\right)}$$ (7)

*Per capita* maize consumption was estimated based upon the calorific requirements proposed by the WHO for adults aged 18−30 [67]. Following the strong likelihood that maize was their staple crop [20,21,54,68,69], we assume it supplied two thirds of their dietary requirement [22,70], deriving a demand of 178.1 kg person^-1^ yr^-1^ [71,72]. We also derive a fuelwood demand of 700 kg person^-1^ yr^-1^ based upon the consumption of the Tsimane Indigenous group [73], which falls within the expected demands for other groups in the tropics [74]. As no reliable estimates are available for the *per hectare* productivity of maize in the savannas of the LM, we calculated carrying capacity using a range of different maize productivity estimates (300–1800 kg ha^-1^). Information on the parameter values selected for the *per hectare* productivity of fuelwood can be found within the supplementary information (S2 File). Under the scenario where maize is solely cultivated in the open savannas, the minimum k produced between these two resources is selected. Where maize is cultivated in forested areas, the land is optimally distributed according to *per capita* resource requirements. If maize can be cultivated in both, we assume that the Casarabe Culture preferentially cultivated maize in the savannas before encroaching into the forest. A full range of assumptions and calculations for our carrying capacity estimates can be found within the supplementary information (S3 File).

### Agent-based modelling: moundsim population

Our agent-based model, *MoundSim Population*, builds upon a previous model that we created to explore the environmental impacts of the Casarabe Culture (*MoundSim LandUse*), which will be published separately. A full ODD + D (Overview, Design Concepts, Details and Decision-making) description of *MoundSim Population*, including these base mechanics, is provided within the supplementary information (S4 File) [75,76]. Here, we explicitly focus on the aspects of *MoundSim Population* that differ between the two models. *MoundSim Population* has been developed and implemented in NetLogo Version 6.3.0 [77]. The full model code will also be made available on GitHub (https://github.com/JoeHirst-Reading/MoundSim_Population.git).

Similar to our prior model, *MoundSim Population* produces a virtual representation of our study area, comprised of a 657x764 grid of hectare-sized land patches. The model landscape is inhabited by agents, which collectively form an artificial recreation of the Casarabe Culture. Human behaviour is implemented as a set of simple, logical rules guided by the principles of bounded rationality [78,79], which are executed by these agents. As our knowledge of the Casarabe Culture is limited, we inform and parameterise these behavioural rules using ethnographic data sourced from modern indigenous groups [73,80]. The primary agents of interest are intended to represent household units of adults and children, each requiring a sufficient quantity of five different resources to survive: Maize; Foraged Tree Crops; Fuelwood; Palm Leaves; and Animal Protein. Their encoded behaviour enables them to obtain these resources by claiming and modifying land patches on the model landscape. Each household is assumed to reside atop a mound settlement, implemented within the model as a second agent-type to which the households are linked. Each timestep equates to a year, with the model being run for 1000 timesteps to match the known 1000-year occupation period of the mounds [23–25].

Upon initialising the model, 10 settlement agents spawn at suitable locations on the model landscape, each with an attached number of households. The number of agents at startup is intended to reflect the population density at which hunter-gatherers become less mobile [81] because our model assumes the Casarabe Culture developed through demographic expansion. *MoundSim Population* allows both the total population and the number of adults/children within each household to grow and change during a simulation. Every household possesses two variables to track the number of adults/children inhabiting it, with each individual represented as a number reflecting their year of birth. Over time, these individuals age, can start households of their own, produce children, and die.

During each timestep, new households are created as children age, and start families with other eligible individuals from the same settlement. In our experiments below, a child is considered eligible once they reach 16 years of age [73,81]. Households that contain two adult inhabitants can produce new children, with the chance dependent on a user-defined value, controlled by a slider on the model interface (b_h_):


$$Prob(Success)=u(0,1.00)\leq b_{h}$$ (8)

Any new child is added to the children list variable of the household that spawned it. Households can only produce a child if the younger adult, assumed to be female for simplicity, is less than 50 years old [82]. Age-related mortality is handled at the individual level, with the chance of death being age-specific (Mo), based upon data collected from 18 Tsimane villages [83]. For simplicity, all individuals aged 80 and older are assumed to die:


Prob(Death=u(0,1.00)<=Mo (9)

Another defining feature of *MoundSim Population* is that households can move between existing settlements, as well as start new settlements of their own. In each timestep, there is a chance for an active settlement (defined as possessing at least one member household) to experience a migration event depending on a user-defined value (Mi_u_):


$$\begin{array}{c} Prob(Success)=u(0,1.00)\leq min\left({Mi}_{u}*\left(\frac{P_{st}}{{cap}_{t}}\right)\;,\left(2*{Mi}_{u}\right)\right) \end{array}$$ (10)

This chance is modified based upon the population of the home settlement (P_st_) relative to its current capacity (cap_t_), which increases over time as the mound is assumed to be constructed. While it is possible for the settlement’s population to exceed this capacity, doing so further increases the chance of a migration event occurring. Migration events are only possible if the settlement population exceeds half of its current capacity, and must involve at least five households to occur.

A migrating household group can choose either to join an existing settlement or to start their own. They can only join an existing settlement with spare capacity, and can only start a new one if there are fewer than a user-defined number of active settlements within the distance they are willing to travel. Should they fail to meet one of these criteria, they may change their strategy. If neither requirements are met, the event itself will fail. If a migrating group seeks to join an existing settlement, they will calculate a habitability score (H_sc_) for all eligible candidate settlements within the range they are willing to migrate. If starting their own settlement, they will instead calculate a suitability score (S_sc_) for a subset of available land patches. The optimal location is selected based on the following calculation:


$$H_{sc}={Pd}_{sc}-{Cr}_{sc}+{Pop}_{sc}-D_{sc}+{Di}_{sc}$$ (11)

$$S_{sc}={Pd}_{sc}-D_{sc}+{Di}_{sc}$$ (12)

Both scores are comprised of multiple distinct components, each of which can be user-weighted to determine their relative contribution to overall utility. The first component, Pd_sc_, reflects the location’s productivity. Locations are considered more productive if they lie atop the fertile sediments deposited in the southeastern LM during the late Holocene [19]:


$${Pd}_{sc}={Pd}_{w}*(100*Pd)$$ (13)

The second and third components (D_sc_, Di_sc_) are distance related, changing utility scores relative to their distance from the migrating group’s home settlement:


$$D_{sc}=D_{W}*100*\frac{D^{2}}{{R_{m}}^{2}}$$ (14)

$${Di}_{sc}={Di}_{W}*100*\frac{D^{2}}{{R_{m}}^{2}}$$ (15)

The final components (Pop_sc_, Cr_sc_) either increase or decrease utility based upon the population of the candidate settlement relative to its current capacity:


$${Pop}_{sc}={Pop}_{W}*100*\frac{{p_{st}}^{2}}{{{cap}_{ct}}^{2}}$$ (16)

$${Cr}_{sc}={Cr}_{W}*100*\frac{{p_{st}}^{2}}{{{cap}_{ct}}^{2}}$$ (17)

Once a suitable site has been chosen, the migrating group will abandon their existing territory, leave their parent settlement, and attach themselves to the new one.

### Model experiments

We conducted experiments on *MoundSim Population* to explore the growth and maximum size of agent populations under a range of assumptions. To thoroughly explore our model’s parameter space, we employed a Latin hypercube sampling framework to identify 40 optimally spaced parameter combinations (Table 2) [84,85]. Each of these combinations was tested against agents that were programmed to prefer cultivating in elevated areas, but were also dissuaded from claiming land harder to clear of vegetation. 50 simulation runs of 1000 years were performed for each configuration to account for any potential variability in the resulting agent populations (total: 2000 runs).

**Table 2:** Parameter combinations used in MoundSim Population Experiments. The full range of parameter combinations for each configuration can be found within the supplementary information (S6 File).

Continuous Variables	Minimum	Maximum
max-set-density	1.37	17.96
mig-dist-cost	0	1
mig-lobe-bonus	0	1
mig-pop-bonus	0	1
mig-pop-cost	0	1
mig-dist-bonus	0	1
migration-distance	11.38	196.65
migration-rate	0.01	0.20
new-settlement-prob	0.11	0.89
prob-household-birth	0.12	0.30
protein-modifier	0.06	0.40
settlement-base-capacity	23.22	492.56
start-population-modifier	0.50	1.50
yr-10%	13.52	197.04
**Boolean Variables**	**Case 1**	**Case 2**
forage-die	TRUE	FALSE
forest-restrict	TRUE	FALSE
fuelwood-die	TRUE	FALSE
intentional-agroforestry	TRUE	FALSE
lobe-restrict	TRUE	FALSE
maize-die	TRUE	FALSE
palm-die	TRUE	FALSE
protein-die	TRUE	FALSE
**Case Switch Variables**	**Options**	
land-for-cultivation	Forest Only	
	Savanna Only	
	Forest and Savanna	

A parameter combination was considered ‘successful’ and thus warranted further investigation if, across the 50 simulation runs, it produced an average number of settlements similar to those in the real landscape during at least one timestep. We defined this number to be within 20% of the 119 mounds identified within our study area (95–143 settlements) [17]. Crucially, settlement agents did not need to be active at the end of a simulation run for the configuration to be considered successful.

**Fig 2:**
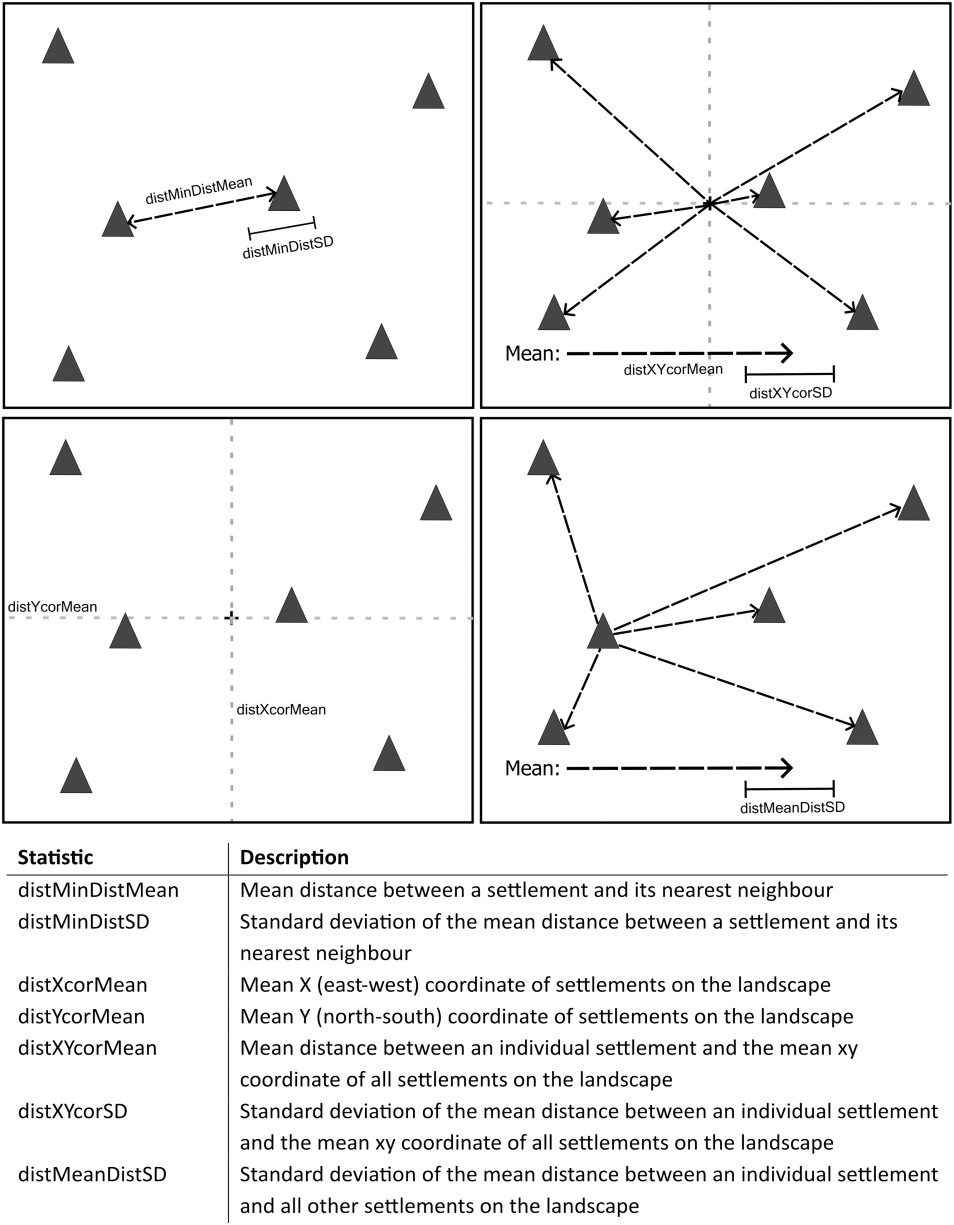
Graphic displaying the spatial components incorporated into our distance metric. These metrics include: the mean and standard deviation of the distance between a settlement and its nearest neighbour; the mean x (east-west) and y (north-south) position of settlements, the standard deviation of the mean distance between settlement agents and the mean xy position of mounds; and the standard deviation of the mean distance between a mound and all other mounds on the landscape.

To investigate which ‘successful’ parameter combination most accurately recreated reality, we compared the spatial configuration of generated settlement agents to mounds on the real landscape. We produced a distance metric incorporating a variety of spatial statistics, such as the mean and standard deviation of the distance between settlements, to aid in this comparison. The full range of statistics considered within this metric are described within Fig 2.

## Results

### Architectural energetics

Our minimum population estimates for the Casarabe Culture, as generated by the architectural energetics approach, indicate that an average workforce of between 4500 and 12,000 people was needed to construct the earthworks of the southeastern LM (Table 3). The majority of these estimates fall between 5000 and 8000 people, only exceeding this under the assumption of very low excavation rates (<0.5 m^3^ day^-1^). These estimates account for all the mounds, causeways, and canals currently mapped within our study area, revising upwards of the previous 20,000,000 m^3^ earth estimated to be necessary for their construction [17] to 49,250,000 m^3^. Approximately three quarters of this value (36,000,000 m^3^) is attributed to mound construction, but the largest increase was associated with the causeway-canal network, which more than doubled to account for infilling and the identification of new earthworks. As the volumetric estimates are based on the mounds for which DEM/LiDAR data is available, they also incorporate any structures which form part of the main mound platform (e.g., pyramidal structures). However, while this also includes other identified structures (e.g., polygonal enclosures [17]), note that (i) their presence varies between mounds and (ii) our study area may still contain as-yet unidentified earthworks. For this reason, and given the incomplete mapping of the causeway-canal network (Fig 1), these estimates should be treated as conservative (Tables 3 and 4).

**Table 3:** Architectural Energetics calculations to estimate the size of the labour force and population of the Casarabe Culture. This includes the number of people needed to excavate and transport the earth needed to build each feature type, as well as total labour force and population estimates.

Excavation Rate (m^3^ day^-1^)	Total Canal/Causeway Excavators	Total Mound Excavators	Total Canal/Causeway Transporters	Total Mound Transporters	Total Labour Force	Workforce: Population Ratio:			
						**1:2**	**1:3**	**1:4**	**1:5**
1.4	521	1,460	22	2,783	4,786	9,571	14,357	19,143	23,929
1.3	561	1,572	22	2,783	4,938	9,876	14,814	19,752	24,690
1.2	608	1,703	22	2,783	5,116	10,232	15,348	20,463	25,579
1.1	663	1,858	22	2,783	5,326	10,652	15,978	21,304	26,630
1.0	729	2,044	22	2,783	5,578	11,156	16,734	22,312	27,890
0.9	810	2,271	22	2,783	5,886	11,772	17,658	23,545	29,431
0.8	912	2,555	22	2,783	6,271	12,543	18,814	25,085	31,357
0.7	1,042	2,920	22	2,783	6,767	13,533	20,300	27,066	33,833
0.6	1,215	3,406	22	2,783	7,427	14,854	22,280	29,707	37,134
0.5	1,458	4,088	22	2,783	8,351	16,702	25,053	33,405	41,756
0.4	1,823	5,110	22	2,783	9,738	19,475	29,213	38,951	48,688
0.3	2,431	6,813	22	2,783	12,049	24,097	36,146	48,194	60,243

**Table 4:** Architectural Energetics calculations to estimate the number of person days needed to construct the earthworks of the southeastern LM.

Total Person-days		Value	Metric
**Minimum**	**Excavation**	36,548,467	person-days
**Maximum**	**Excavation**	154,025,682	person-days
	**Transport**	50,489,047	person-days
**Minimum**	**Canals/Causeways**	10,013,452	person-days
	**Mounds**	77,024,062	person-days
	**Total:**	87,037,514	person-days
**Maximum**	**Canals/Causeways**	40,905,636	person-days
	**Mounds**	163,609,093	person-days
	**Total:**	204,514,729	person-days

Assuming a workforce: population ratio of between 1:2 and 1:5, our calculations indicate the Casarabe Culture reached a minimum population of between 9000 and 60,000 people within our 5020 km^2^ study area. Most of our results vary between 10,000 and 30,000 people, the latter threshold only being exceeded if excavation rates are low (<0.7 m^3^ day^-1^) and if we assume that there are 4–5 dependents per worker. Assuming that the mounds are simultaneously active, this would equate to an average settlement population of between 120 and 200 people. Due to the numerous assumptions made within our energetics approach, these results should not be interpreted as precise; rather they should be viewed as order-of-magnitude approximations for the population size of the Casarabe Culture. Additionally, while these calculations assume a constant amount of earth was excavated and transported during each workday, the Casarabe Culture almost certainly grew in size over time. As such, the estimates quoted in Table 3 should be treated as conservative relative to the peak population actually reached.

### Maximum carrying capacity

Out of our initial 5020 km^2^ study area, approximately 2.65% (133 km^2^) is classified as water unsuitable for either fuelwood extraction or maize cultivation, and has thus been excluded from our carrying capacity estimates. From the remaining terrestrial land (4887 km^2^), approximately 39.9% (1950 km^2^) is classified as forest and 75.6% (3694 km^2^) is located within daily walking distance (7 km) of one of the Casarabe Culture’s settlement mounds. In this restricted range, the percentage cover of forest reduced to 35.2% (1300 km^2^). Our maximum carrying capacity estimates assume that all of the land suitable for each of these activities was fully utilised, factoring in the required fallow period for maize cultivation. These estimates suggest that the southeastern LM could have sustainably supported between 60,000 and 350,000 people (Table 5). While these figures decrease to between 41,000 and 225,000 if the available resources were restricted to those within daily walking distance of a settlement mound, they still imply that the Casarabe Culture was capable of sustaining a large sedentary population on the basis of potential maize and fuelwood supplies.

**Table 5:** Maximum carrying capacity population estimates for the Casarabe Culture based upon maize production and the sustainable extraction of fuelwood. Estimates are provided in number of people. Upper table calculates estimates for all resources within our study region. Lower table calculates estimates for all land within daily walking distance (7 km) from all mounds on the landscape.

Carrying Capacity assuming all resources can be exploited			
Maize Productivity (kg/ha)	Forest Maize Cultivation	Savanna Maize Cultivation	Mixed Maize Cultivation
300	61,734	114,214	154,839
600	104,209	228,428	261,371
900	135,220	334,019	334,019
1200	158,857	334,019	334,019
1500	177,470	334,019	334,019
1800	192,508	334,019	334,019
**Total Area**	4887	km^2^	
**Forest**	39.87	%	
**Carrying Capacity assuming resources are restricted to within daily walking distance (7 km)**			
**Maize Productivity (kg/ha)**	**Forest Maize Cultivation**	**Savanna Maize Cultivation**	**Mixed Maize Cultivation**
300	41,163	93,081	117,040
600	69,484	186,161	197,566
900	90,161	222,717	222,717
1200	105,922	222,717	222,717
1500	118,333	222,717	222,717
1800	128,360	222,717	222,717
**Accessible Area**	3694	km^2^	
**Forest %**	35.17	%	

The extent to which each of these resources constrained the Casarabe Culture’s population size depends upon the assumed productivity of maize. Using a cultivation model with a 3-year cultivation period and 10-year fallow period, similar to modern indigenous groups [65,86], each individual member of the Casarabe Culture would have required between 0.43 and 2.57 hectares of land for maize cultivation and 0.58 hectares of forested land for fuelwood production. Given that the land required *per capita* to produce fuelwood is normally smaller, it only becomes the primary resource limiting population size under specific conditions. These conditions include scenarios where maize productivity is high (>900 kg ha^-1^) or if members of the Casarabe Culture were forced to cultivate maize solely in forested areas, forcing them to choose which resource to produce.

Our results emphasise the substantial benefits of cultivating in the open savannas; 50% reductions in carrying capacity are recorded when maize is grown solely in forested areas. This reduction is driven by the mutually exclusive practices of maize monoculture and fuelwood extraction from the same parts of the landscape. By contrast, strategies that exploit the open savanna are able to support far greater populations because maize can be cultivated in areas where fuelwood extraction is impossible. Mixed cultivation strategies are particularly effective, as they provide further flexibility if the system is maize-limited. At maize productivity levels of 300 and 600 kg ha^-1^, approximately 66 and 33.3% of forested areas respectively remain unused if only the savannas are used to cultivate maize (41.6 and 83.1% respectively when cultivation is restricted to within walking distance). By encroaching into forested areas, mixed strategies are able to increase the population capacity by up to 35.6% for the entire landscape, and 25.7% within walking distance of the mound settlements. When maize productivity exceeds 900 kg ha^-1^, no difference is observed between savanna and mixed strategies because population becomes fuelwood-limited.

It should be noted that these carrying capacity estimates reflect a theoretical maximum population that can indefinitely be sustained. As such, it is possible that the Casarabe Culture could also have temporarily exceeded the capacities quoted in Table 5.

### MoundSim population

**Fig 3:**
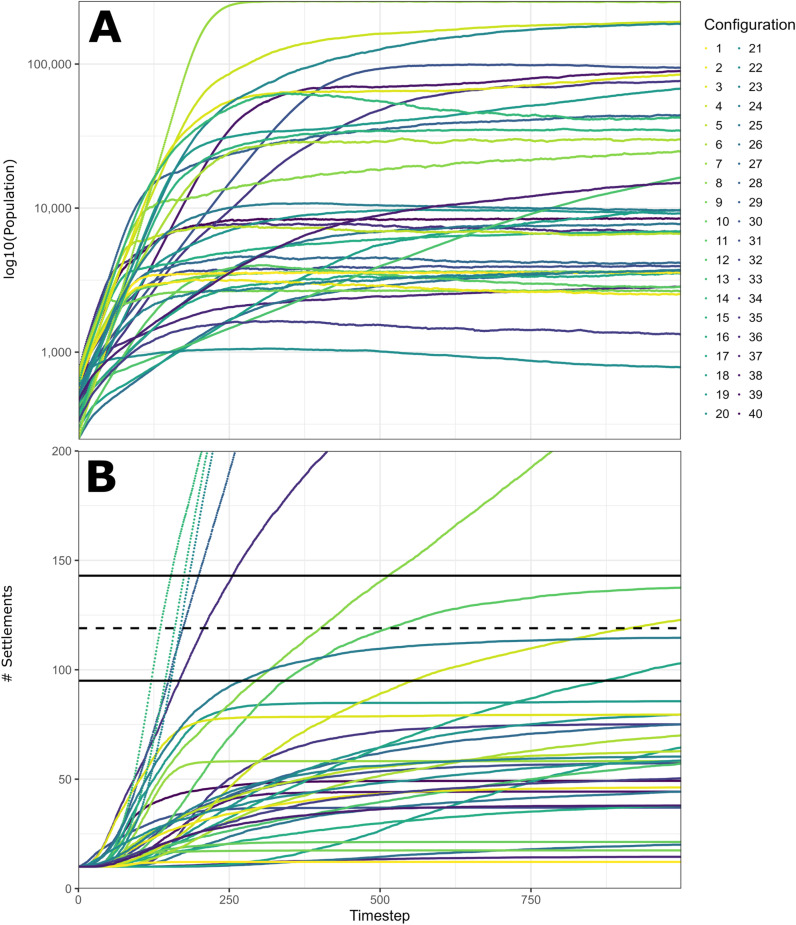
Graphs displaying the outputs of our agent-based model, MoundSim Population. (A) Graph displaying the average population estimates during each timestep for parameter combinations tested in MoundSim Population. Each estimate reflects an average of 50 simulation runs. (B) Graph displaying the average number of settlements during each timestep for parameter combinations tested in MoundSim Population. Each estimate reflects an average of 50 simulation runs. Dashed black line reflects the identified number of mounds in the southeastern LM. Solid black lines reflect the 20% settlement threshold required for parameter combinations to be considered successful.

The 40 parameter combinations generated by our Latin hypercube sampling framework produced a wide range of maximum population estimates. The lowest value (configuration 21) reached just 1000 people, far below the minimum workforce size produced by our energetics estimates. By contrast, four combinations (configurations. 13, 20, 32, and 39) could not be completed because their households faced so few demographic restrictions that their population rapidly exceeded 500,000. Of the combinations that could be completed, the largest (configuration 7) produced an estimate of 270,000, reflecting a hundredfold increase compared with the starting population. The majority of combinations produce populations ranging between 5000 and 100,000 people, depending upon which resources are limited (Fig 3A; also see S7 File). The most restrictive resource was animal protein, which rarely allowed populations to exceed 10,000 even though households never relied on it for more than 40% of dietary requirements. By contrast, simulations restricted solely by foraged tree crops regularly exceeded populations of 100,000.

Irrespective of the restricting resource(s), every parameter combination exhibited a similar pattern of population growth, characterised by expansion for the first few hundred timesteps, before transitioning to follow a logistic growth pattern. This sometimes culminated in the establishment of a population equilibrium, but the time taken to reach it varied substantially. In many cases (e.g., configuration 7), the population began to stabilise within centuries. However, others still continued to exhibit consistent growth even after 1000 timesteps (e.g., configuration 19). Some ensemble members would even overshoot, reaching a population peak, before subsequently exhibiting signs of decline. These patterns are driven by multiple interrelated processes that increase pressure on the finite resources available within localised parts of the model landscape. Even when populations were undergoing exponential growth, small numbers of deaths were still recorded due to localised resource shortages (see S7 File). These are further exacerbated under low migration rates (e.g., configuration 18), as households were unable to escape from the highly stressed localities possessing high populations and low resource availability.

Of the 36 parameter combinations that could be simulated, only 10 were able to produce a similar number of mound settlements as compared with the real landscape (Fig 3B). No single factor was consistently found to prevent a combination from achieving this goal. Certain constraints were common among the successful combinations, such as all possessing fertility rates exceeding 0.15, almost all (9/10) being fatally restricted by palm leaf resources, most (7/10) being restricted by fuelwood, and agents never preferring to start new settlements close to their former home. However, these constraints were insufficient to guarantee success, with configuration 6 failing despite possessing all of them. Furthermore, many ‘failed’ combinations still produced a substantial number of settlements, with 13 combinations generating between 50 and 80 settlement sites. However, it remains unclear whether these combinations could eventually reach the threshold, even with substantial additional time, due to the logistic patterns of population growth observed in Fig 3.

**Fig 4:**
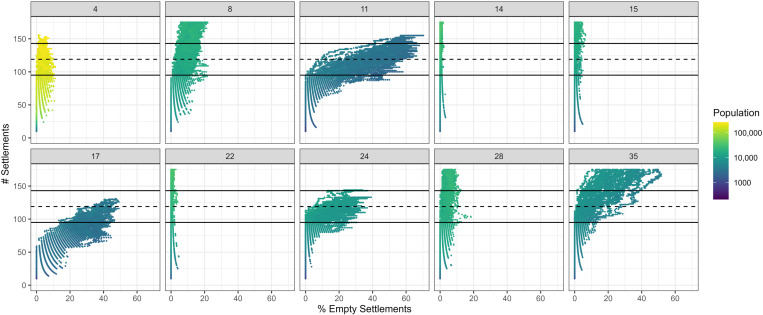
Successful parameter combinations produced from experiments in MoundSim Population. Each point reflects a single timestep within each of 50 simulations conducted upon each configuration. The diagram plots the number of settlements (active and abandoned) during that timestep against the proportion of these settlements which are abandoned (possess no household agents). The colour of each point reflects the number of individuals present on the model landscape.

Successful parameter combinations were observed to produce a wide variety of population estimates, ranging between 3000 and 200,000 people when the number of settlement agents matched the number of mounds on the real landscape (Fig 4). The majority reached populations of between 8000 and 20,000, with only configurations 11, 17, and 4 falling outside this range. Configuration 4 produced populations far larger than any other successful combination, a pattern driven by few resource restrictions (agents were only constrained by maize and palm leaf supplies), low migration rates, and a relative intolerance towards establishing new settlements. Contrastingly, configurations 11 and 17 were heavily resource restricted, such that they could only reach populations of between 3000 and 4000 people.

**Fig 5:**
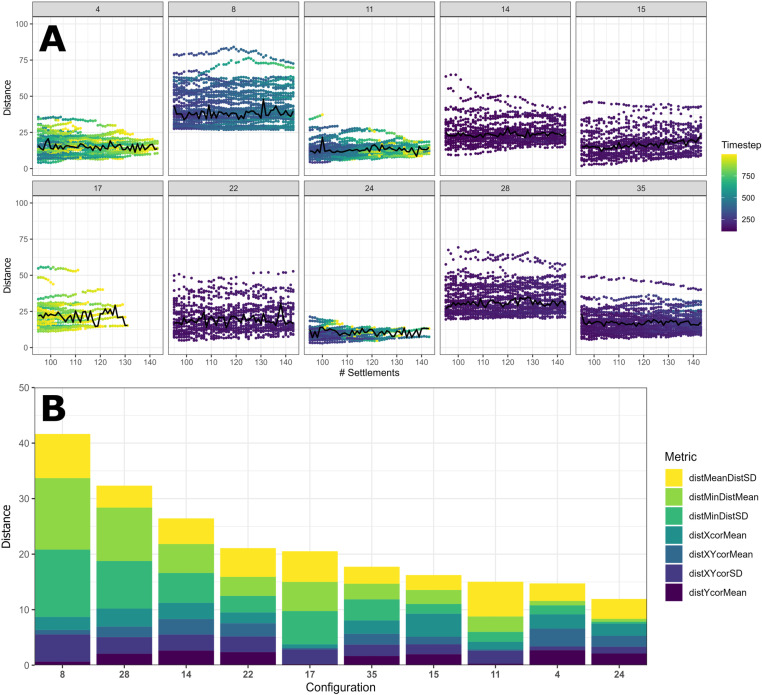
Distance metric scores for each successful parameter combination produced from experiments conducted upon MoundSim Population (A) distance metric scores for each timestep from the 50 simulations conducted for each successful parameter combination. Black line reflects the mean distance score calculated from points which possess the same number of settlements as mounds on the real landscape. (B) mean distance metric scores when each parameter combination possesses the same number of mounds as the real landscape (119). This is broken down by component. distMeandistSD reflects the standard deviation of the mean distance between a settlement and all other settlements on the landscape. distMinDistMean and distMinDistSD represent the mean and standard deviation of the distance between a settlement and its nearest neighbour. distXcorMean and distYcorMean represent the mean x and y coordinates of all settlements. Finally, distxycorMean and distxycorSD represents the mean and standard deviation of the distance between all settlements and the mean position of all mounds on the landscape.

**Fig 6:**
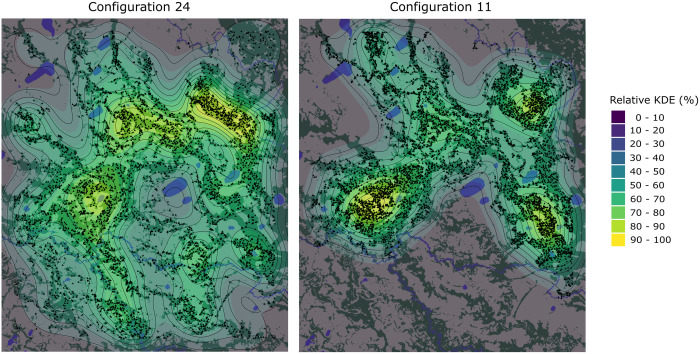
Combined spatial distribution of population density for the 50 runs performed for parameter configurations 24 and 11.

Multiple different strategies enabled households to produce a similar number of settlement agents compared with mounds on the real landscape. Under low agent populations (e.g., configurations 11, 17), the number of households was often insufficient to occupy every settlement site simultaneously. This forced households to jump between active and abandoned sites, resulting in a significant number of empty settlements (empty rate = 20–60%; Fig 4). By contrast, when agent populations experienced few demographic restrictions (e.g., configurations 14, 15, 22, and 28), the number of settlements grew to vastly exceed the number identified on the real landscape, sometimes reaching 500 before stabilising. A third strategy was also observed in configurations 4 and 24, where households prioritised large migration distances and possessed a limited tolerance to any new settlements within the local environment. The differences between these strategies are most evident through their ability to reproduce the spatial configuration of mounds in the southeastern LM. Although the distance scores for each parameter configuration were minimally affected by the number of settlement agents (Fig 5A), they do exhibit substantial differences depending on the strategy executed by household agents (Fig 5B). Parameter configurations where a greater number of settlement agents were tolerated typically produced greater distance scores than other combinations; under these parameter configurations, it was unlikely that the agent population could fully disperse across the landscape before a sufficient number of sites was produced. This likelihood was further reduced if the maximum migration distance was below 7 km, with the extremely short distances in some configurations forcing *MoundSim Population* to produce localised clusters of settlements and leave large portions of the landscape untouched (Fig 6). Combinations that imposed more significant restrictions on the number of settlements more closely reflected the real landscape, even if short migration distances were employed.

Two characteristics were also found to reduce distance scores regardless of the adopted strategy. Restricting new settlement sites to where nutrient-rich fluvial sediments are located [19] was found to almost eliminate the distance component attached to the mean Y coordinate of settlements on the landscape (see configurations 8, 11, and 17). This indicates our model better approximates the spatial configuration of real mounds if the areas away from this fertile sediment are excluded as suitable settlement sites. Furthermore, the two configurations with the largest migration distances (configurations 4 and 24) produced very low scores for the mean and standard deviation of the distance between a settlement and its nearest neighbour. This indicates that larger migration distances more closely approximate the real landscape.

## Discussion

### Minimum population size

Our energetics estimates demonstrate that the earthworks present in the southeastern LM could have plausibly been constructed by a workforce containing as few as 4500 people. Multiple factors contribute to explaining this surprisingly small value. First, while the largest mounds cover over 20 ha and reach up to 20 m high [18], only 119 of these structures have been identified within our 5020 km^2^ study area. For comparison, over 16,000 raised fields have been identified in a region of the northwestern LM less than 1/10^th^ of this size (416 km^2^) [87]. Thus, while each raised field is considerably smaller than a settlement mound [13], the amount of earth mobilised per square kilometre (13,000 m^3^ km^-2^) is significantly higher than in our study area (9800 m^3^ km^-2^). Second, the task of constructing these earthworks was made considerably less labour intensive by building them gradually over the 1000-year period they were occupied [23–25,88]. This occupation period is so long that, even if the earthworks are treated as a community project for which little time is available outside of normal subsistence activities (following [73]), they still would have only required a relatively small workforce. Based on our calculations, and assuming a workforce-to-population ratio of 1:2, it is conceivable that a population of just 9400 could have been responsible for constructing these earthworks. Under this scenario, the Casarabe Culture would have been thinly dispersed across the southeastern LM, with each of the mounds in our study area inhabited, on average, by just 79 people. This echoes previous claims that the culture might have practiced a form of low-density agrarian urbanism [18]. While our results do suggest that a much larger population (up to 60,000) would have been needed if excavation rates were extremely low (<0.5 m^3^ day^-1^), such a scenario is unlikely given the rates recorded at other sites where dense soil was mobilised commonly exceed 1 m^3^ day^-1^ [49,64].

The outputs of *MoundSim Population* show that a sufficient number of settlements could have been produced by fewer people than our minimum energetics estimate of 4500, suggesting that populations as low as 3000 could produce a similar number of settlements to the real landscape. However, these outputs also show that such a low population would be insufficient to inhabit every settlement simultaneously. Instead, household agents were observed to abandon up to 65% of settlement sites, with the remainder being inhabited by between 50 and 60 people. While it has previously been suggested that the mounds were constructed in a spatiotemporally discontinuous fashion [89], inhabited by sedentary communities on a cyclical basis [28], the plausibility of this hypothesis is undermined by the earthworks themselves. The simultaneous occupation of the mounds is clearly demonstrated through them being interconnected by the causeway-canal network [17] and their contemporaneous radiocarbon dates [23–25]. Additionally, it is important to consider the practical benefits of these earthworks. The causeway-canal system, which accounts for one quarter of the mobilised earth, is known to have served an agricultural purpose [17,20]. Modern indigenous groups often arrange themselves into communities larger than the minimum sizes produced by both our energetics model and by *MoundSim Population*, and yet none construct earthworks. Instead, they practice slash-and-burn forest cultivation and avoid the savannas due to poor drainage and weed competition [13,73,80]. Slash-and-burn forest cultivation would have presented significant challenges for the Casarabe Culture, especially given their lack of access to metal tools [90], but it remains difficult to imagine that these challenges exceeded those associated with constructing and maintaining such a large canal network. We therefore consider the notion that an indigenous population of between 3000 and 6000 people was willing and able to construct these earthworks to be unreasonable. Instead, we follow our energetics estimates in arguing the Casarabe Culture grew to a population of at least 10,000. At this size, the outputs of *MoundSim Population* show that more than 70% of settlements were contemporaneously active, providing an explanation for why they would be integrated into the wider causeway-canal system. As shown in configuration 24, reaching a population of 10,000 was also easily achievable within the known mound occupation period [23–25].

Our minimum population estimate of 10,000 people represents a thirteenfold increase on the smallest population estimate currently available for an equivalent area of the LM (750 people) [40]. This exceeds the 0.2–1 people km^-2^ carrying capacity estimates applicable to wider Amazonia, though it should be noted that the population densities of some modern indigenous groups have also exceeded this threshold [26–28]. Our estimates broadly align with the 2 people km^-2^ density proposed by Denevan for the wider LM [38] and, in doing so, vindicate his concerns around the intra-regional variability of population estimates [91,92]. Although his figures were intended to reflect an average population density, they fall on the conservative end of our estimates.

### Maximum carrying capacity and resource restriction

Maximum carrying capacity estimates show that our study area could have plausibly supported up to 334,000 people on the basis of maize and fuelwood production. This value is multiple orders of magnitude greater than other published density estimates applicable to the region, and suggests that the southeastern LM was capable of supporting a dense sedentary population prior to European Contact [18,93,94]. Nonetheless, we stress that the maximum carrying capacity of a given environment does not inherently equate to the equilibrium population density actually reached there [95,96]. This notion is supported by the results produced by *MoundSim Population*; of the 40 parameter combinations tested during our experiments, only eight consistently generated population estimates exceeding 100,000 people. Just one of these (configuration 4) also produced a final number of settlements approximating the number of mounds identified on the real landscape. The rarity of our model runs generating populations that exceeded less than one third the size of those produced by our carrying capacity approach, especially given that nearly all terrestrial land patches (97.7%) were within 3 km of a site designated suitable for settlement, suggests that this discrepancy was significant for the Casarabe Culture.

Myriad factors can prevent the population density from reaching this theoretical maximum limit. For instance, we recorded 33% relative reductions in carrying capacity simply by restricting the available resources to those within daily walking distance of the settlement mounds (7 km) [65]. As the mounds are unevenly distributed across our study area, doing this renders 24.4% of the terrestrial land surface inaccessible. It also causes a 4% relative reduction in forested land, which is more valuable given that it can be used both for cultivation and fuelwood extraction. Environmental constraints and preferences can also widen this discrepancy; the clear benefits of open savanna cultivation [20] are exemplified by the doubling of carrying capacity when the savannas are farmed, compared with when cultivation is restricted solely to forested areas. Conversely, forest cultivation forces the choice between exploiting land for agriculture versus fuelwood, given that at least some degree of forest clearance, or thinning, would be necessary due to maize being shade intolerant [97].

It is possible that the values we used to parameterise both our carrying capacity model and *MoundSim Population* may simply underestimate resource supplies on the real landscape. For example, while we calculated palm leaf supplies in *MoundSim Population* based upon the abundant Motacú palm (*Attalea phalerata*) [94,98], other palms found on this landscape, including the spiny palm Murumuru (*Astrocaryum murumuru*), have been used for construction on rare occasions [99] (though this palm is far more commonly exploited for its fruit [73,80,100]). Equally, we must recognise that the real Casarabe Culture may have managed their resources more efficiently than is implemented in *MoundSim Population*. While our model incorporates intra-settlement resource exchange [101], it is likely that the causeway-canal network facilitated inter-settlement trade, which may explain some of the discrepancy between carrying capacity and the actual population reached.

Nonetheless, it is also important to consider our estimates in relation to logistical constraints. Assuming each mound structure possessed an average 5.5-ha surface area [17], the settlement mounds in our study area could collectively provide a population of 334,000 with just 19.6 m^2^ of space *per person*. It is physically possible to live within such a confined space, but there would be little reason for the Casarabe Culture to impose such severe restrictions on themselves instead of further expanding their mounds, particularly given the available labour force. When this is considered alongside the variety of factors that may have prevented the Casarabe Culture from reaching ‘maximum’ resource use efficiency, we believe it is unlikely that this culture’s population exceeded 100,000, and probably reached no more than 50,000 people. Such a population is far more consistent with the existing earthworks, as the mounds within our study area could collectively provide 65.5–130.9 m^2^ person^-1^ of space. Most of the mound’s surface would be occupied under this scenario, but it would also provide sufficient space for communal functions, such as the ceremonial pyramid observed atop some mounds [17,18]. Our model also shows that such a population could be reached within the known occupation period. We therefore consider 50,000–100,000 people to be a reasonable maximum population range for our 5020 km^2^ study area.

Representing a density of between 10 and 20 people km^-2^, our maximum population estimates broadly align with Clark Erickson’s claim that the medium-sized settlement mound close to Ibiato village was inhabited by between 500 and 1000 people [32]. These values are also similar to the estimates of 6–12.5 people km^-2^ proposed for the pre-Columbian settlements of the Upper Xingu [11] and fall on the lower end of population estimates made for the earthworks produced by the Marajoara culture at the mouth of the Amazon [12,102]. More widely, the Casarabe Culture’s maximum population is similar to, and even potentially greater than, that of the Mississippian culture of greater Cahokia [103,104], but is much lower than that of the regional-scale urban polities developed by the Maya in Mesoamerica and Greater Angkor in Cambodia, where population density could attain several hundred people per square kilometre [105,106].

### Spatial configuration

The outputs of *MoundSim Population* show that the Casarabe Culture could have employed multiple migration strategies to produce the number of mound settlements seen on the real landscape. These include small, cyclical reoccupation by sedentary communities, rapid expansion driven by a large, unrestricted population, and a gradual deterioration in the creation of new settlements as the settlement density becomes saturated. The first strategy can immediately be excluded given the highly interconnected nature and contemporaneous radiocarbon dates of the mounds [17,23–25]. However, the other strategies cannot be discounted based on the data presented here; both approaches successfully reproduced the number of real settlements, aligning with the concept of equifinality [107,108]. Nonetheless, the distance metric we used to evaluate each successful combination enable us to identify which assumptions produce a more realistic recreation of the southeastern LM.

Chief among these assumptions is migration distance. Although the average distance between a mound and its nearest neighbour on the real landscape is just 2.69 km [17], the two parameter combinations which most accurately reproduced these spatial characteristics (configurations 4, 24) possessed: (i) the largest migration distances of all successful combinations (13.0 and 19.5 km respectively); (ii) agents that prefer to migrate larger distances from their home settlements; and (iii) growth strategies intolerant of large numbers of settlements within the local environment. Combining these characteristics produced average distances of 2.25 and 2.36 km respectively. By contrast, shorter migration distances produced much lower values (lowest = 0.8 km), even if the agents preferred migrating large distances. From these observations, we infer that even though the mounds are densely packed, their inhabitants still viewed retaining sufficient land within their local environment as an important priority. This is unsurprising given even small indigenous communities still operate several kilometres away from their home settlement [73,80]. Thus, the outputs of *MoundSim Population* imply that contrary to the strategies characterised by rapid, unrestricted expansion, the mounds in the southeastern LM are likely to have already reached their maximum density. This notion is particularly striking, as previous mapping shows a number of mounds being located in close proximity to one another, with some just 420 m apart [17,18]. Such a small distance raises questions around whether these mounds were simultaneously occupied [89], or why the Casarabe Culture chose to start building a new mound despite an abandoned one being in such close proximity. Our model treats each mound as an individual settlement, but it is also possible that multiple habitation mounds may form part of a single, larger settlement complex.

Another important characteristic identified within our experiments is whether settlements were restricted only to spawning on the fertile sediments deposited during the late Holocene [19,109]. Under parameter combinations allowing agents to spread unhindered, the mean Y (north-south) position of settlements was 5 km further south than when restricted, causing the ‘distYcorMean’ component of our distance score to quadruple in value (restricted distance from true value = 1.13 km (n = 3), unrestricted distance 5.66 km (n = 7) respectively). The settlements also dispersed more widely when unrestricted, quintupling the ‘distXYcorMean’ component score (restricted difference = 0.4 km (n = 3), unrestricted distance = 2.54 km (n = 7) respectively). On this basis, our results corroborate proposals that the Casarabe Culture developed by exploiting these fertile sediments [110]. The spatial configuration cannot be explained through elevation because the southern part of our study area possesses more than sufficient elevated land to enable the Casarabe Culture to settle (see Fig 6). Neither can lower forest cover because, although the proportion of forested land is lower in the south, household agents were restricted to settling in forested locations during 60% of successful parameter combinations. This does not imply that the Casarabe Culture was bound by insurmountable environmental limitations [1,5,6], especially given that some mounds have been found outside the boundaries of the fertile deposits [18]. However, it does suggest that this culture considered the fertile sediment to be an important factor in determining where to settle.

Although we cannot determine the migration strategy used by the Casarabe Culture to settle this landscape, the outputs of our agent-based model suggest that the thousand-year occupation period of the southeastern LM [23–25] was more than sufficient to enable this culture to develop into a large, sedentary society. Household agents were easily capable of producing a sufficient number of settlements to match the real landscape and, in several model runs (e.g., configuration 14), were able to produce enough to surpass this value multiple times over. This prompts the question of whether the final number of settlements on the landscape may have been curtailed in some way. As shown by *MoundSim Population*, internal behavioural factors—such as the desire to migrate larger distances—could certainly have prevented the establishment of settlement mounds. The highly integrated nature and close spacing of the Casarabe Culture’s mounds [17] clearly highlights the society’s emphasis on internal cooperation, but this does not imply that new settlements in close proximity to existing ones would be tolerated, nor does it rule out the possibility of inter-settlement conflict. The moat and rampart features identified around some of the Casarabe Culture’s mounds [18] indicate that, at the very least, defensive structures were necessary to deter conflict.

It is also important not to overlook the potential impacts of external forcing factors. While beneficial for resource exchange, the Casarabe Culture’s integrated canal-causeway network [17] would have simultaneously made them more susceptible to epidemic-related depopulation events than other, more dispersed pre-Columbian communities [111,112]. Additionally, as the development of this culture spans the medieval climate anomaly [113,114], it is quite conceivable that climate perturbations either directly or indirectly influenced demographic trends [115,116]. The potential impacts of such drivers have not yet been incorporated into our agent-based model, and is something we plan to address in future work.

### Numbers from nowhere: A note on model limitations

The problems associated with generating past population estimates are well-known and documented [91,92,117,118]. As with all such attempts [4], estimating the Casarabe Culture’s population required making numerous assumptions about their behaviour and activities. Some of these assumptions are reasonable even with the limited availability of empirical evidence; for example, the rarity of stone tools [47] and lack of domesticated animals in lowland South America [119,120] makes it safe to assume any earth mobilised by the Casarabe Culture was excavated by wooden digging stick and transported by hand. We can also reasonably estimate the efficiency of these activities because their work rate is very likely to fall within the existing range of values collected from experimental archaeology studies [49]. In other cases, however, we are forced to make assumptions for which no supporting evidence is available. For example, implicit within *MoundSim Population* is the assumption that the Casarabe Culture’s growth and spread was driven by demographic expansion. The 1000-year occupation window employed within this study is based upon the radiocarbon dates obtained from just three mounds [23–25] and the regional population size prior to this, between 2000 BCE and 400 CE, is even less certain given much of the region remains unexplored archaeologically [19,109]. A new range of possibilities opens up depending upon the population size during this interim period. The presence of numerous villages would imply the practice of mound building may have spread through cultural diffusion rather than demographic expansion. Alternatively, the absence of prior settlements and the rapid establishment of the Casarabe Culture would point towards them having migrated from elsewhere.

We raise this example to emphasise that the population estimates presented here should be treated as just an initial step to determining the true size of the Casarabe Culture. Much remains to be learned about how this culture operated in terms of practice and behaviour, which could further constrain our models. Unknown factors, such as the initial population size, can substantially influence the resulting trends in population growth, and further empirical research should be undertaken to fill these gaps. For example, it is important to identify how much of each mound was occupied at any one time, how densely packed the people were on these structures, and whether the existing window of mound occupation is truly representative of earthworks in the wider area [23–25]. Moreover, our agent-based model does not yet attempt to recreate the four-stage mound settlement hierarchy identified from recent LiDAR scans [18], additional evidence that may help to determine how the Casarabe Culture spread over time. Nonetheless, despite these acknowledged limitations and caveats, our multifaceted approach has produced the first ever set of plausible, supported population estimates for this culture. We have explicitly defined and quantified the assumptions associated with our methods throughout this article to ensure transparency, and we recommend that any future studies estimating pre-Columbian population sizes are similarly upfront with their inherent assumptions and limitations.

## Conclusions

Our multifaceted modelling approach has enabled us to tentatively constrain the population of the Casarabe Culture to between 10,000 and 100,000 people within a 5020 km^2^ area of Amazonian Bolivia. This reflects a substantial improvement upon the existing range of regional estimates (Table 1) that span four orders of magnitude (<1000 to >100,000 people) for our study area [37,40]. Representing a density of between 2 and 20 people km^-2^, our population estimates are similar in magnitude to the claims made for other complex pre-Columbian Amazonian earthmoving cultures, such as those in the Upper Xingu [11] and the Marajoara culture at the Amazon’s mouth [12,102]. The latter comparison is especially noteworthy given that the Marajoara are similarly known for constructing large mound earthworks within a forest-savanna mosaic environment [12], though their subsistence strategy, focusing on aquatic resources, stands in contrast to the maize monoculture practiced by the Casarabe Culture in the open savannas [20,102]. However, while the pre-Columbian population density of the southeastern LM was significantly larger than it is today (<1 person km^-2^) [14], the Casarabe Culture remained an order of magnitude smaller than major pre-colonial tropical forest polities beyond Amazonia, such as the Classic Maya and Greater Angkor [105,106]. In fact, at the lower end of our estimated population range, each of the 119 mounds within our study area would have, on average, been inhabited by fewer than 100 people. This population size is smaller than that of some indigenous villages present on the modern landscape [73], corroborating previous claims that this culture might have practiced a form of low-density agrarian urbanism [18,121].

We reiterate that the population estimates presented within this article represent an initial exploratory step to determining the true population size of the Casarabe Culture. Producing these estimates involved making myriad assumptions about the Casarabe Culture’s behaviour and agricultural practices. Given that only two mounds have been excavated in any detail [23,47,122] and just two palaeoecological records are currently available for the region [20,21], the models presented here will greatly benefit from additional empirical research as it becomes available. It is also important to emphasise that these estimates solely pertain to the confines of our study area (Fig 1). Scholars have highlighted the challenges of applying population estimates calculated for specific regions to broader areas [91,92,123], and it would be particularly inappropriate to extrapolate estimates calculated for unique regions, like the one inhabited by the Casarabe Culture, to other parts of the Amazon basin. The seasonally-flooded forest-savanna mosaic vegetation and younger, fertile sediments characteristic of this location significantly influence the demographic trajectory of the populations inhabiting it [19,109,110], but these features are highly atypical of Amazonia as a whole. Nonetheless, we believe that future studies aiming to estimate pre-Columbian population sizes would greatly benefit from adopting a similar kind of transparent, multifaceted approach to the one employed within this article.

## Supporting information

S1 File SI1 Architectural Energetics. File containing architectural energetics population estimates for the Casarabe Culture.
(XLSX)

S2 File SI2 Model Parameters. File containing the parameters selected for use in MoundSim Population.
(XLSX)

S3 File SI3 Carrying Capacity. File containing carrying capacity population estimates for the Casarabe Culture.
(XLSX)

S4 File SI4 ODD + D: MoundSim Population. File containing an **‘**Overview, Design, Details and Decision Making’ (ODD) description of MoundSim Population.
(PDF)

S5 File SI5 Imported Model Data. File containing details on the spatial data imported into MoundSim Population.
(PDF)

S6 File SI6 LHS Parameters. File containing the parameter values selected as part of the latin hypercube sampling framework used to test MoundSim Population.
(XLSX)

S7 File SI7 Extended Results. File containing extended outputs of MoundSim Population.
(PDF)
