# Supplementary material for: Constraining the population size estimates of the pre-Columbian Casarabe Culture of Amazonian Bolivia
Source: PLoS One. 2025 May 30;20(5):e0325104. doi: 10.1371/journal.pone.0325104 (PMC12124758; doi:10.1371/journal.pone.0325104)
Supplement: S4 File — File containing an ‘Overview, Design, Details and Decision Making’ (ODD) description of MoundSim Population. (PDF) [file pone.0325104.s004.pdf]

ODD: MoundSim Population

Below we provide an ODD (Overview + Design + Detail) description of MoundSim Population [1,2]. This description is amended with the recommendations of Müller et al. [3] to better account for features of the model that are associated with human decision-making.

Purpose

MoundSim Population is an agent-based model that aims to constrain a range of potential population estimates for the pre-Columbian Casarabe Culture in the Monumental Mound Region (MMR) of the Llanos de Moxos (LM), located in Amazonian Bolivia. The few available estimates for this region currently span multiple orders of magnitude [4–7]. Due to the numerous difficulties and limitations associated with each method, some scholars have even resorted to informed guesswork [8,9].

MoundSim Population approaches the problem from an alternative perspective; it places a central focus on the decisions made at the individual level [10,11]. The model functions as a 'virtual laboratory', enabling the user to examine how changes in resource demands, demographic parameters, and agent preferences influence the growth and spread of the agent population in a variety of 'What if' scenarios. By comparing model outputs to population estimates generated from alternative methods (Architectural Energetics, Carrying capacity), as well as known features of the contemporary landscape, the model serves to help constrain the size of the population that once inhabited the MMR. MoundSim Population is implemented in NetLogo Version 6.3.0 [12].

Entities, State Variables, and Scales:

MoundSim Population possesses four agent types: Square land patches, pre-Columbian household units, settlements, and community ties. The model uses imported, georeferenced (GIS) data to recreate a 5020 km^2 area of the MMR, including the 4500 km^2 region studied in Lombardo and Prümers [13]. The simulated landscape consists of a 657x764 grid of patches, each representing one hectare of land. Each patch possesses immutable environmental variables determined by imported data (e.g., Elevation, Productivity). They also possess a variable to determine its state of land cover (land-use), which is initially determined by imported data, but can change over time.

The primary agent of interest within the model represents a pre-Columbian household unit. Each has two variables to denote the number of adult and child inhabitants residing within it. These inhabitants factor into the longevity and resource demands of the overall household. Households are assumed to be permanently situated atop a mound settlement agent, to which they are attached by a community link. While the settlement agent influences household behaviour, community link agents possess no variables of their own and simply exist to connect these two agent types. The households are also characterised by a set of variables that describe their demand for a variety of resources considered necessary by both the pre-Columbian inhabitants of the MMR (the 'Casarabe culture') and members of contemporary Indigenous Amazonian communities (Maize, Foraged Tree crops, Fuelwood, Palm Leaves, and Animal protein).

To acquire the resources necessary for their survival, households possess behaviour to claim, retain, modify, and extract resources from terrestrial patches of land. These processes take place within a user-defined distance from the attached settlement agent, stored in three different variables

(settlement-radius, forage-radius, fishing-radius). When land is claimed, households possess a variable to record and track the identity of the patch (household-territory), with a similar variable used by each patch to track its owner household (owner).

If an insufficient quantity of each resource is produced by the patches within a household's territory, that household can utilise two alternative methods to obtain them. Firstly, they can obtain resources from communal stores supplied by other households that are attached to the same settlement. Secondly, they can extract resources from unclaimed patches within a set radius that are controlled by their settlement (stored in the settlement-owner patch variable). If the household fails to produce a sufficient quantity of resources, the modeller can decide whether it either becomes stressed (discussed below), or dies.

Global parameters are initially used to store imported environmental information (elevation, productivity, land-use) before it is mapped to a number of patch variables. A similar parameter keeps track of imported demographic information that is important in calculating individual mortality. Further parameters keep track of population over time, as well as the number of households that die as a result of resource shortages. Each timestep in the model equates to one year of activity, with simulations running for one thousand timesteps to match current radiocarbon data that indicates the mounds within the central MMR were continuously occupied between 400-1400 CE (Jaimes Betancourt, 2012, 2015; Prümers, 2015:82-87).

Process, Overview and Scheduling

MoundSim Population simulates multiple key processes. Upon initialisation, 10 settlement agents are spawned on the model landscape, with a user influenced number of household agents attached to each. Without user modification (start-population-mod = 1.0) the baseline number of households matches the defined packing threshold noted in Binford (2001; 9.08 persons 100 km^{-2}). The number of households can increase during a simulation as child inhabitants age and marry, starting new households of their own. However, it can also decrease as a result of age-related mortality and resource shortages.

The first procedure executed during each timestep is population increase, during which each household determines whether its eldest child marries and breaks away to form a new household. It is only possible for a settlement to produce new households if at least two of its households contain child inhabitants of sufficient marrying age. This procedure also determines whether households that contain two living adults procreate and produce new children. Children cannot become adults in the same household they were born in.

The second procedure executed simulates the migration and generation of new settlement agents. As settlements grow in population, they might experience migration events, during which a portion of their households leave to either join another existing settlement, or to start their own. This is only possible if the settlement is above 50% population 'capacity', a value that increases as a settlement grows over time. The chance of a settlement to experience a migration event is also directly related to how close it is to population capacity. Migrating households can only join another settlement if there is one within the range they are willing to migrate that is under population capacity. They can only start a new settlement if the number of settlements within this same range falls below a user defined threshold (max-settlement-density). If these criteria cannot be met, they either switch strategy or the migration event itself fails.

The third procedure executed represents the conversion of land. During this procedure, households make decisions around whether and how much additional land to convert for maize-based agriculture. If a household deems its supply insufficient for the following year, it will claim and convert additional land patches. Terrestrial land from anywhere on the landscape can be claimed for resource production, as long as it lies within an appropriate distance range. However, households can only claim and convert a limited number of patches during a single timestep, reflecting limitations on time and labour. This procedure updates the landscape asynchronously to reflect the competition for finite resources, as well as to ensure a patch cannot be owned by more than one agent.

The fourth procedure reflects the production of resources. Patches possess a number of variables to store the supplies of various resources. This procedure allocates a suitable quantity of each resource type to patches within the territory of household agents.

The fifth procedure, decrease population, determines whether household agents have produced sufficient quantities of each resource to avoid becoming stressed, which household inhabitants die, and whether any households disband. Each individual within a household has a chance to die based upon their age, a process informed by imported data. A household will disband if it possesses no more living inhabitants. However, a household may also disband (and its inhabitants may die) due to a lack of resources. Decrease population manages the alternative methods of acquiring resources should a household's territory prove insufficient. If the quantity of resources remains insufficient, it determines whether households become stressed, or disband.

The final procedure, vegetation-growth, accounts for the abandonment of cropland and the regeneration of fallowed land into forest and savanna. Initially, it determines whether a patch is abandoned based on the number of years it has remained in cultivation. Subsequently, the procedure stochastically governs the transition of fallowed land into regenerated savanna or forest based on whether it was forested at the start of the simulation.

Design Concepts

Theoretical and Empirical Background

MoundSim Population has been built to generate and explore a range of 'What if?' scenarios regarding the size of the MMR's pre-Columbian population, as well as the extent and spatial distribution of pre-Columbian land-use within the region. The paucity of available data makes this aim heuristic by nature; it seeks to explore a range of plausible scenarios rather than attempting to approximate reality [18]. The mechanisms within the model take inspiration from Malthusian theory [19] in that, provided aggregated household demand never exceeds the total quantity of locally accessible resource supplies, reproduction can proceed unhindered. However, total resource supplies are finite and, when insufficient, households may become stressed or die.

These concepts are encoded as rules governing household agent behaviour. Rather than being able to claim, convert, and extract resources from anywhere on the landscape, households are restricted in terms of accessibility. They can only procure resources within a set radius of their attached settlement, can only convert a set number of patches per timestep, and may not select the optimal location for their farms.

Individual Decision-making

Decision-making within the model is conducted at the household level. Households are satisficers that utilise inductive reasoning to determine which and how many patches to convert [20]. This is demonstrated when converting land, as households claim additional patches based on whether the expected supply of resources from their existing territory is sufficient for the forthcoming timestep. They have further preferences for certain environmental characteristics defined by the user, which influences the patches selected for cultivation. However, this comes with restrictions. They can only convert a set amount of land during each timestep, and can only choose between a subset of the patches available to them.

Due to the paucity of available data, few socio-cultural values are considered beyond the assumption of maize as the staple crop [16,21–23], and the households being sedentary, located atop habitation mounds [13,24].

Learning

Learning within the model is simplistic. Households are able to track the average productivity of their land over time, altering their expectations regarding how much of each resource it expects to receive.

Individual Sensing

Households employ various sensing mechanisms to inform their decisions. When cultivating patches, they can discern the essential characteristics of each patch, including whether a patch is owned, its elevation, and its land cover characteristics. They will only attempt to convert unclaimed land, in keeping with the practices of contemporary Indigenous groups. The model, however, does not account for territorial disputes.

When choosing where to cultivate, households are assumed to possess the ability to recognise fallow land, determining when it became fallow by recognising distinctive vegetation characteristics [25]. This is encoded in the model by allowing agents to sense the land-use variable, with agents choosing only to farm areas of regenerated forest/savanna even if abandoned fallow land is available. This assumption is a practical, if simplified, representation of the real world, as humans may recognise fallowed land but not necessarily its owner.

When migrating, households are assumed to be able to accurately determine the population size of candidate settlements relative to their current capacity, the distance to that settlement, and whether the land is productive. However, they are only assumed to know this information within a user-defined radius of their home settlement. They cannot determine the information of settlements outside this radius, and will not attempt to join settlements unless they are within range.

When the children within a household attempt to leave and start a new household, they are assumed to know of other eligible children within the same settlement. However, they do not know of eligible children in other settlements.

Individual Prediction

Prediction plays a crucial role for households in deciding whether to cultivate additional land. This is based on whether households expect to obtain enough resources from patches within their territory to meet their needs for the following year. Their decision relies upon imperfect information, utilising average resource yields derived from productive patches of cropland, agroforestry, and fallow. It is assumed that the pre-Columbian inhabitants of the MMR were able to assess average resource yields and anticipate expected sources of yield reduction such as crop loss. Furthermore, households also

factor in the productivity of land from previous timesteps when making agricultural decisions, giving them a degree of sensitivity to unexpected variability. However, they cannot determine the level of unexpected variability for the current timestep.

Households within the model assume that a single hectare of land can produce an expected quantity of maize. Following patterns observed within the Tsimane population, we assume the MMR's pre-Columbian population consumed this maize throughout the year [26]. Acknowledging limitations in storage options and pest control, the model assumes that any surplus from cultivation cannot be stored interannually. Additionally, households proactively make cultivation decisions, considering a predetermined 15% loss in maize yield as a pre-emptive measure (Ringhofer, 2010).

Interaction

Households within the same settlement collectively engage in direct and indirect interactions that are facilitated by the community tie link. Households may only share resources between members of the same community. All households are assumed to reside on a mound settlement, cultivating within the range determined by the settlement agent. This fosters indirect competition for desirable land patches. Individuals within a household may only marry and start a family with another member of the same settlement. They will also only migrate alongside at least four other households from the same settlement.

Collectives

Each household is compelled to establish a community link to a pre-Columbian settlement collective agent. This ensures every household belongs to a collective, even if that collective contains only the household itself. These collectives play a pivotal role by influencing the ability of member households to convert patches on the landscape, to produce new households, and to migrate. Patches can only be converted within a given range of a settlement. A collective agent can also exist without attached household members (we treat this as an abandoned settlement). Settlement collectives also influence the sharing of resources, and dictate the radius within which agents can extract resources from unclaimed patches. Households only migrate if the total population of the settlement exceeds 50% of its current capacity.

Heterogeneity

Household agents are heterogeneous in that each varies in terms of the number of adults and children inhabiting it, as well as the age of these individuals. This significantly influences whether a household executes parts of the increase population procedure (a new child can only be produced if there are two adults within a household. A household will only attempt to produce a new one if it possesses an eligible child). It also influences the level of resource demand experienced by each household, which plays a key role in claiming and converting additional patches of land.

Stochasticity

Stochasticity is involved in selecting patches for cultivation. Only a random subset of the available patches within range are chosen. The size of this subset is defined by the user, enabling variations in agent knowledge to be tested. The user is also provided a NULL model choice, which selects patches within range entirely at random.

Stochasticity is involved in resource production and vegetation growth. In the former, stochasticity acts to vary production around a mean value defined by constants within the model. In the latter, it acts to vary the rate at which certain patches become reforested/regenerated after abandonment.

The production of new children within a household is managed stochastically, dependent upon whether a household possesses two eligible adults and a user-defined probability. In addition, the model stochastically determines whether a settlement experiences a migration event, as well as whether that event initially involves moving to another existing settlement, or attempting to start a new one.

Observation

During each timestep, the view updates to display the number and location of household and settlement agents on the model landscape. Plots track: the number of settlements; the current population; the percentage of households that are stressed; the population growth rate; the number of disbanded households; a histogram showing the population size of settlements; the average settlement population; the number of migrating household; the average distance between settlements and their nearest neighbour; and the number of individual deaths.

Monitors on the interface track the percentage of households currently experiencing shortages of each of the five different resources.

Initialisation

Prior to initialisation, the user can modify the number of household agents that will be spawned, as well as restrict where settlement agents initially spawn (within the North-East, South-East, North-West, South-West quadrants, or anywhere that is eligible). On startup, the model imports data from multiple georeferenced datasets: elevation; land-use; productivity; and mortality. The first three datasets are assigned to patch variables, with patches adjusting their colour to reflect their state of land cover. The procedure then determines the locations on the landscape which are suitable for settlement. Settlements may not spawn on patches within a 3 km (30 patch) buffer zone around the edge of the map to prevent resources from being unreasonably restricted, and can only spawn above a certain elevation threshold. The user is also able to restrict settlements to only spawn on forested land, or on land with fertile sediment [27].

Following this, the procedure spawns ten settlements at random eligible locations. Settlements cannot spawn closer than a user-defined distance between one another. Each settlement defines suitable patches within range for cultivation and extraction (farming, foraging) by its member households. Fishing (part of foraging) may only take place on land below <-0.5875 in elevation. Foraging more generally may only take place on patches that are closer to the settlement than to any other, as tracked by the settlement-owner variable.

Finally, each settlement spawns a user-influenced number of households, which are attached to it via a community tie link. Each household is instantiated with two adult inhabitants. The adult inhabitant (i) of a household (h) is represented by the timestep during which it was produced (t_{ih}). This is calculated as the sum of a minimum age (A_m) and a value taken from a range (A_r). The latter varies based upon a uniform distribution. The product of this calculation is multiplied by -1:

$$t_{ih} = -1 * A_m + u(A_r)$$

Equation 1

For example, assuming that A_m = 16 and A_r = 9, the inhabitant is assigned (16 + 9)*-1 = -25. It is therefore 25 years old at timestep 0.

Once these adult inhabitants have been instantiated, each household then generates a list of years in which it was possible to produce a child inhabitant (opportunities; o_{ch}):

$$o_{ch} = \{\max(t_{ih}), max(t_{ih}) + 1, max(t_{ih}) + 2, \dots , -A_m - 1, -A_m\}$$

Equation 2

Each opportunity (o_{cht}) in this list has a 20% chance to produce a child (Prob(Success))):

$$Prob(success) = u(0, 1.00) \leq 0.2$$

Equation 3

For our experiments, A_m is set to 16, following numerous examples in contemporary Amazonian indigenous groups, as well as other globally [17,26]. A_r is set to 34 to reflect the prehistoric average of menopausal onset [28,29].

Details

Increase-Population

The increase-population procedure manages the formation of new households, as well as the generation of new inhabitants within them. Its first function is increasing the population capacity of settlement agents. Over time, the household members of each settlement are expected to contribute to the growth of the mound, increasing the number of people that it can accommodate. The current capacity of a mound settlement (cap_c) is dependent on two user-defined parameters: The number of timesteps taken for its capacity to grow by 10% (t_g), and the base capacity of the settlement prior to growth (cap_b). To enable growth, the model calculates a capacity modifier for each settlement at timestep *t* (cap_{modt}), based on its current population (p_{st}):

$$cap_{modt} = cap_{modt-1} + \left({}^{p_{st}}/_{cap_b}\right)$$

Equation 4

This modifier is then used to calculate the settlement's current capacity:

$$cap_{ct} = cap_b + \left({}^{cap_b}/_{10} * {}^{cap_{modt}}/_{t_g}\right)$$

Equation 5

Following this, the procedure handles the creation of new household agents. Households with children aged 16 or older are eligible for marriage and the formation of new households. The procedure identifies all eligible households within the same settlement and pairs them. Each pair then forms a new household, with the children being removed from their respective parent households and becoming part of the adult pair in the newly established one. Households cannot be paired with themselves, and for simplicity, only one child per household can marry and form a new household per timestep. If a pair cannot be made, the household with an eligible child will wait until another becomes eligible. It is also possible for a household to contain only children if both parents have died. When this occurs, the children are assumed to be raised by other members of the community. If a household becomes empty after a child marries, the household is disbanded.

Increase-population also manages the production of new children. The production of children is only possible with households that possess two living adults, and the youngest must be below 50 years of age (we assume this to be the female for the sake of parsimony). The probability of successfully conceiving a child (Prob(Success)) is modified by the user defined value (b_h):

$$Prob(Success) = u(0, 1.00) \leq b_h$$

Equation 6

Migration

The migration procedure manages migration events and the creation of new settlements. The procedure begins by determining whether each settlement experiences a migration event. This is only possible if the settlement's population exceeds a user-defined proportion of its current capacity (P_{min}). For an eligible settlement, the probability of a migration event (Prob(Mi) occurring depends upon a user defined rate (Mi_u):

$$Prob(Success) = u(0, 1.00) \leq min\left(Mi_u * \left({}^{P_{st}}/_{cap_{ct}}\right), (2 * Mi_u)\right)$$

Equation 7

If this roll is successful, a second stochastic process determines whether the migrating households within a settlement initially pursue joining another existing settlement, or attempt to create a new one instead. This is determined by a user-defined parameter (Mi_{pnew}):

$$Prob(New) = u(0, 1.00) \leq Mi_{pnew}$$

Equation 8

$$Prob(Old) = u(0, 1.00) \geq Mi_{pnew}$$

Equation 9

Households can only join another existing settlement if there is one within range that has a population below its current capacity. They can only create a new settlement if the current number of settlements within a user-defined radius (migration-distance) is below a threshold value (max-settlement-capacity). If these criteria are not met, the settlement will attempt to pursue the alternative migration strategy. If the criteria of neither strategy are met, no migration will occur.

Provided that the criteria are met, the procedure will then determine the number of migrating households (n_{mh}). This is based upon an exponential distribution with a user defined mean (e_u). For our experiments, this is set to 10% of the total number of households. No fewer than five households can migrate during an event (anything smaller automatically fails), and it also cannot exceed 50% of the total:

$$n_{mh} = \min(e(0.1 * e_u), 0.5 * e_u)$$

Equation 10

Following this, a habitability score (H_{sc}) is calculated for each candidate settlement for the households wishing to join an existing one. When creating a new settlement, households will instead calculate a suitability (S_{sc}) score for up to 100 eligible candidate patches that could support one. Prior to calculating suitability scores, households will also try to reactivate any abandoned settlements within range.

Habitability is calculated using the following formula:

$$H_{sc} = Pd_{sc} - Cr_{sc} + Pop_{sc} - D_{sc} + Di_{sc}$$

Equation 11

Individual component scores are calculated for: the positive (Pop_{sc}) and negative (Cr_{sc}) effects of population; the distance to a particular candidate, both positive (D_{sc}) and negative (Di_{sc}); and whether or not the land is productive (Pd_{sc}). Each factor can be weighted, meaning the user is able to select which factors weigh heavily, lightly, or are entirely factored out of agent decision making. When weighted equally, each component provides the same value to overall utility.

Settlements are considered more productive if they lie atop the fertile sediment lobe described by Lombardo *et al.* [27]. Patches within this area have a productivity (P) of 1. Those outside have a productivity of 0. A component is calculated based on this value:

$$Pd_{sc} = Pd_w * (100 * Pd)$$

Equation 12

The distance component (D_{sc}) decreases as its distance (D) increases from the settlement to which the enquiring household is attached. This applies relative to the radius (R_m) within which households are able to migrate, with no value added when patches are at the outer limit of the radius The loss of value increases non-linearly:

$$D_{sc} = D_W * 100 * \frac{D^2}{{R_m}^2}$$

Equation 13

In contrast, the dispersal component (Di_{sc}) reflects the direct antithesis, gaining value at greater distances:

$$Di_{sc} = Di_W * 100 * \frac{D^2}{{R_m}^2}$$

Equation 14

The population component (Pop_{sc}) increases value where settlements have higher population relative to their capacity. It is also non-linear:

$$Pop_{sc} = Pop_W * 100 * \frac{{p_{st}}^2}{{cap_{ct}}^2}$$

Equation 15

In contrast, the overcrowding component (Cr_{sc}) decreases value where populations are higher:

$$Cr_{sc} = Cr_W * 100 * \frac{{p_{st}}^2}{{cap_{ct}}^2}$$

Equation 16

With regards to the suitability of patches to starting new settlements, three components are included:

$$S_{sc} = Pd_{sc} + D_{sc} + Di_{sc}$$

Equation 17

Suitability scores function in the same way as those described in equations 12-14. However, there are additional restrictions. Similar to during initialisation, patches cannot be settled if they classed as "Water" or have an elevation value below the lowest mound on the landscape. They are also ineligible if they exist within the buffer zone. The user can further restrict the model so patches can only be settled if they are forested, or possess fertile sediments.

Once a suitable site is selected, households detach from their prior settlement and abandon their territory. If joining an existing settlement, they will immediately attach to it. If starting a new settlement, the selected patch first produces a settlement agent, with the settlement-owner patch variable updating to account for it. Following this, the migrating households will attach themselves to it. Any reoccupied settlement is reactivated in a similar way, updating local territory to reflect that the settlement is no longer abandoned.

Convert-Land

In the convert-land procedure, households claim and convert patches on the virtual landscape to satisfy their resource demands. The procedure begins with defensive programming to prevent agents from trying to manage agroforestry when only savanna patches are available for cultivation. Following this, the procedure updates household demand values to reflect changes in the number of adults and children following the previous timestep. This occurs for all five resources: Maize (M), Tree Crops (F), Fuelwood (W), Palm leaves (Pa) and Animal Protein (Pr):

$$M_d = (M_{da} * n_{ah}) + (M_{dc} * n_{ch})$$

Equation 18

$$F_d = (F_{da} * n_{ah}) + (F_{dc} * n_{ch})$$

Equation 19

$$W_d = (W_{da} * n_{ah}) + (W_{dc} * n_{ch})$$

Equation 20

$$Pa_d = (Pa_{da} * n_{ah}) + (Pa_{dc} * n_{ch})$$

Equation 21

$$Pr_d = Pr_{mod} * (Pr_{da} * n_{ah}) + (Pr_{dc} * n_{ch})$$

Equation 22

In each case above, demand is calculated by multiplying amount of resource used by each adult (a) and child (c) with the number of adults (n_{ah}) and children (n_{ch}) within the household.

Households use these values to determine whether they need to claim additional patches of land to satisfy their resource demands. This is done by assessing whether they expect their existing territory to produce a sufficient quantity of each resource. An additional user-defined parameter (Pr_{mod}) modifies the amount of protein demanded based on the proportion of dietary protein assumed to be sourced from animals.

$$M_{es} \; = \; 0.85 * n_{pct} * \mathrm{M}_c$$

Equation 23

The above calculation accounts for both the number of patches within their territory (n_{pct}) and the mean productivity of maize per hectare of suitable land (M_c), a constant determined by a slider on the user interface. We assume that households are accurately able to determine the average maize yield per hectare of land within the MMR, as well as anticipating a 15% loss in yield to pests (Ringhofer 2010:99). However, this knowledge is imperfect, and they cannot account for random interannual variability (see resource-production).

M_{es} is subsequently compared to the amount of maize demanded by the household (M_d) to determine whether the household suffers from regular maize shortages. As each household is assumed to comprise of two adults and three children, this demand value is fixed and determined by a constant within the model.

$$Shortage = M_d * O - M_{es} > \; 0$$

Equation 24

$$Severe\ Shortage = M_d * O - (\, M_{es} + M_c) > \; 0$$

Equation 25

An expected maize shortage occurs where expected maize supply is unable to account for demand. Expected supplies are considered severely short if this disparity is larger than the supply of Maize that an additional hectare of cropland could provide. Please also note that households attempt to acquire more than their base demand to account for potential shortfalls. This is encoded as an overproduction modifier (O), selected by the user on a slider on the interface.

If households are set to intentionally cultivate the surrounding forest (determined by the intentional-agroforestry slider), they will assess whether their territory is expected to produce sufficient quantities of Tree crops (F_{es}), Fuelwood (W_{es}) and Palm Leaves (Pa_{es}):

$$F_{es} \; = \; N_{pf} * \mathrm{F}_c$$

Equation 26

$$W_{es} \; = \; N_{pf} * \mathrm{W}_c$$

Equation 27

$$Pa_{es} \; = \; N_{pf} * \mathrm{Pa}_c$$

Equation 28

These calculations function similarly to maize, though are instead calculated based on land that is able to become forested (N_{pf}). This includes both active agroforestry and cropland that will

subsequently become agroforestry after it is abandoned, preventing agents from repeatedly cultivating land until their territory begins to produce forest resources. Instead, they are assumed to forage for resources on unclaimed land until their plots become productive. When intentional-agroforestry is enabled, it is impossible for agents to own fallowed land that can become forested, though it is possible for them to own fallows in the open savanna. As with maize, households cannot account for natural variations in the productivity of these resources, but can accurately estimate their average yield per hectare. Agents do not expect to produce sufficient animal protein from their territory; it must be procured over a larger area.

Households expecting a resource shortage are separated into three categories: those severely short of maize, those short of maize and no other resource, and those with a shortage in at least one forest product. These categories are hierarchical and mutually exclusive. A severe maize shortage is prioritised over all other shortages. If a household has a shortage of forest products, it will be designated as short of forest resources even if it also lacks maize. Each category undergoes a different process of land conversion.

Following this, household agents determine whether to resolve their resource shortages either by reactivating fallowed land within their existing territory, or by choosing to claim new land. This is only possible when converting land to produce maize, as land agroforestry cannot immediately be reactivated.

The choice to reactivate fallowed land is modelled stochastically, with the chance based upon the probability-of-reactivation slider on the user interface. Households can only reactivate land that was fallowed no less than a user-defined number of timesteps ago, selected via the fallow-period slider. Land cannot be reactivated if it more than 15 years old, mirroring the reestablishment of woody plants (Finegan 1984; Peña-Claros 2003; Ringhofer 2010). If multiple patches are reactivated, they must be selected from the open savanna, mirroring the increased difficulty of clearing forested land. If no land can be reactivated, the choice is skipped. If the reactivated land is sufficient to satisfy household demand, no further patches will be claimed.

If no fallowed land is available, or the household has decided against reactivation, they will instead select (a) new patch(es) to claim and cultivate. To do this, households will assess a subset of the patches within a set area of their settlement mound. Both the number of patches and the radius of eligibility are user-defined, chosen via the percent-patches-considered and agricultural-radius sliders respectively. The number of patches considered is proportional to the size of this radius. Not all patches within range are eligible; those with a land use category of 'water', or those already owned and cultivated by other households are automatically excluded. In addition, the user can further restrict households to only cultivate patches of open savanna, or alternatively only those that can become forested.

MoundSim LandUse offers three different methods for agents to select patches. A null model ("NULL") is available, which forces households to randomly choose one patch from the subset. The second, "CANAL-CAUSEWAY", is an iterative procedure that selects the patch closest to known earthworks on the landscape. This uses imported vector data denoting the locations of canals and causeways associated with the Casarabe culture. In the third model, "VARIABLES", households select patches by calculating a utility score (U_{sc}) for each potential site based on multiple characteristics:

$$U_{sc} = Pd_{sc} + E_{sc} + Fl_{sc} - D_{sc} - Df_{sc} + A_{sc}$$

Equation 29

Patches calculate individual component scores for: Productivity (P_{sc}); Elevation (E_{sc}); Flooding (Fl_{sc}); Distance (D_{sc}); Clearance Difficulty (Df_{sc}); and Aggregation (A_{sc}). Each variable is calculated independently and can be weighted, meaning the user is able to select whether each factor weighs heavily, lightly, or is entirely factored out of decision making. When weighted equally, each component provides the same value to overall utility.

Patches are considered more productive if they lie within the fertile sediment lobe described by Lombardo *et al.* [27]. Patches within this area have a productivity (P) of 1. Those outside have a productivity of 0. A score is calculated based on this value:

$$P_{sc} = P_w * (100 * P)$$

Equation 30

The final value is multiplied by the weighting for this criterion (P_w), selected by the user on the interface.

Elevation (E_{sc}) is the preference for households to select patches in areas less likely to flood regularly, dependent on the patches elevation variable (E). Scores vary between nothing below -2 elevation and maximum points above 2.04063. This reflects the elevation of the lowest mound on the contemporary landscape, a high point at which flooding does not occur and thus no additional benefit can be derived from selecting patches at higher elevation.

$$E_{sc} = E_w * \max(0, \min(100, (100 * (E + 2) / 4.04))) \qquad (9)$$

Flooding (Fl_{sc}) is the preference for households to select patches in areas more likely to flood regularly. At present, it is unknown when members of the Casarabe culture may have cultivated maize. It is possible for this to have occurred during the dry season, when much of the savanna was available. At such a time, access to water may have been crucial. This calculation acts as a reverse of the above slider, with no value derived from land with an elevation value of +2.04063 or more, and no additional benefit gained from land below -2. On this landscape, extremely low-lying areas are considered swamps and thus unsuitable for agriculture:

$$Fl_{sc} = Fl_w * 100 - \max(0, \min(100, (100 * (E + 2) / 4.04))) \qquad (10)$$

The distance score (D_{sc}) of a patch decreases as its distance increases from the settlement to which the household is attached. This applies relative to the radius (R_a) within which households are able to cultivate, with no value added when patches are at the outer limit of the radius The loss of value increases non-linearly:

$$D_{sc} = D_w * 100 * (D^2 / R_a^2) \quad (11)$$

The difficulty component (Df_{sc}) decreases as its vegetation score (V) increases. At low levels, the patch is considered easy to clear and thus of increased value:

$$Df_{sc} = Df_w * 100 * (V / V_{Fmax}) \quad (12)$$

This value applies relative to the maximum vegetation score of forested land, as defined by the user (V_{Fmax}). The final component, Aggregation (A_{sc}), increases patch suitability if the patch borders another currently either: i) being actively managed as cropland or agroforestry or ii) is currently fallowed. As with the other mechanics above, it can be weighted (A_w).

$$A_{sc} = A_w * (\text{Border} \rightarrow 100, \neg\text{Border} \rightarrow 0) \quad (13)$$

Under certain circumstances, a household may not be able to find a patch of suitable land. For example, they may fail to find enough space as all patches within range have been claimed. If such a scenario arises, households will instead attempt to reactivate a patch of fallowed land they have access to, or instead sacrifice plots of agroforestry to produce sufficient quantities of maize (as the staple crop, maize is considered more important).

Once a site is selected, it is converted into cropland. Fallowed territory is reactivated by transitioning to the “Cropland” land-use category, resetting the last-converted value to the current tick count, and reducing the patch vegetation score to 0 (to reflect the vegetation being cleared). Resource supply scores are also reset to prevent the patch from supplying incorrect values. When creating new cropland, the land-use, last-converted, vegetation, and resource supply scores are set in an equivalent way. In addition, the patch is added to the household’s territory (stored in the household-territory household variable), and has its owner variable set to the households ID number. Transition to agroforestry and fallowed land is managed within the vegetation-growth sub-model.

Resource-Production

In the resource-production procedure, resources are assigned to patches owned by household agents on the virtual landscape. Firstly, the communal resource pools of settlement agents are reset to prevent interannual storage. Resources are then assigned to patches according to their land-use type. Three types of land-use are considered: Cropland, Fallow, and Agroforestry. Only patches owned by household agents receive these resources. These supplies overwrite those remaining from the previous year to prevent storage.

	Land-Use Type	
	Cropland	Forest [Agroforestry/Fallow]
Maize [kg ha^{-1}]	[Varies]	0
Forage [kg ha^{-1}]	0	760
Fuelwood [kg ha^{-1}]	0	1200
Palm [leaves ha^{-1}]	0	206
Protein [kg ha^{-1}]	0	0.23

Table 1: Resources assigned to each patch in the resource-production procedure

Cropland patches are assumed to exclusively produce maize, and we assume no decrease in yield takes place throughout the cultivation period for the sake of simplicity. The productivity of maize per hectare (M_S) is determined by a slider on the user interface (cropland-production; M_c). In contrast, agroforestry, and forested fallow land produces forest resources (F_s, W_s, Pa_s, Pr_s), which are defined by constants (F_c, W_c, Pa_c, Pr_c). These values can also be multiplied by the user-defined forest production modifier (Fpi), assumed to represent forest enrichment due to human intervention. It is also modulated based on the patch vegetation score (V), discussed more under the 'vegetation-growth' procedure. For all resources, the quantity produced varies upon a normal distribution with a standard deviation of 15% of the mean value:

$$M_s \sim N(\mathrm{M}_c, 0.15 * \mathrm{M}_c)$$

Equation 31

$$F_s \sim \mathrm{Fpi} * \mathrm{V} * N(\mathrm{F}_c, 0.15 * \mathrm{F}_c)$$

Equation 32

$$W_s \sim \mathrm{Fpi} * \mathrm{V} * N(\mathrm{W}_c, 0.15 * \mathrm{W}_c)$$

Equation 33

$$Pa_s \sim \mathrm{Fpi} * \mathrm{V} * N(\mathrm{Pa}_c, 0.15 * \mathrm{Pa}_c)$$

Equation 34

$$Pr_s \sim \mathrm{Fpi} * \mathrm{V} * N(\mathrm{Pr}_c, 0.15 * \mathrm{Pr}_c)$$

Equation 35

Decrease-Population

This procedure represents the processes associated with the dissolution of households, as well as the death of individuals within them. There are two primary mechanisms to the procedure: i) age and ii) insufficient resources.

Age-related mortality is conducted at the inhabitant level, utilising age-specific mortality data imported into the model during initialisation [32]. The process is modelled stochastically, where the probability of death (Prob(Death)) is calculated as:

$$Prob(Death) \; = \; u(0, 1.00) \; <= \; Mo_{iha}$$

Equation 36

Mo_{iha} reflects the probability of mortality for individual *i* from household *h* at age *a.* Any individual that dies as a result of this process has its birth age removed from the respective adult or child variable of the household they inhabited.

Following this, the model manages mortality associated with resource shortages. The procedure begins by altering Household expectations to account for the mean productivity of each resource from their territory in the current timestep:

$$M_{ct} \; = \; (M_{ct-1} + mean(M_{st})/2)$$

Equation 37

$$F_{ct} \; = \; (F_{ct-1} + mean(F_{st})/2)$$

Equation 38

$$W_{ct} \; = \; (W_{ct-1} + mean(W_{st})/2)$$

Equation 39

$$Pa_{ct} \; = \; (Pa_{ct-1} + mean(Pa_{st})/2)$$

Equation 40

$$Pr_{ct} \; = \; (Pr_{ct-1} + mean(Pr_{st})/2)$$

Equation 41

In each case, expected productivity from the previous timestep is averaged with mean productivity from the current one.

The procedure then determines whether household agents have produced sufficient quantities of each resource. Each household first calculates whether sufficient quantities of Maize, Tree Crops, Fuelwood, Palm Leaves, and Protein have been obtained from its territory:

$$M_{ts} = 0.85 * \sum_{i=1}^{n_{pt}} M_{si}$$

Equation 42

Here, n_{pt} refers to the number of patches within the Household's territory. This is similarly calculated for forest resources:

$$F_{ts} = \sum_{i=1}^{n_{pt}} F_{si}$$

Equation 43

$$W_{ts} = \sum_{i=1}^{n_{pt}} W_{si}$$

Equation 44

$$Pa_{ts} = \sum_{i=1}^{n_{pt}} Pa_{si}$$

Equation 45

$$Pr_{ts} = \sum_{i=1}^{n_{pt}} Pr_{si}$$

Equation 46

Each household then calculates whether its demands have been satiated by the supplies calculated above, or whether residual demand remains (M_r, F_r, W_r, Pa_r, Pr_r):

$$M_r \ = \ M_d - (0.85 * M_{ts})$$

Equation 47

$$F_r \ = \ F_d - F_{ts}$$

Equation 48

$$W_r \ = \ W_d - W_{ts}$$

Equation 49

$$Pa_r = \ Pa_d - Pa_{ts}$$

Equation 50

$$Pr_r \ = \ Pr_d - Pr_{ts}$$

Equation 51

As described above, Maize supplies are further multiplied by 0.85 to account for unintended crop loss (Ringhofer 2010:99).

If a household suffers from a resource shortfall, it may still survive by obtaining resources from i) communal stores owned by its settlement and ii) unclaimed patches of land on the landscape. The

former is only possible if the communal pooling of resources is enabled via the communal-resource-pools? switch. Households may only obtain Maize, Fuelwood, and Animal protein in this way. Palm leaves are typically collected on demand, and Foraged tree crops are supplementary to the diet and cannot be stockpiled. In comparison, it is common for maize and fuelwood to be stockpiled for consumption intra-annually, and animal protein is commonly shared in contemporary Amazonian communities (In some cases the lack of sharing can lead to disunity e.g., Good (1987)). These resources are shared freely under the assumption of generalised reciprocity (donor households would expect to receive similar aid in a similar situation). Resources cannot be shared between settlements.

Households may also obtain resources from unclaimed patches of forested land controlled by their settlement. Settlements may directly control patches of territory up to 7km (within daily walking distance (Beckerman 1987; Binford 2001)). However, for it to be controlled, it must be nearer to that settlement than to any other. Only settlements that possess household members may control territory:

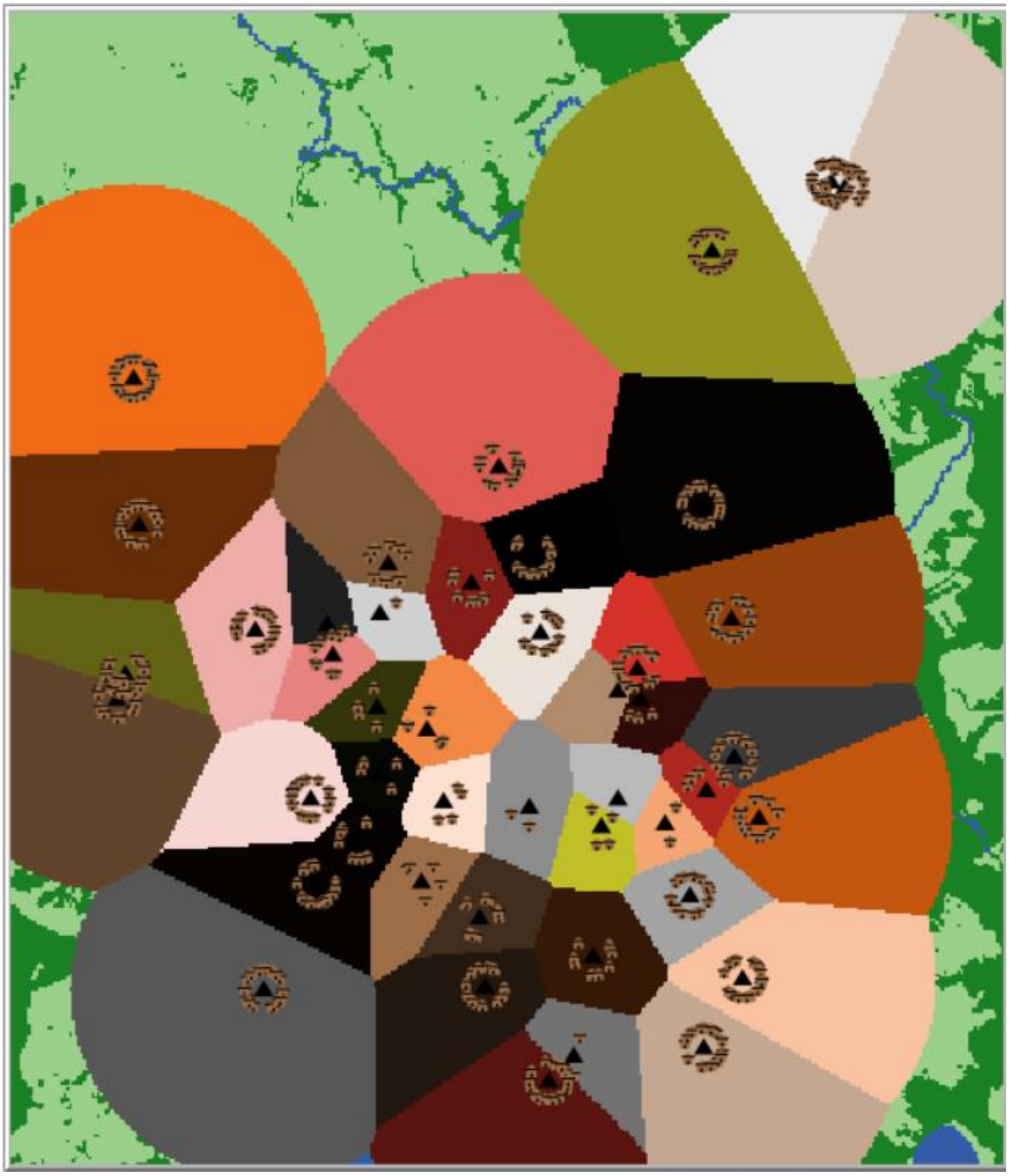

Figure 1: Patches controlled by settlements within the North-East Quadrant of MoundSim Population. This initialised upon startup and tracked using the settlement-owner patch variable.

Unclaimed patches of forest (patches with a land-use value of either “Old Growth Forest” or “New Growth Forest”) that meet these criteria can provide additional Foraged Tree Crops, Fuelwood, Palm Leaves and Animal protein to the households of that settlement. This is managed through the settlement agents. First, each settlement produces a value reflected the quantity of each resource produced per hectare by unclaimed patches it controls. Second, it calculates the total number of old (n_{OldFor}) and new (n_{NewFor}) growth forest patches it controls. From these two values, the calculates the total quantity of each resource that can potentially be extracted from unclaimed forest patches it controls (F_u, W_u, Pa_u, Pr_u):

$$F_u = (Fpi * F_s * n_{NewFor}) + (F_s * n_{OldFor})$$

Equation 52

$$W_u = (Fpi * W_s * n_{NewFor}) + (W_s * n_{OldFor})$$

Equation 53

$$Pa_u = (Fpi * Pa_s * n_{NewFor}) + (Pa_s * n_{OldFor})$$

Equation 54

$$Pr_u = Pr_s * (n_{NewFor} + n_{OldFor})$$

Equation 55

In addition, each settlement is able to extract additional animal protein from fish produced at low elevations in the open savannas:

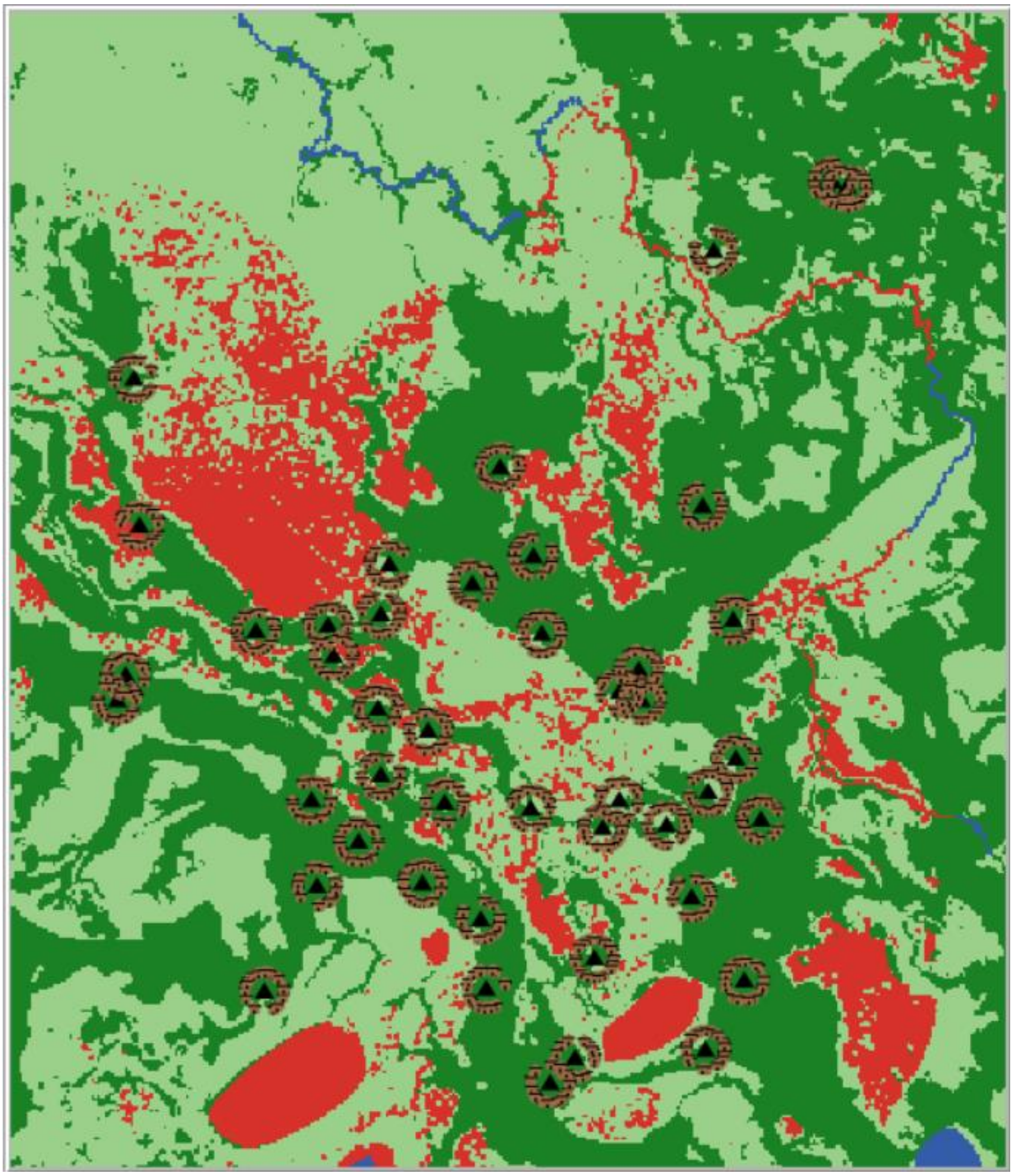

Figure 2: Location of Fish producing patches within the North-East Quadrant of MoundSim Population. To be considered viable, patches must possess an elevation value below -0.5875. They must also be controlled by that settlement (managed by the settlement-owner patch variable).

Aquatic protein is only accounted for if the fishing-modifier slider is set to above 0.0 on the user interface. The total quantity of unclaimed fishing protein available for extraction depends upon the number of patches that can produce fish within territory controlled by the settlement (n_{Fi}), as well as a user-defined density of fish available within a hectare of eligible land (Den_{Fi}):

$$Fi_u = n_{Fi} * 0.084 * \mathrm{Den}_{Fi}$$

Equation 56

Households experiencing a shortage in any of these resources can then obtain unclaimed resources from these areas.

Finally, the procedure identifies household agents that were unable to acquire sufficient quantities of each resources from any of the above methods. The model allows the user to determine whether agents die as a result of these shortfalls. This is determined by the *-die? Switches on the user interface. If a household lacks sufficient quantities of a resource with the respective *-die? switch turned on, that household will abandon its territory and die. However, if the switch is turned off, the household will instead be marked as under resource stress (with the respective *-stressed? variable switched to "True") until the next time decrease-population is executed. This allows the user to track the number of individuals stressed as the result of a particular resource shortage. The number of dissolved households is also tracked separately by resource for this purpose.

When household territory is abandoned, the patch's owner variable is reset. If the territory is actively being farmed as cropland, the land-use variable will be set to "Fallow".

Vegetation-Growth

The vegetation-growth procedure represents the continuous growth of vegetation over time. For the sake of parsimony, we solely focus on secondary succession taking place on land abandoned by household agents, tracked using a boolean variable. The first task managed by this procedure is succession on abandoned land. In the LM, local edaphic and hydrological conditions cause succession to vary between forests on high ground and low-lying savanna (Mayle et al. 2007). This begins after land is first cleared, but is stalled until the end of the cropping cycle by humans through regular weeding (e.g., Beckerman 1987; Ringhofer 2010; Staver 1989), proceeding unhindered thereafter. In MoundSim LandUse, we define the point when land is considered to be regenerated the point at which local Indigenous communities can no longer differentiate between fallow and secondary forest (Huanca 1999:59). The model enables the user to vary this parameter, which is defined by sliders on the user interface.

Within the model, we account for the process of succession using the vegetation patch variable. This is calculated as a normally distributed value with a mean and standard deviation dependent on the current timestep (t) and the timestep during which the patch was last clear (t_{lc}). These two variables can be used to calculate the number of years of secondary succession that has taken place unhindered. This is converted to a vegetation score by transforming it based on the natural logarithm:

$$V \sim N(\log_e(t - t_{lc}), \log_e(0.0025 * \log_e(t - t_{lc}))) \quad (35)$$

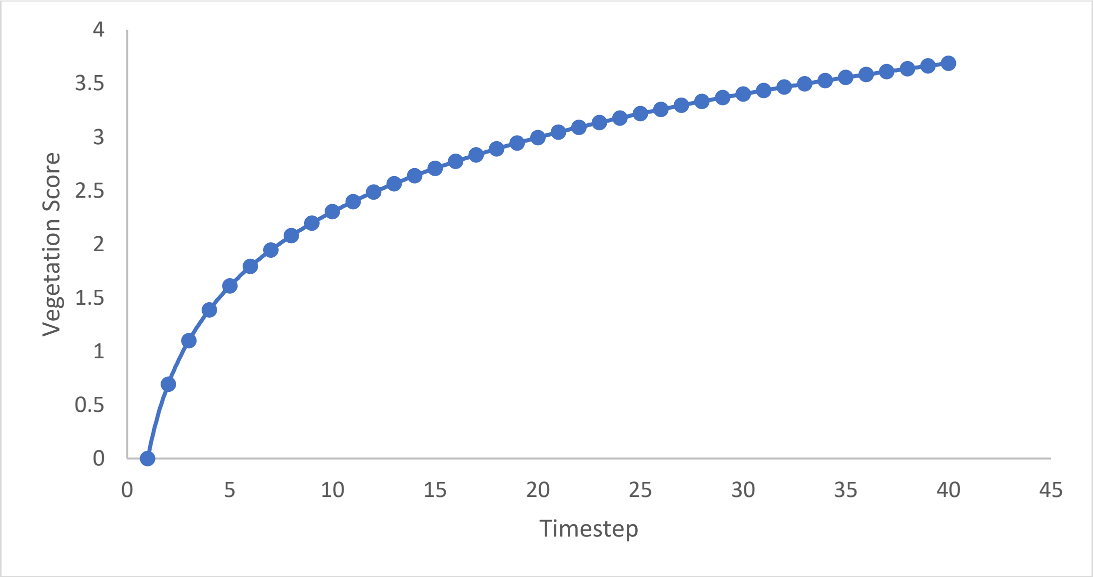

Figure 3: mean vegetation score for patches during timesteps subsequent to abandonment.

Once a patch's vegetation score exceeds a threshold, it will transition to New Growth Savanna or New Growth Forest depending on the relevant succession pathway. This threshold depends on which pathway the patch takes. This value can vary between 10 and 120 years within the model following the results of Poorter *et al.* [37]. There is a lack of information regarding the rate of savanna regeneration in the LM, so we assume a 5-50 year transition rate to account for the faster establishment of herbaceous vegetation. The model follows the Tsimane system of agriculture in that households retain land rights after the land is fallowed (Piland 1991), which is subsequently lost after it becomes indistinguishable from secondary forest/savanna.

The second task managed is the abandonment of unproductive cropland. Contemporary cropping systems typically produce crops for three years before fallowing (e.g., Beckerman 1987; Denevan 2001; Piland 1991; Staver 1989). In MoundSim LandUse, the user is able to determine whether the land is abandoned between one and seven years after first cultivation. The procedure also ensures that households only manage up to six patches of land within their territory at any one time (Beckerman 1987; Piland 1991). In such cases, the oldest patch is abandoned. Finally, the procedure ensures that any patches abandoned by their owner become fallowed and are entered into the succession system.

References

[1] Grimm V, Berger U, DeAngelis D, Polhill G, Giske J, Railsback S. The ODD protocol: A review and first update. Ecol Modell 2010;221:2760–8.

[2] Grimm V, Berger U, Bastiansen F, Eliassen S, Ginot V, Giske J, et al. A standard protocol for describing individual-based and agent-based models. Ecol Modell 2006;198:115–26. https://doi.org/10.1016/j.ecolmodel.2006.04.023.

[3] Müller B, Bohn F, Dreßler G, Groeneveld J, Klassert C, Martin R, et al. Describing human decisions in agent-based models - ODD+D, an extension of the ODD protocol. Environmental Modelling and Software 2013;48:37–48. https://doi.org/10.1016/j.envsoft.2013.06.003.

[4] Steward J. Handbook of South American Indians Volume 5: The Comparative Ethnology of South American Indians. Washington D.C.: United States Government Printing Office; 1949.

[5] Denevan W. The Native Population of the Americas in 1492. 1st ed. Madison: University of Wisconsin Press; 1976.

[6] Denevan W. The Native Population of the Americas in 1492. 2nd ed. Madison: University of Wisconsin Press; 1992.

[7] Lombardo U. Estimating the pre-Columbian population of southwester Amazonia. The 81st Annual Meeting of the Society for American Archaeology, Vancouver: 2017, p. tDAR id: 429243.

[8] Mann C. Earthmovers of the Amazon. Science (1979) 2000;287:786–9.

[9] Denevan W. Estimating Amazonian Indian Numbers in 1492. Journal of Latin American Geography 2014;13:207–21. https://doi.org/10.1353/lag.2014.0036.

[10] Railsback S, Grimm V. Agent-Based and Individual-Based Modeling: A practical Introduction. Woodstock: Princeton University Press; 2012.

[11] Railsback S, Grimm V. Agent-Based and Individual-Based Modeling: A Practical Introduction. 2nd ed. Oxford: Princeton University Press; 2019.

[12] Wilensky U. NetLogo. Center for Connected Learning and Computer-Based Modeling, Northwestern University, Evanston, IL 1999. http://ccl.northwestern.edu/netlogo/.

[13] Lombardo U, Prümers H. Pre-Columbian human occupation patterns in the eastern plains of the Llanos de Moxos, Bolivian Amazonia. J Archaeol Sci 2010;37:1875–85. https://doi.org/10.1016/j.jas.2010.02.011.

[14] Jaimes Betancourt C. La cerámica de la Loma Salvatierra, Beni-Bolivia. La Paz: Plural Editores.; 2012.

[15] Jaimes Betancourt C. La cerámica de la Loma Mendoza. In: Prümers H, editor. Loma Mendoza. Las Excavaciones de los años 1999-2002, La Paz: Plural Editores; 2015, p. 89–222.

[16] Prümers H. Loma Mendoza. Las Excavaciones de los años 1999-2002. La Paz: Plural Editores; 2015.

[17] Binford L. Constructing Frames of Reference: An Analytical Method for Archaeological Theory Building Using Ethnographic and Environmental Data Sets. 1st ed. London: University of California Press; 2001.

[18] Lake M. Trends in Archaeological Simulation. J Archaeol Method Theory 2014;21:258–87. https://doi.org/10.1007/s10816-013-9188-1.

[19] Malthus T. An Essay on the Principle of Population. 1st ed. London: Pickering & Chatto Publishers; 1798.

[20] Arthur B. Inductive Reasoning and Bounded Rationality. Am Econ Rev 1994;84:406–11.

[21] Dickau R, Bruno M, Iriarte J, Prümers H, Jaimes Betancourt C, Holst I, et al. Diversity of cultivars and other plant resources used at habitation sites in the Llanos de Mojos, Beni, Bolivia: Evidence from macrobotanical remains, starch grains, and phytoliths. J Archaeol Sci 2012;39:357–70. https://doi.org/10.1016/j.jas.2011.09.021.

[22] Bruno M. Carbonized Plant Remains from Loma Salvatierra, Department of Beni, Bolivia. Zeitschrift Für Archäologie Außereuropäischer Kulturen 2010;3:153–208.

[23] Whitney B, Dickau R, Mayle F, Soto D, Iriarte J. Pre-Columbian landscape impact and agriculture in the Monumental Mound region of the Llanos de Moxos, lowland Bolivia. Quaternary Research (United States) 2013;80:207–17. https://doi.org/10.1016/j.yqres.2013.06.005.

[24] Prümers H, Jaimes Betancourt C, Iriarte J, Robinson M, Schaich M. Lidar reveals pre-Hispanic low-density urbanism in the Bolivian Amazon. Nature 2022;606:325–8. https://doi.org/10.1038/s41586-022-04780-4.

[25] Huanca T. Tsimane Indigenous Knowledge, Swidden Fallow Management and Conservation. University of Florida, 1999.

[26] Ringhofer L. Fishing, Foraging and Farming in the Bolivian Amazon. 2010. https://doi.org/10.1007/978-90-481-3487-8.

[27] Lombardo U, May JH, Veit H. Mid- to late-Holocene fluvial activity behind pre-Columbian social complexity in the southwestern Amazon basin. Holocene 2012;22:1035–45. https://doi.org/10.1177/0959683612437872.

[28] Chamberlain A. Demography in Archaeology. Cambridge: Cambridge University Press.; 2006.

[29] Hassan F. Demographic Archaeology. New York: Academic press; 1981.

[30] Finegan B. Forest Succession. Nature 1984;312:109–14.

[31] Peña-Claros M. Changes in Forest Structure and Species Composition during Secondary Forest Succession in the Bolivian Amazon. Biotropica 2003;35:450–61. https://doi.org/10.1111/j.1744-7429.2003.tb00602.x.

[32] Gurven M, Kaplan H, Supa AZ. Mortality experience of Tsimane Amerindians of Bolivia: Regional variation and temporal trends. American Journal of Human Biology 2007;19:376–98. https://doi.org/10.1002/ajhb.20600.

[33] Good K. Limiting Factors in Amazonian Ecology. In: Harris M, Ross E, editors. Food and Evolution: Towards a Theory of Human Food Habits, Philadelphia: Temple University Press; 1987, p. 207–421.

[34] Beckerman S. Swidden in Amazonia and the Amazon Rim. In: Turner II BL, Brush Stephen, editors. Comparative Farming Systems, New York: The Guilford Press; 1987, p. 55–94.

[35] Mayle F, Langstroth R, Fisher R, Meir P. Long-term forest-savannah dynamics in the Bolivian Amazon: Implications for conservation. Philosophical Transactions of the Royal Society B: Biological Sciences 2007;362:291–307. https://doi.org/10.1098/rstb.2006.1987.

[36] Staver C. Why farmers rotate fields in maize-cassava-plantain bush fallow agriculture in the wet Peruvian Amazon. Hum Ecol 1989;17:401–26. https://doi.org/10.1007/BF00889498.

[37] Poorter L, Craven D, Jakovac C, van der Sande M, Amissah L, Bongers F, et al. Multidimensional tropical forest recovery. Science (1979) 2021;374:1370–6.

[38] Piland R. Traditional Chimane agriculture and its relation to soils of the Beni biosphere reserve. University of Florida., 1991.

[39] Denevan W. Cultivated Landscapes of Native Amazonia and the Andes. Oxford: Oxford University Press; 2001.
