# Supplementary material for: Constraining the population size estimates of the pre-Columbian Casarabe Culture of Amazonian Bolivia
Source: PLoS One. 2025 May 30;20(5):e0325104. doi: 10.1371/journal.pone.0325104 (PMC12124758; doi:10.1371/journal.pone.0325104)
Supplement: S5 File — File containing details on the spatial data imported into MoundSim Population. (PDF) [file pone.0325104.s005.pdf]

Imported Datasets

Upon initialisation, four datasets are imported into MoundSim Population. These datasets contain information on environmental characteristics (Elevation, Productivity, Land-Use) and one demographic characteristic (age-specific mortality). Where spatial data has been modified, alterations were performed in ArcGIS Pro Version 2.7.0 [1]. A configuration file is also imported to parameterise the model.

Age-specific Mortality

Age-specific mortality data was sourced from Gurven *et al.* [2]. This data was collected based upon the mortality data of 18 Tsimane villages, together constituting 31% of this indigenous population. A Siler distribution model was subsequently applied to this mortality data to account for higher mortality associated with the elderly and the very young, as well as lower mortality in young adulthood. This function was sampled at whole integers to estimate age-specific mortality. For the sake of parsimony, data was modified to ensure mortality beyond 80 years. Data is imported into the model in tabular format as a .json file. Within the model, this data is mapped to a lookup table within which agents search when calculating mortality.

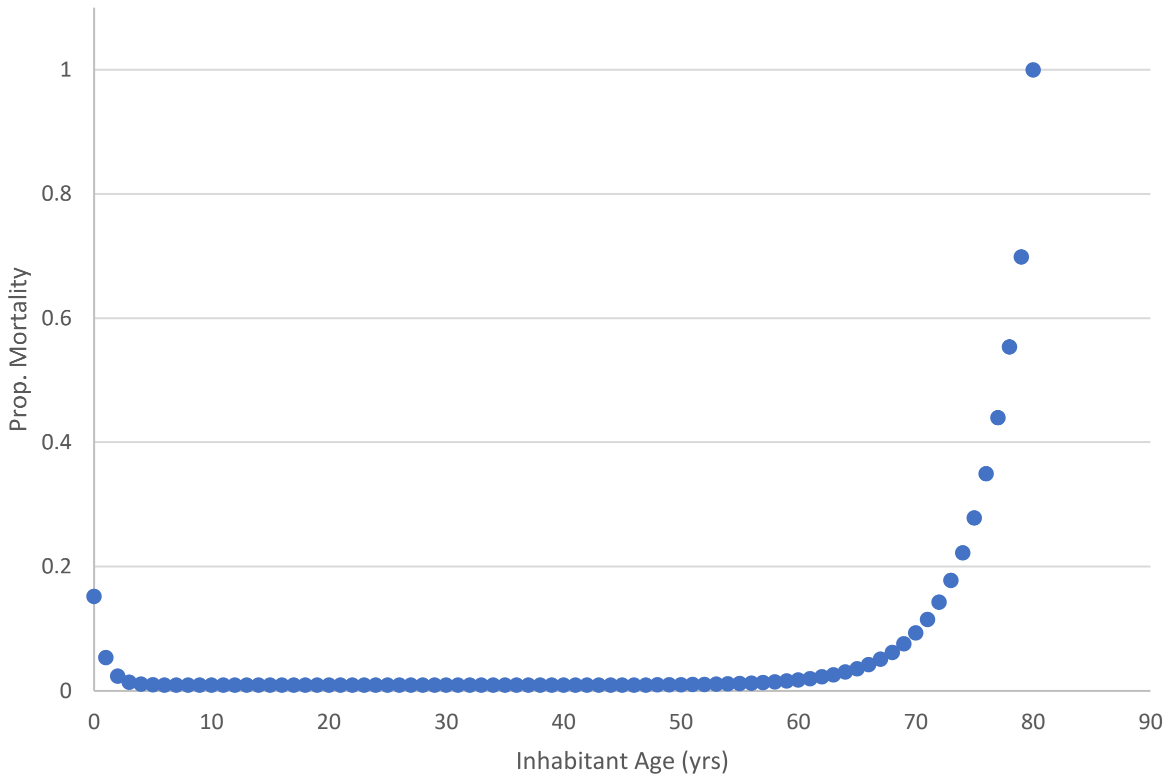

Figure 1: Siler function for mortality data in MoundSim Population.

Land-Use

A single dataset was imported to represent the initial state of land-use within the model. This dataset was developed specifically for this study using satellite data from ESRI, Google Earth, and Bing Maps. All levels considered to possess "substantial" levels of arboreal coverage based on imagery were classified as forest. A vector dataset categorising savanna and forest was developed in ArcGIS pro Version 2.7.0 [1]. This was combined with a dataset identifying water bodies within the region, which were identified using the supervised classification tool [3]. The subsequent dataset was rasterised

and resampled to 100x100 m resolution using the Resample tool. The overall estimation of arboreal coverage from this process was 39.7%, higher than the estimate originally made in Lombardo and Prümers [3]. Part of this difference may stem from differences in the resolution to which forests were mapped.

Productivity

No detailed and accurate soil maps currently exist for the MMR. As such, productivity within the model is dependent upon whether patches lie upon the sediment lobe deposited by the Rio Grande during the mid-to-late Holocene [4]. The data used to identify the location of the sediment lobe in MoundSim LandUse is combined from the article produced by Lombardo *et al.,* [4], as well as the article produced by Plotzki *et al.* [5]. This is a vectorised dataset that is mapped to a patch variable based upon whether patches intersect with polygons in the dataset.

Elevation

Elevation data in MoundSim LandUse is sourced from the TanDEM-X digital elevation model (12x12 m resolution). This data was modified to remove the shallow (0.1 mm m^{-1}) South-North gradient observed across the wider LM [4]. This procedure followed the approach outlined in Lombardo and Grützner [6], where 500 random points were placed over areas of open savanna to extract DEM values. These values were used to reconstruct a trend surface using the Trend tool, interpolated using a 12th order polynomial. Following this, elevation was detrend by subtracting this trend surface from the original DEM values. The modified dataset was subsequent interpolated to a 100*100m resolution using the Resample tool.

For all raster data above, raster files were converted to ASCI text files using the Raster to ASCI tool.

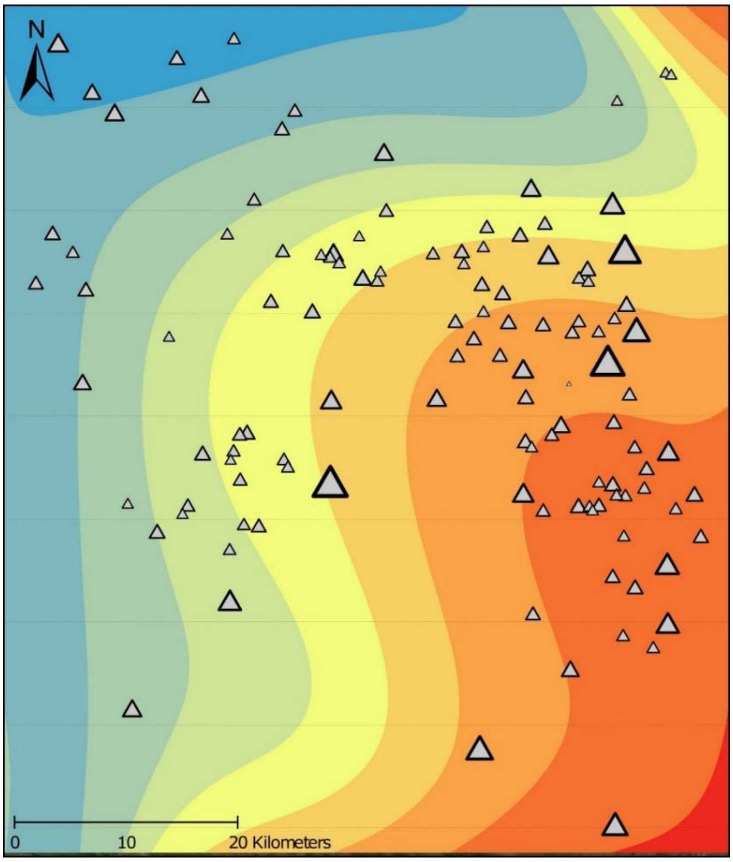

Figure 2: Trend surface used to detrend elevation data in MoundSim LandUse

Configuration File

A configuration file is also imported into the model to allow the user to assign constants prior to experimentation. Below is a list of variables managed by the configuration file:

ID	Variable	Type	Description
1	start-population-modifier	Continuous	Modifier to population produced when model is initialised
2	prob-household-birth	Continuous	Probability for household to produce child per timestep
3	yr-10%	Continuous	Timesteps taken to increase settlement base capacity by 10%
4	settlement-base-capacity	Continuous	Base population capacity for settlement prior to settlement growth
5	migration-rate	Continuous	Probability for eligible settlement to experience a migration event
6	new-settlement-probability	Continuous	Probability for migration event to initially attempt to create a new settlement
7	max-settlement-density	Continuous	Maximum number of settlements tolerated by Households within the migration distance
8	migration-distance	Continuous	Maximum distance Households will choose to migrate
9	protein-modifier	Continuous	Maximum protein percent sourced from animals (aquatic + terrestrial)
10	land-for-cultivation	Case Switch	Restricts cultivation to only areas of forest or savanna
11	mig-pop-cost	Continuous	Households preferentially migrate to less populated settlements
12	mig-dist-cost	Continuous	Households preferentially migrate to closer settlements
13	mig-pop-bonus	Continuous	Households preferentially migrate to more populated settlements
14	mig-dist-bonus	Continuous	Households preferentially migration to farther settlements
15	mig-lobe-bonus	Continuous	Households preferentially migrate to more productive land
16	intentional-agroforestry?	Boolean	Households intentionally account for forest productions when cultivating land
17	forest-restrict?	Boolean	Settlements may only form on forested land
18	lobe-restrict?	Boolean	Settlements may only form on the sediment lobe
19	maize-die?	Boolean	Maize shortages cause household inhabitants to die
20	forage-die?	Boolean	Tree crop shortages cause household inhabitants to die
21	fuelwood-die?	Boolean	Fuelwood shortages cause household inhabitants to die
22	palm-die?	Boolean	Palm leaf shortages cause household inhabitants to die
23	protein-die?	Boolean	Animal Protein shortages cause household inhabitants to die
24	start-location	Case Switch	Determines location where settlements spawn on initialisation
25	agricultural-radius	Continuous	Determines radius within which households can cultivate patches around their settlement

26	forage-radius	Continuous	Determines radius within which households can extract resources around their settlement
27	percent-patches-considered	Continuous	Percentage of patches within the agricultural radius considered by households when selecting new farm sites
28	probability-of-reactivation	Continuous	Probability for household to attempt reactivating fallow land when cultivating
29	overproduction-mod	Continuous	Amount of additional maize Households attempt to satisfy above base demand
30	forest-prod-mod	Continuous	Amount of additional resources produced by forests modified by Households
31	fallow-period	Continuous	Number of timesteps fallow land must be left fallow before it is eligible for recultivation
32	fallow-age	Continuous	Number of timesteps fallow age is cultivated before being abandoned
33	cropland-production	Continuous	Amount of maize produced per patch of cropland (kg/ha)
34	yrs-savanna-regen	Continuous	Number of timesteps for Fallow to return to New Growth Savanna
35	yrs-forest-regen	Continuous	Number of timesteps for Fallow to return to New Growth Forest
36	fish-density	Continuous	Modifier to the quantity of aquatic protein produced per eligible patch
37	excl-radius	Continuous	Radius around an active settlement within which a new settlement cannot spawn
38	model	Case Switch	Determines which preference system household agents choose to cultivate land
39	productivity-bonus	Continuous	Households preferentially select more productive patches
40	distance-cost	Continuous	Households preferentially select patches closer to their settlement
41	flooding-cost	Continuous	Households preferentially select patches at lower elevation
42	difficulty-cost	Continuous	Households preferentially select patches easier to clear
43	elevation-bonus	Continuous	Households preferentially select patches at higher elevation
44	aggregation-bonus	Continuous	Households preferentially select patches adjacent to other active/fallowed farmland

Table 1: Configuration parameters imported into MoundSim Population

References

[1] ESRI. ArcGIS Pro Desktop: Release 2.7.0 2020.

[2] Gurven M, Kaplan H, Supa AZ. Mortality experience of Tsimane Amerindians of Bolivia: Regional variation and temporal trends. American Journal of Human Biology 2007;19:376–98. https://doi.org/10.1002/ajhb.20600.

[3] Lombardo U, Prümers H. Pre-Columbian human occupation patterns in the eastern plains of the Llanos de Moxos, Bolivian Amazonia. J Archaeol Sci 2010;37:1875–85. https://doi.org/10.1016/j.jas.2010.02.011.

[4] Lombardo U, May JH, Veit H. Mid- to late-Holocene fluvial activity behind pre-Columbian social complexity in the southwestern Amazon basin. Holocene 2012;22:1035–45. https://doi.org/10.1177/0959683612437872.

[5] Plotzki A, May J-H, Preusser F, Roesti B, Denier S, Lombardo U, et al. Geomorphology and evolution of the late Pleistocene to Holocene fluvial system in the south-eastern Llanos de Moxos, Bolivian Amazon. Catena (Amst) 2015;127:102–15. https://doi.org/10.1016/j.catena.2014.12.019.

[6] Lombardo U, Grützner C. Active faults and tectonic geomorphology in the Bolivian Amazon. Glob Planet Change 2021;203:103544.
