# Supplementary material for: Constraining the population size estimates of the pre-Columbian Casarabe Culture of Amazonian Bolivia
Source: PLoS One. 2025 May 30;20(5):e0325104. doi: 10.1371/journal.pone.0325104 (PMC12124758; doi:10.1371/journal.pone.0325104)
Supplement: S7 File — File containing extended outputs of MoundSim Population. (PDF) [file pone.0325104.s007.pdf]

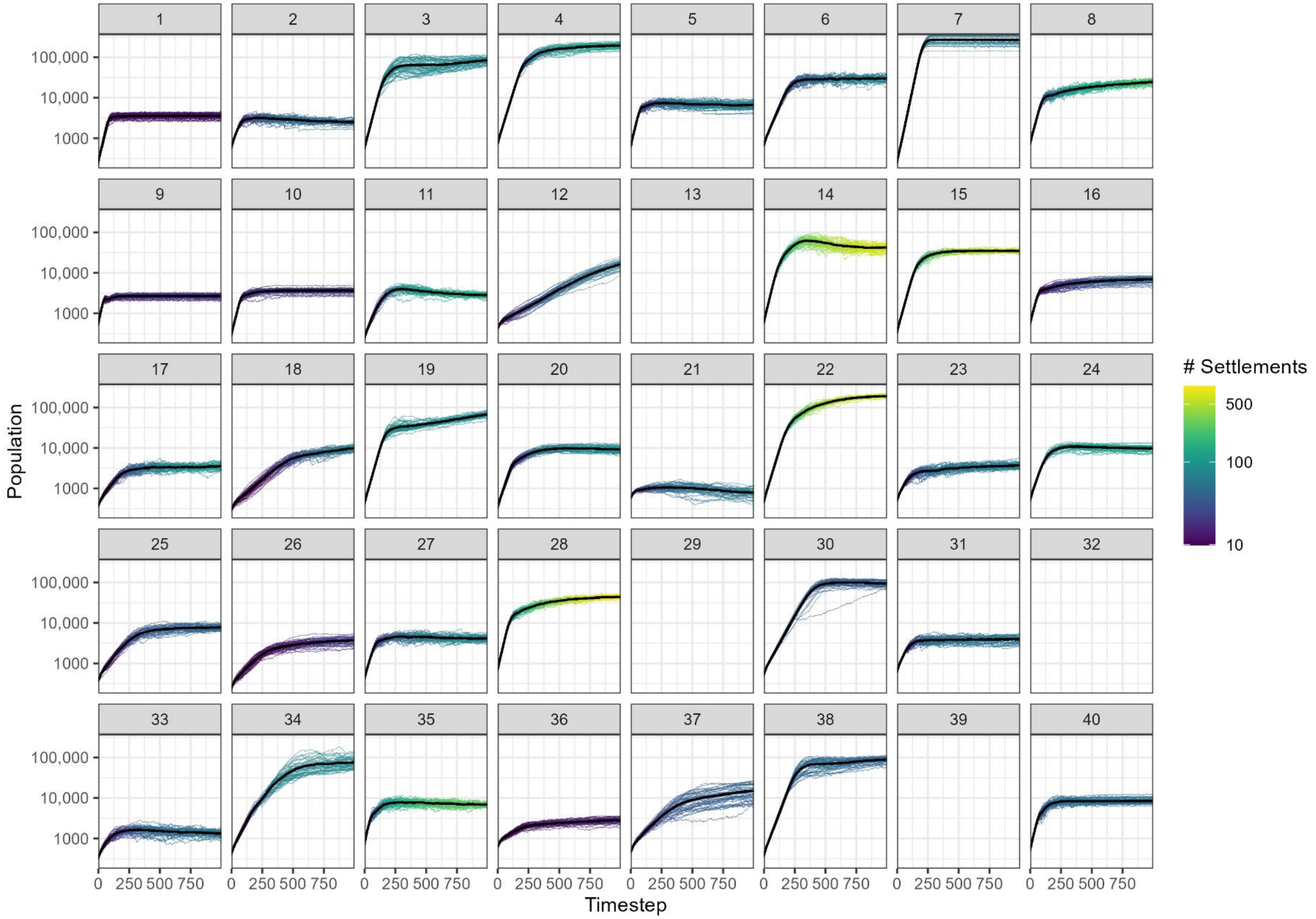

Figure S1: Population estimates for the 36 completed parameter combinations teste. Each line relates to the average population of a single simulation run. Black line reflects the average population estimate. Colour relates to the number of settlements (active + inactive) on the model landscape.

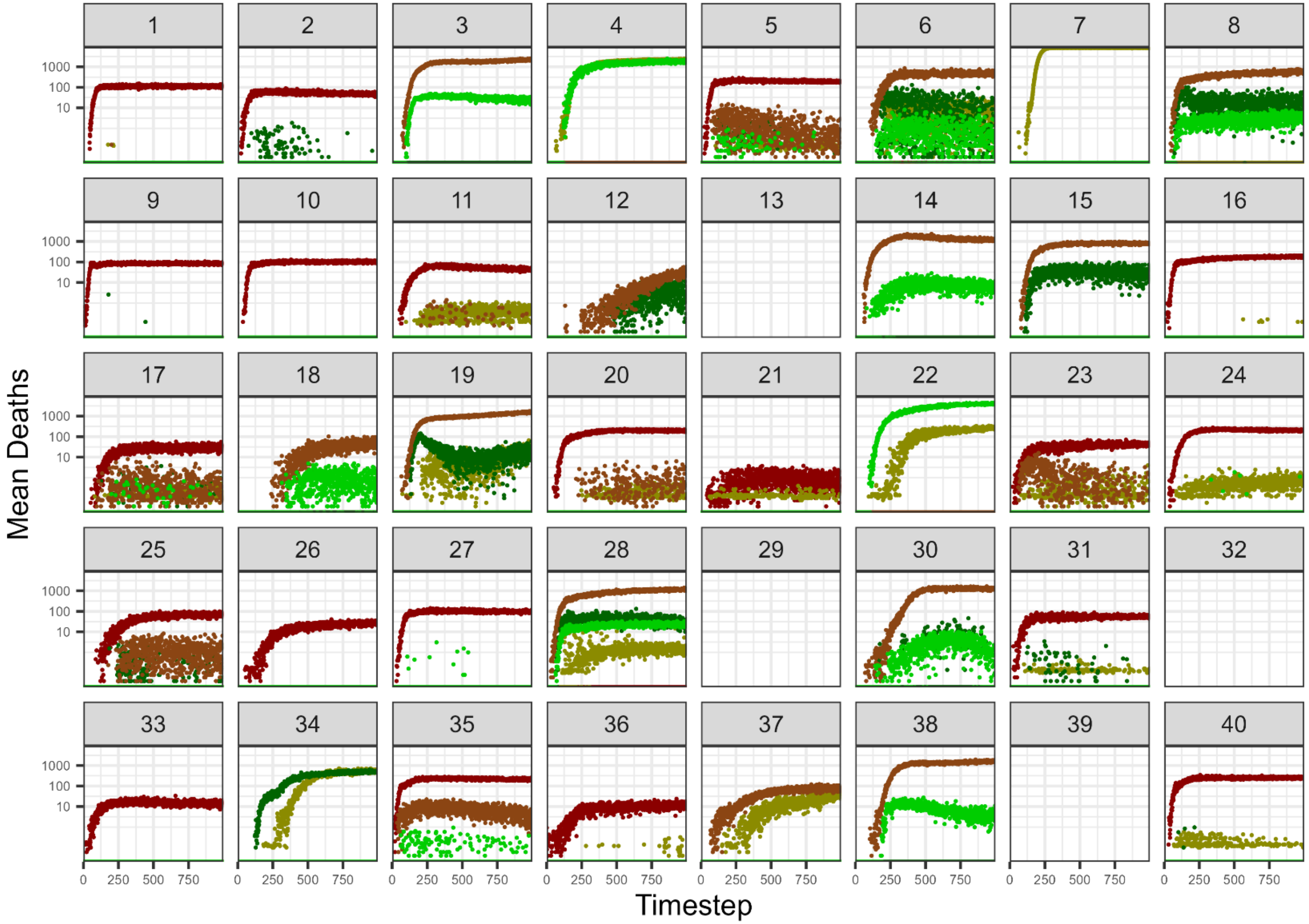

Figure S2: Number of deaths per timestep under each of the 36 completed parameter combinations. Colours denote death type: Maize (Gold); Foraged tree crops (Dark Green); Fuelwood (Brown); Palm Leaves (Light Green); and Protein (Red).

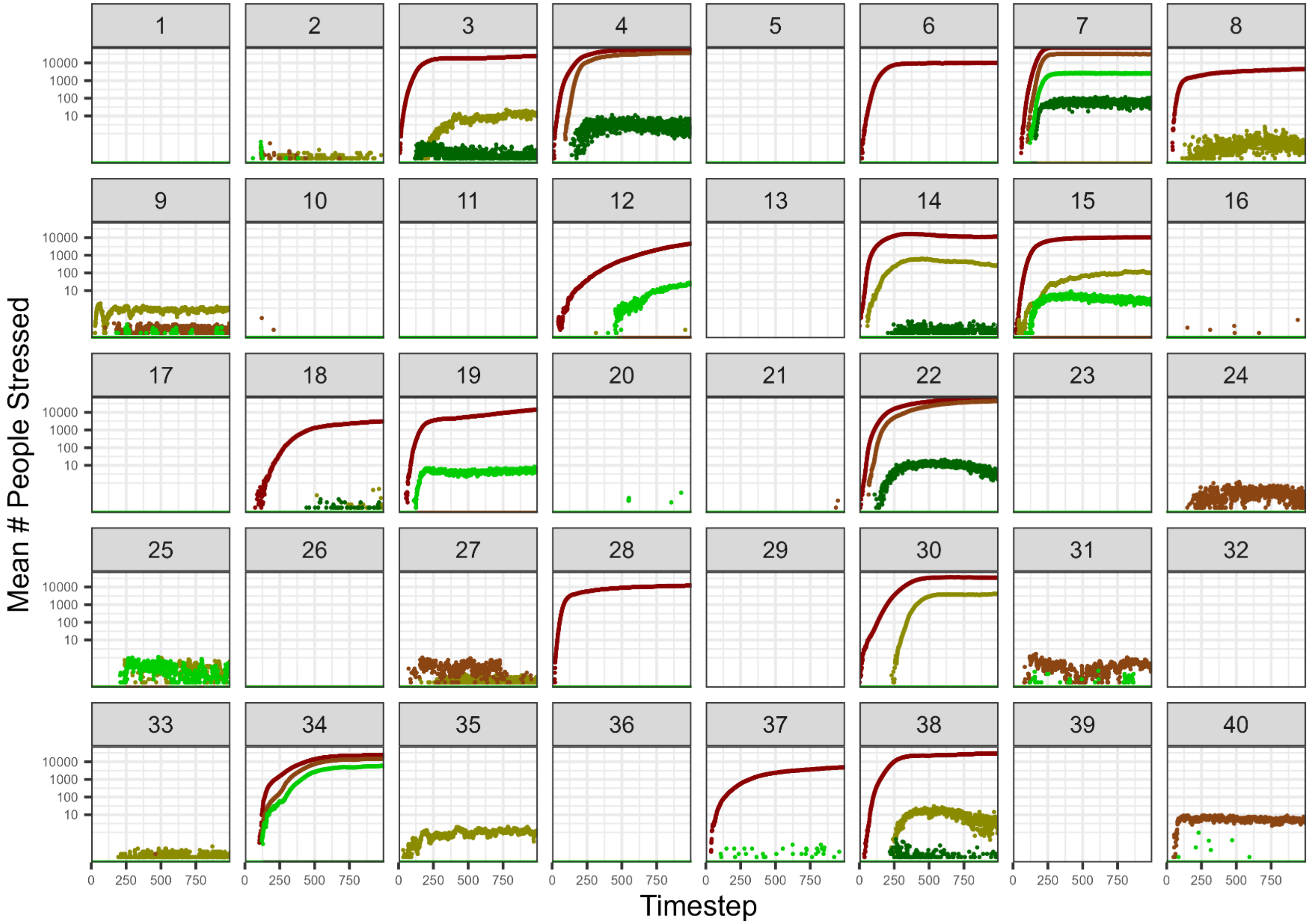

Figure S3: Number of stressed households per timestep under each of the 36 completed parameter combinations. Colours denote death type: Maize (Gold); Foraged tree crops (Dark Green); Fuelwood (Brown); Palm Leaves (Light Green); and Protein (Red).

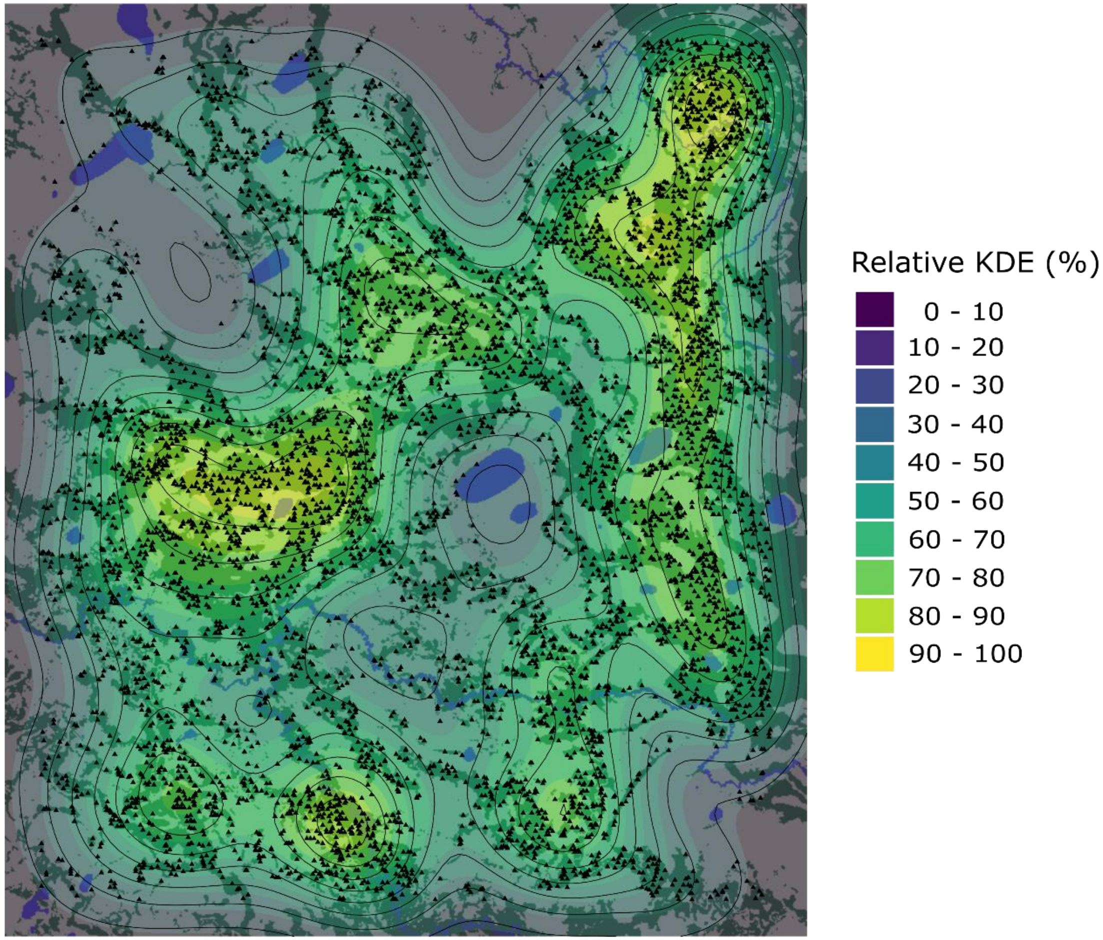

Figure S4: Combined Kernel Density Map for the spatial distribution of population density across the 50 runs performed for Parameter Configuration 4. Black triangles reflect the position of a settlement agent spawned during one of the 50 simulation runs.

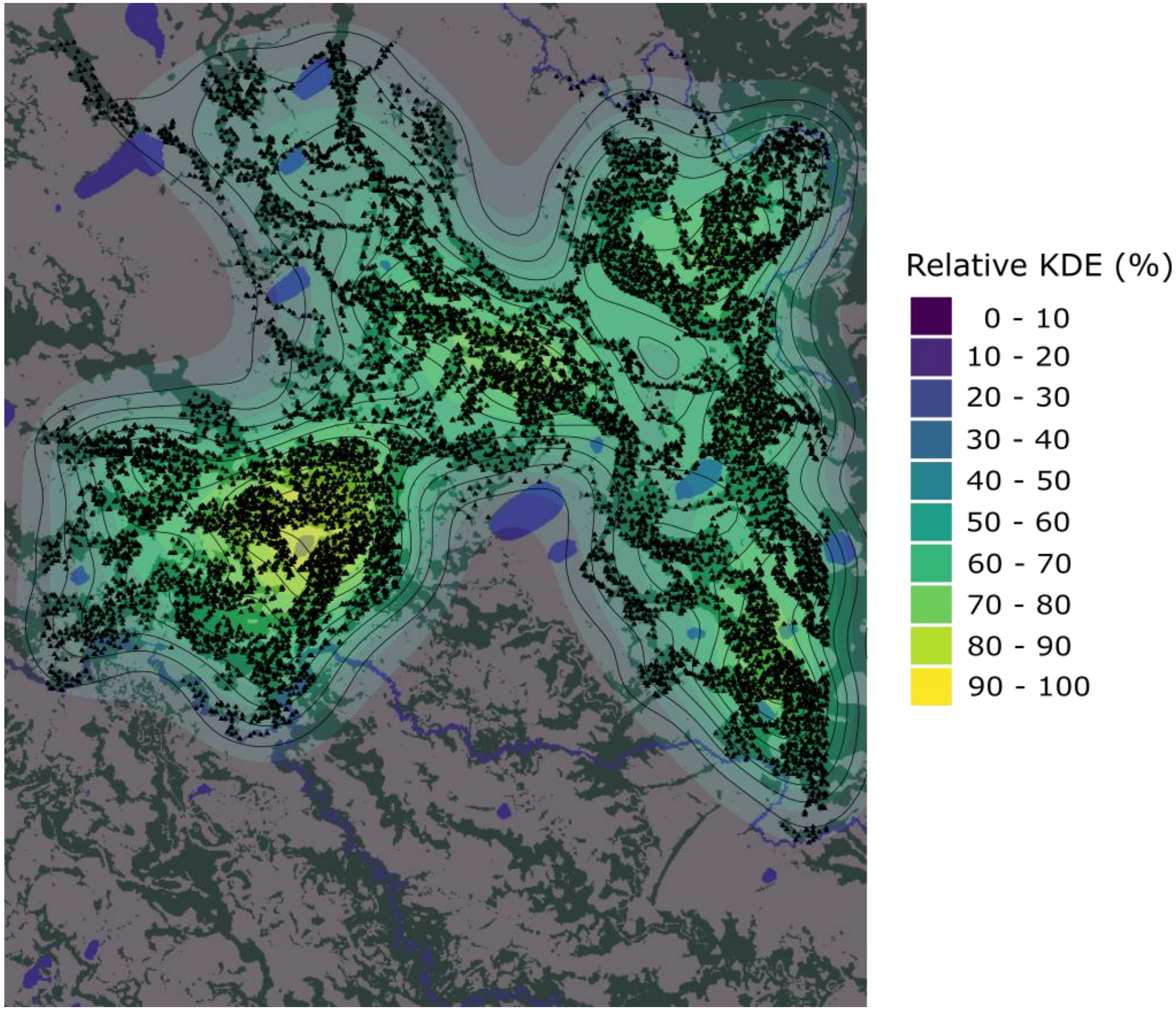

Figure S5: Combined Kernel Density Map for the spatial distribution of population density across the 50 runs performed for Parameter Configuration 8. Black triangles reflect the position of a settlement agent spawned during one of the 50 simulation runs.

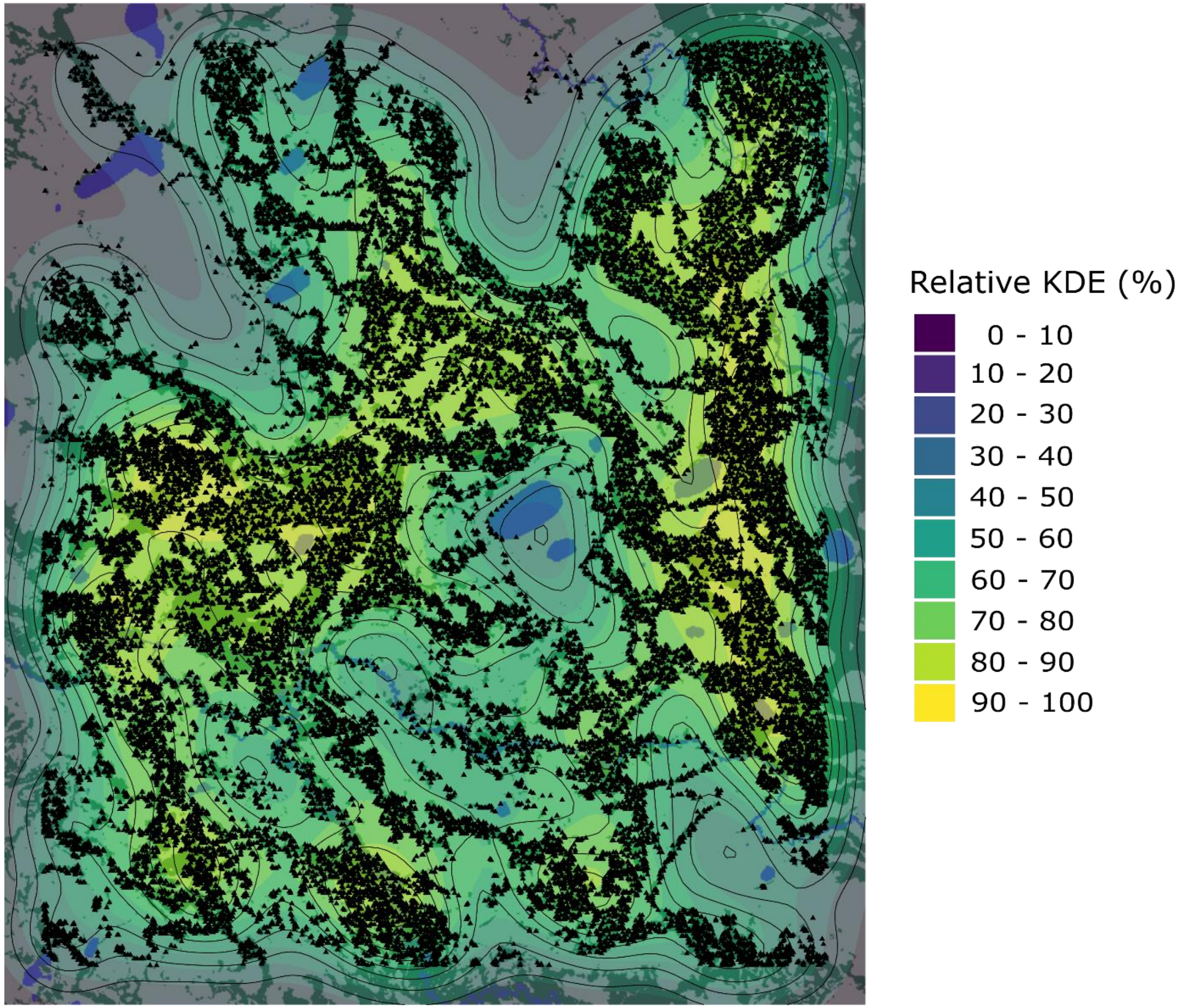

Figure S6: Combined Kernel Density Map for the spatial distribution of population density across the 50 runs performed for Parameter Configuration 14. Black triangles reflect the position of a settlement agent spawned during one of the 50 simulation runs.

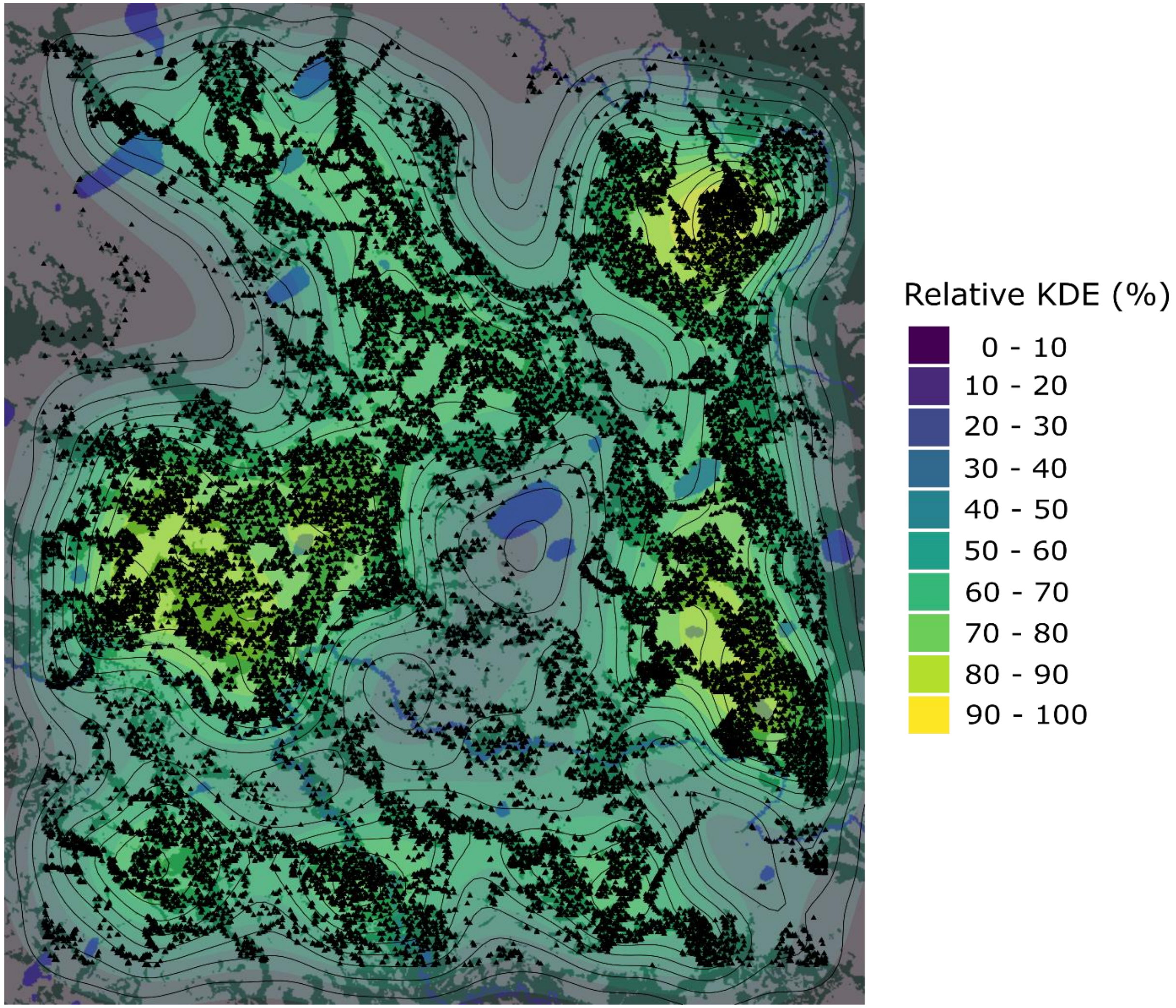

Figure S7: Combined Kernel Density Map for the spatial distribution of population density across the 50 runs performed for Parameter Configuration 15. Black triangles reflect the position of a settlement agent spawned during one of the 50 simulation runs.

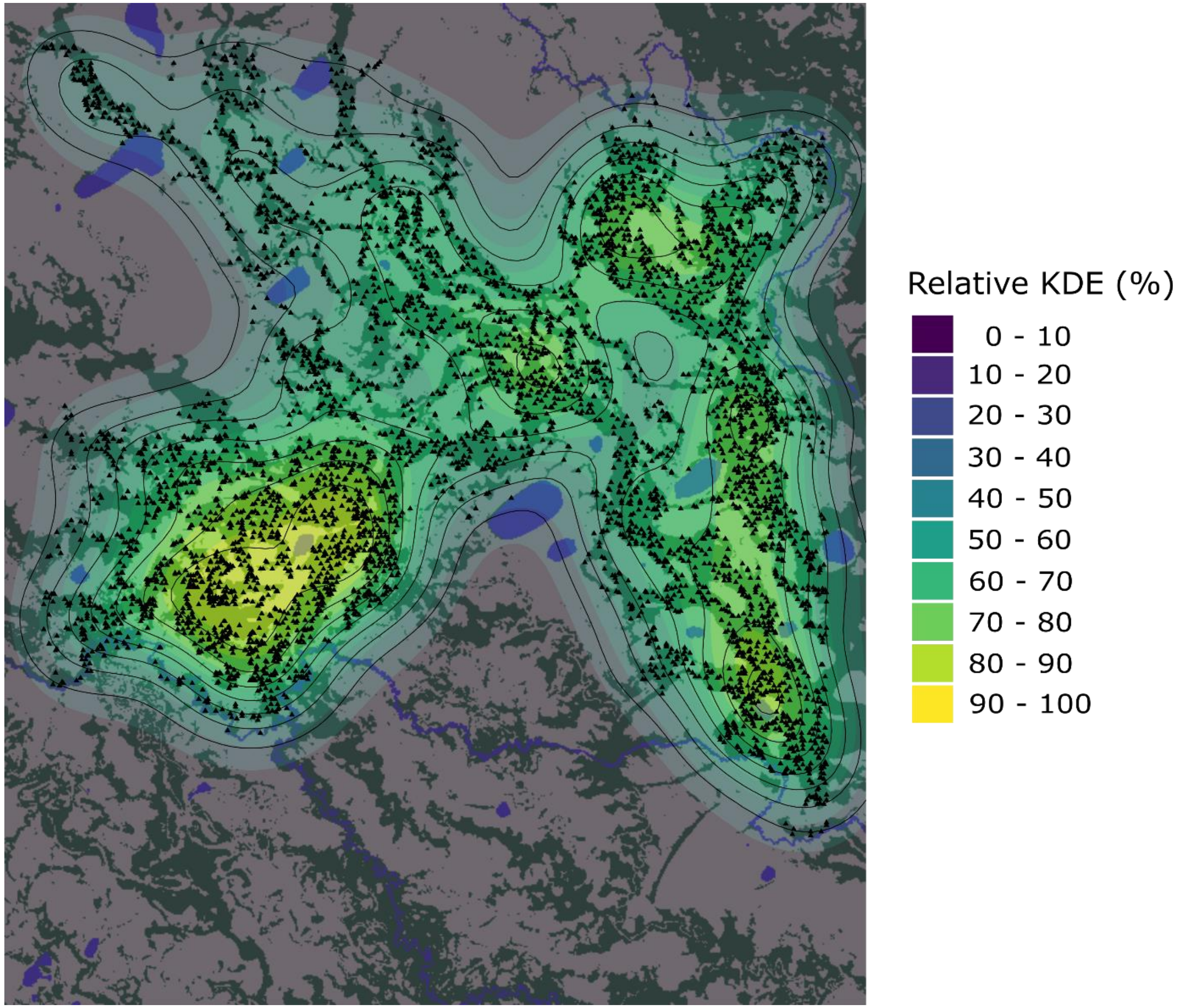

Figure S8: Combined Kernel Density Map for the spatial distribution of population density across the 50 runs performed for Parameter Configuration 17. Black triangles reflect the position of a settlement agent spawned during one of the 50 simulation runs.

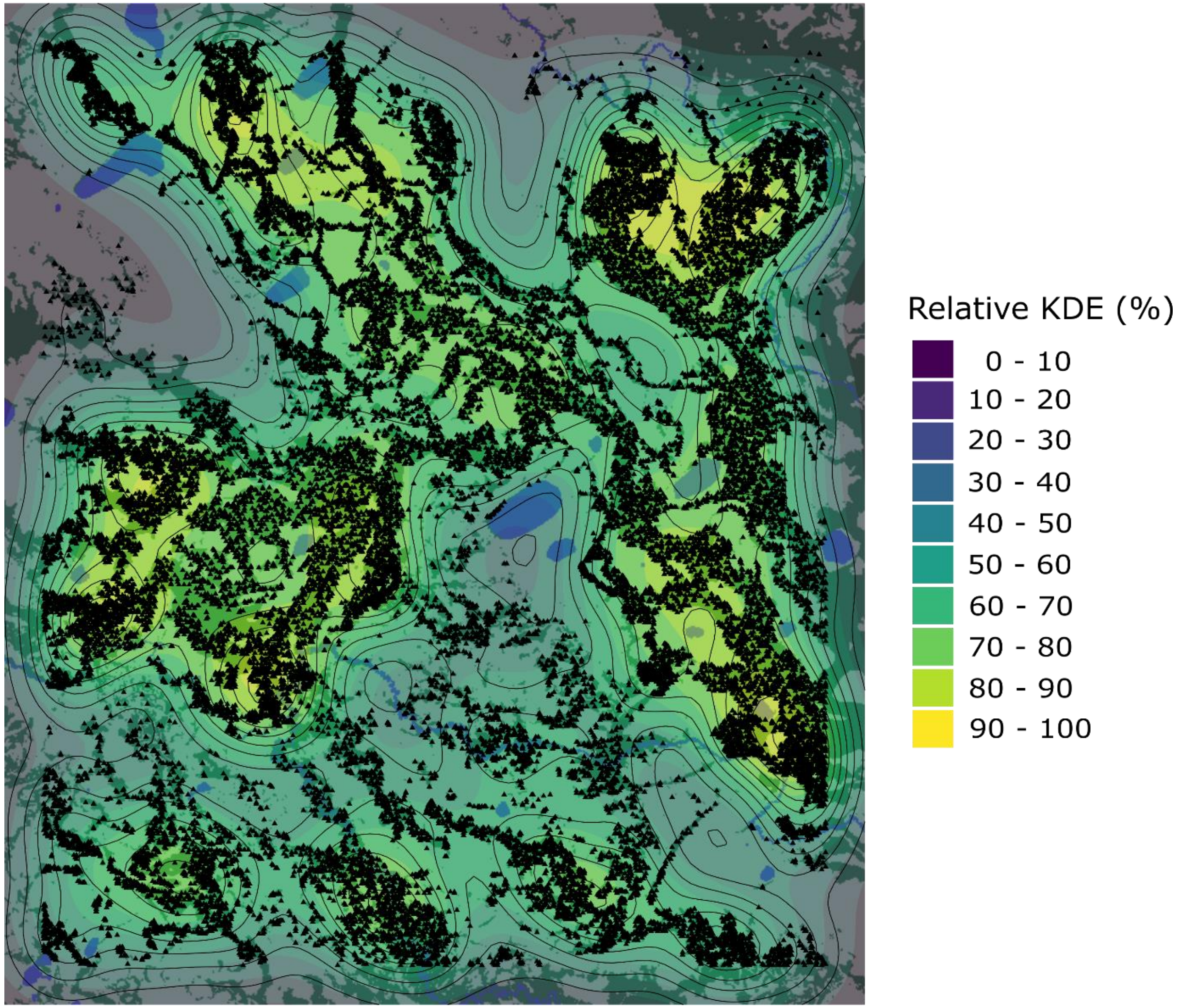

Figure S9: Combined Kernel Density Map for the spatial distribution of population density across the 50 runs performed for Parameter Configuration 22. Black triangles reflect the position of a settlement agent spawned during one of the 50 simulation runs.

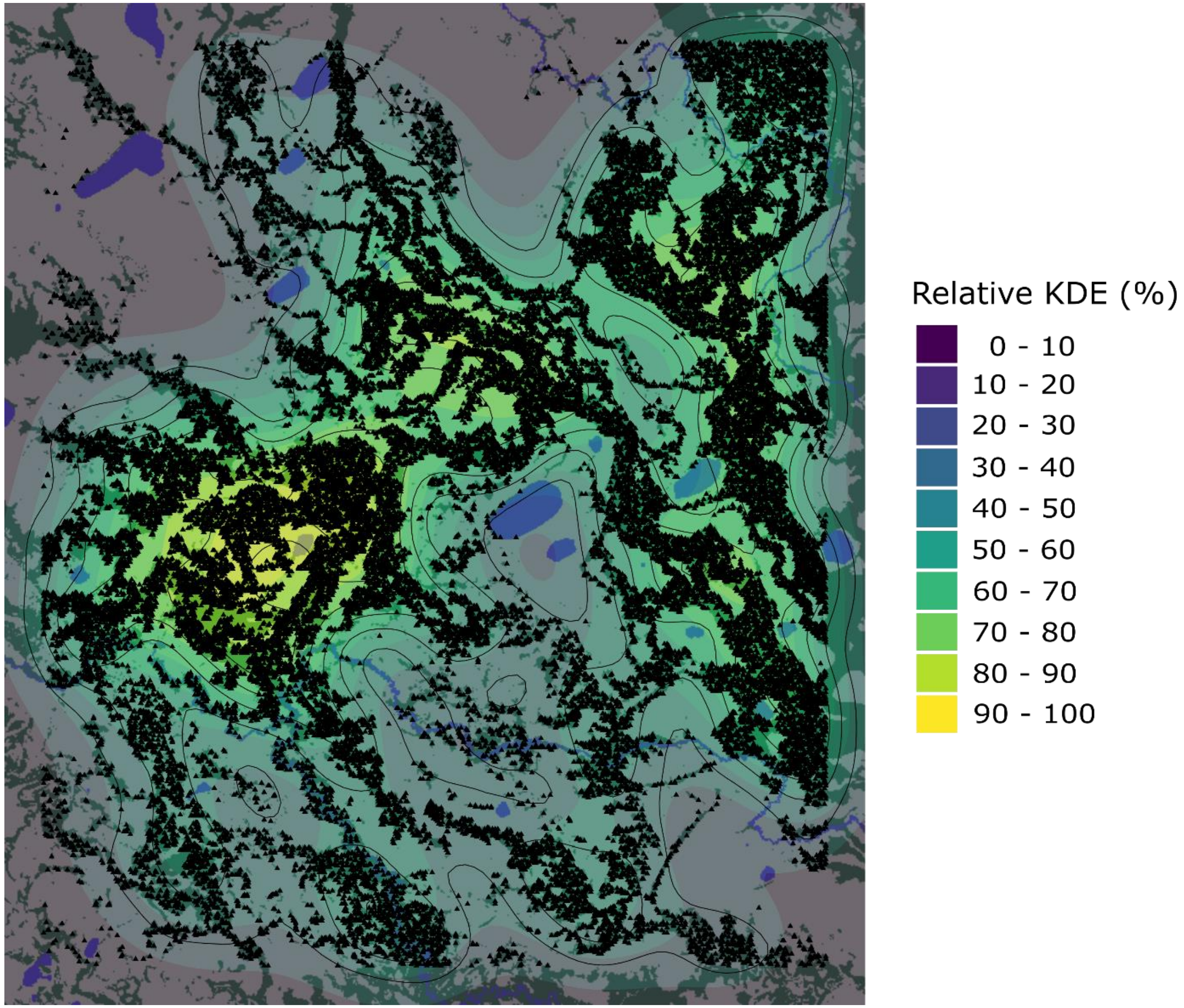

Figure S10: Combined Kernel Density Map for the spatial distribution of population density across the 50 runs performed for Parameter Configuration 28. Black triangles reflect the position of a settlement agent spawned during one of the 50 simulation runs.

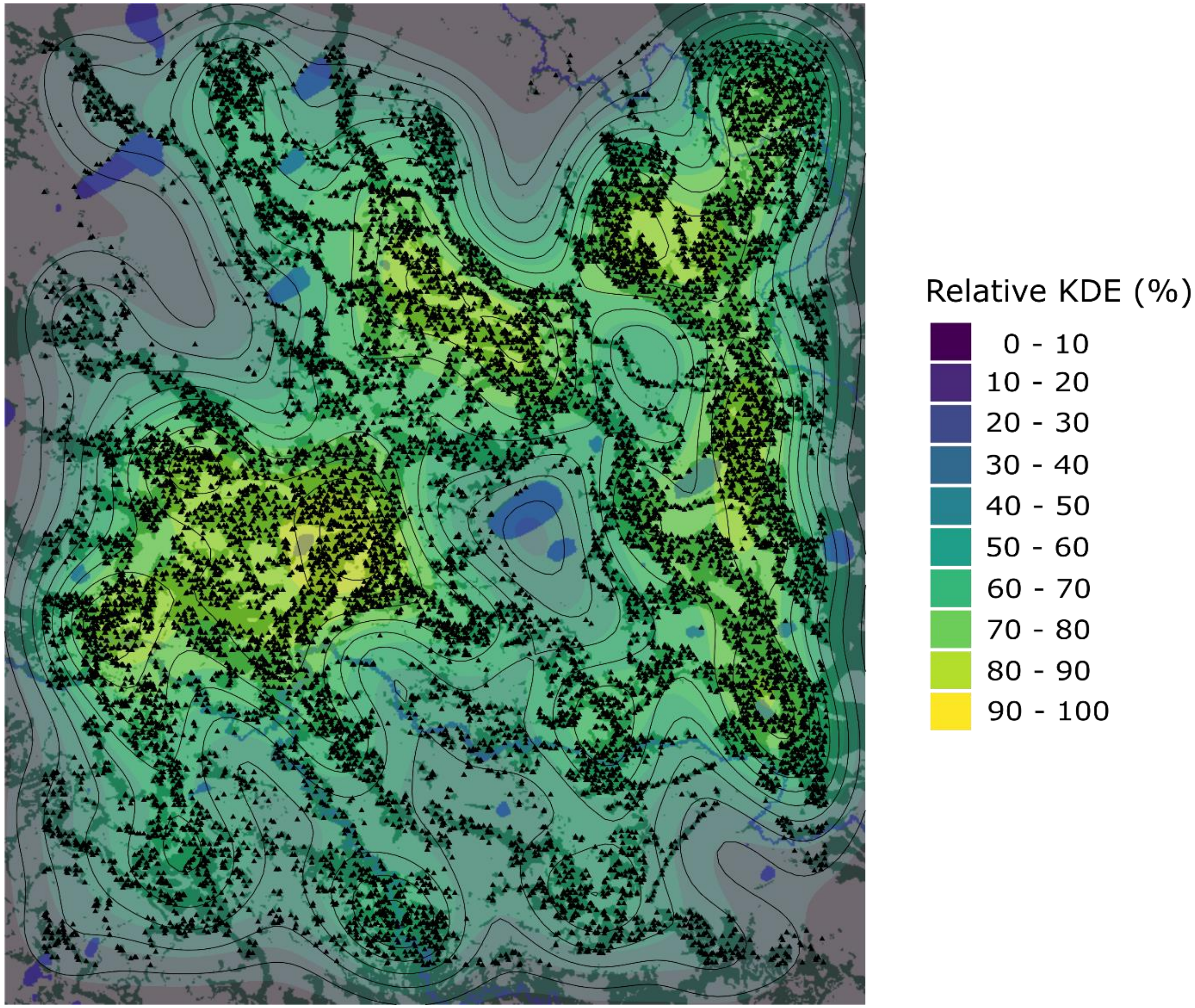

Figure S11: Combined Kernel Density Map for the spatial distribution of population density across the 50 runs performed for Parameter Configuration 35. Black triangles reflect the position of a settlement agent spawned during one of the 50 simulation runs.

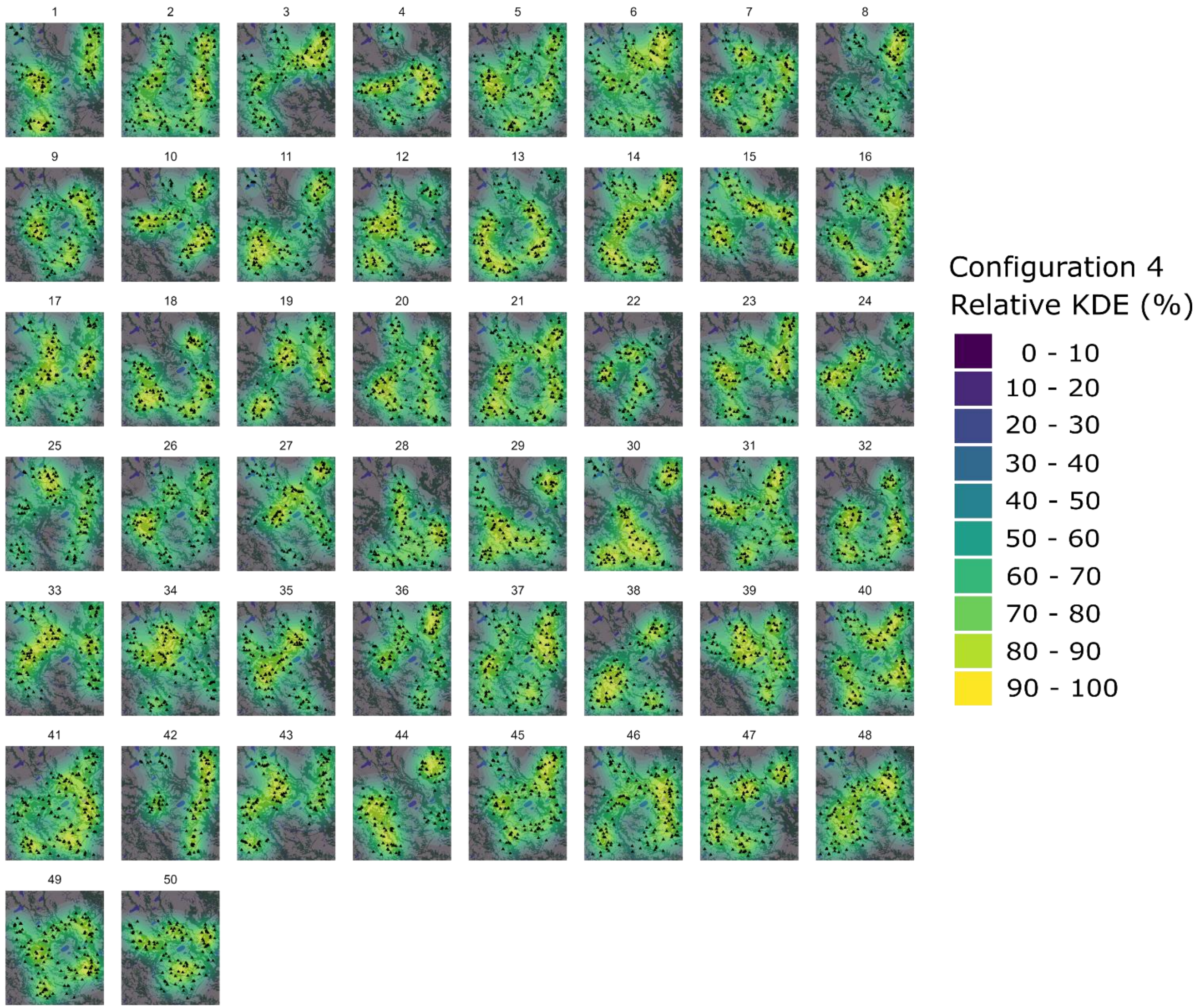

Figure S12: Kernal density maps for each of the 50 simulation runs conducted for Parameter Combination 4. Black triangles reflect the position of a settlement agent spawned during the simulation run.

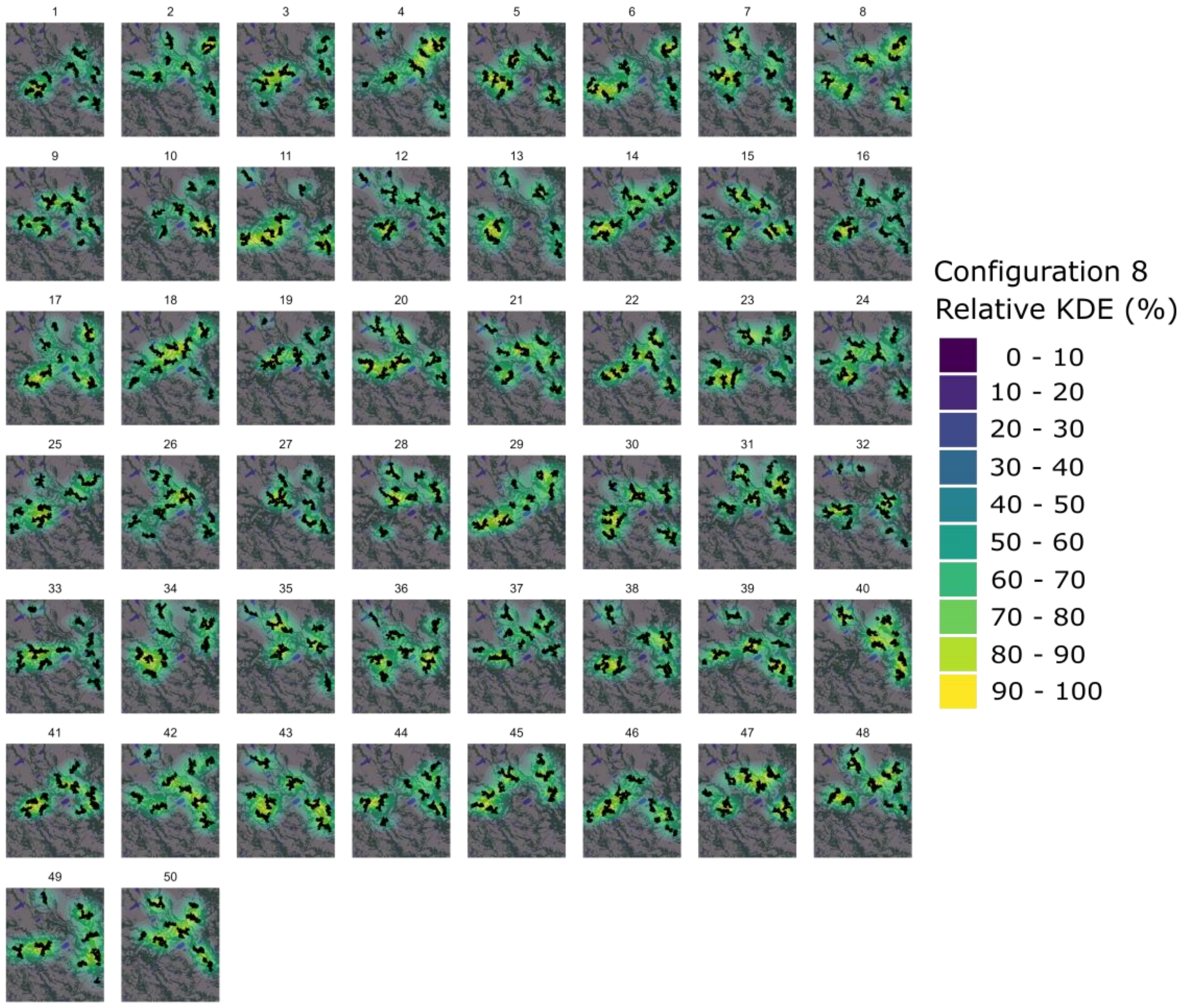

Figure S13: Kernal density maps for each of the 50 simulation runs conducted for Parameter Combination 8. Black triangles reflect the position of a settlement agent spawned during the simulation run.

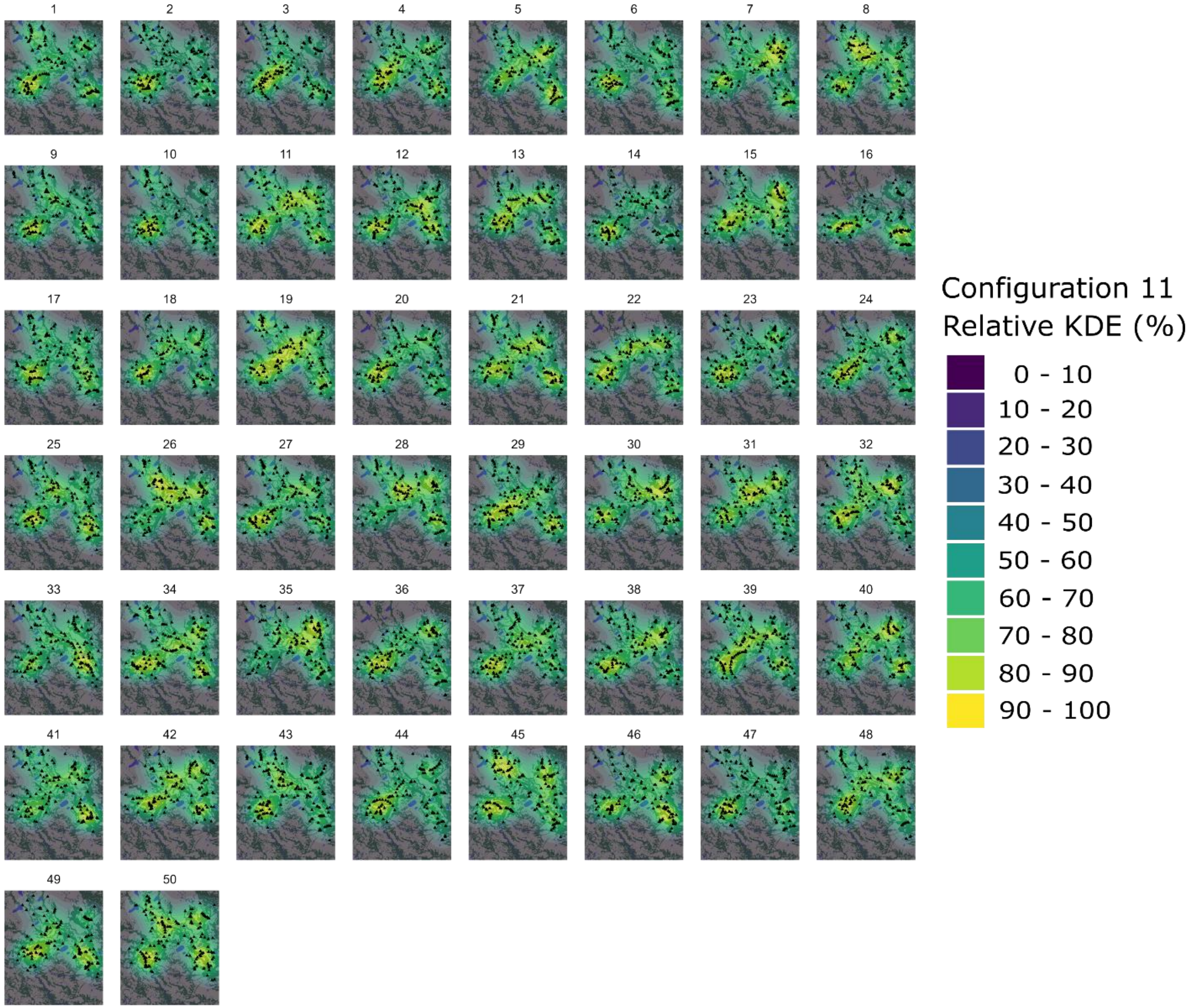

Figure S14: Kernal density maps for each of the 50 simulation runs conducted for Parameter Combination 11. Black triangles reflect the position of a settlement agent spawned during the simulation run.

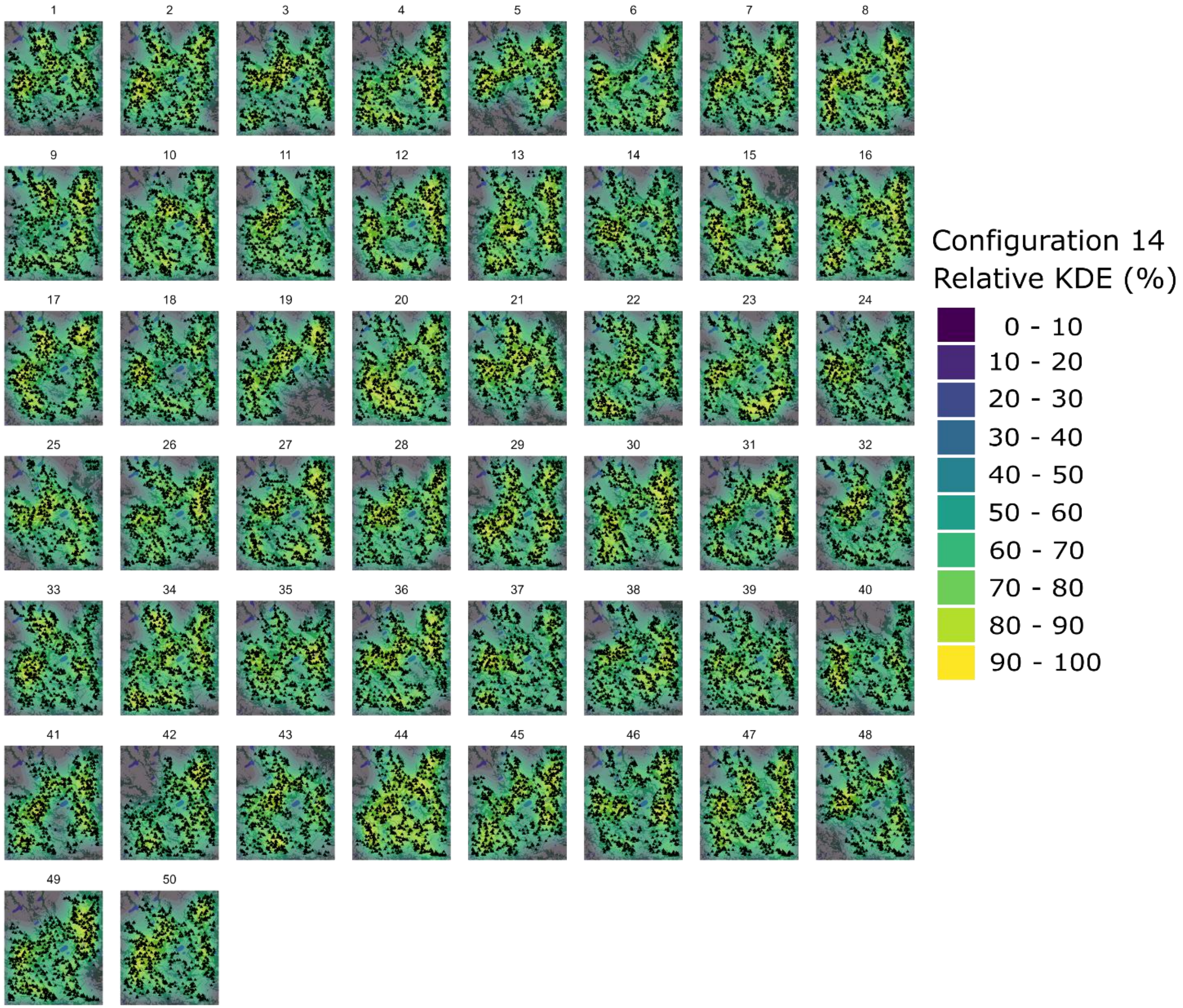

Figure S15: Kernal density maps for each of the 50 simulation runs conducted for Parameter Combination 14. Black triangles reflect the position of a settlement agent spawned during the simulation run.

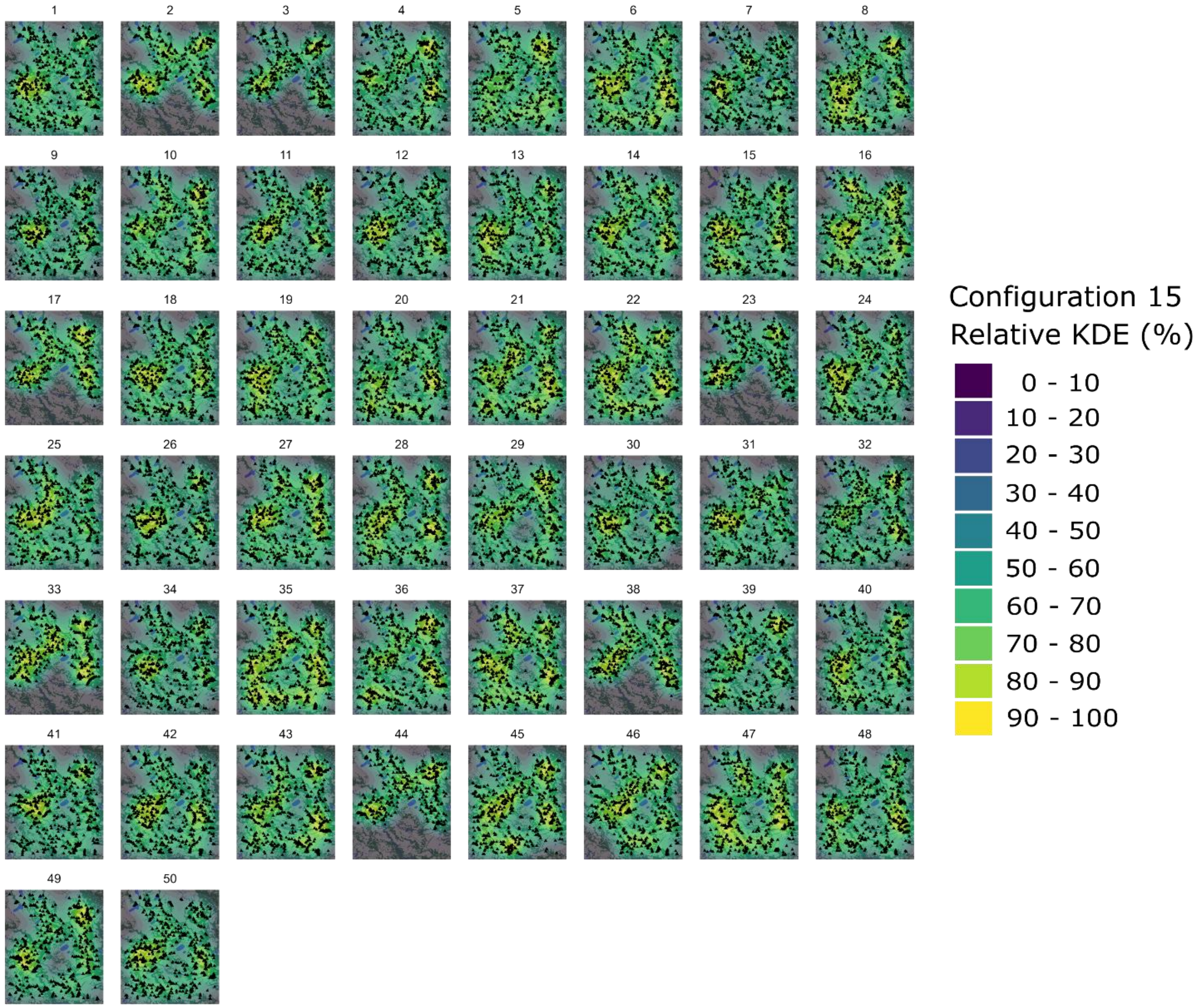

Figure S16: Kernal density maps for each of the 50 simulation runs conducted for Parameter Combination 15. Black triangles reflect the position of a settlement agent spawned during the simulation run.

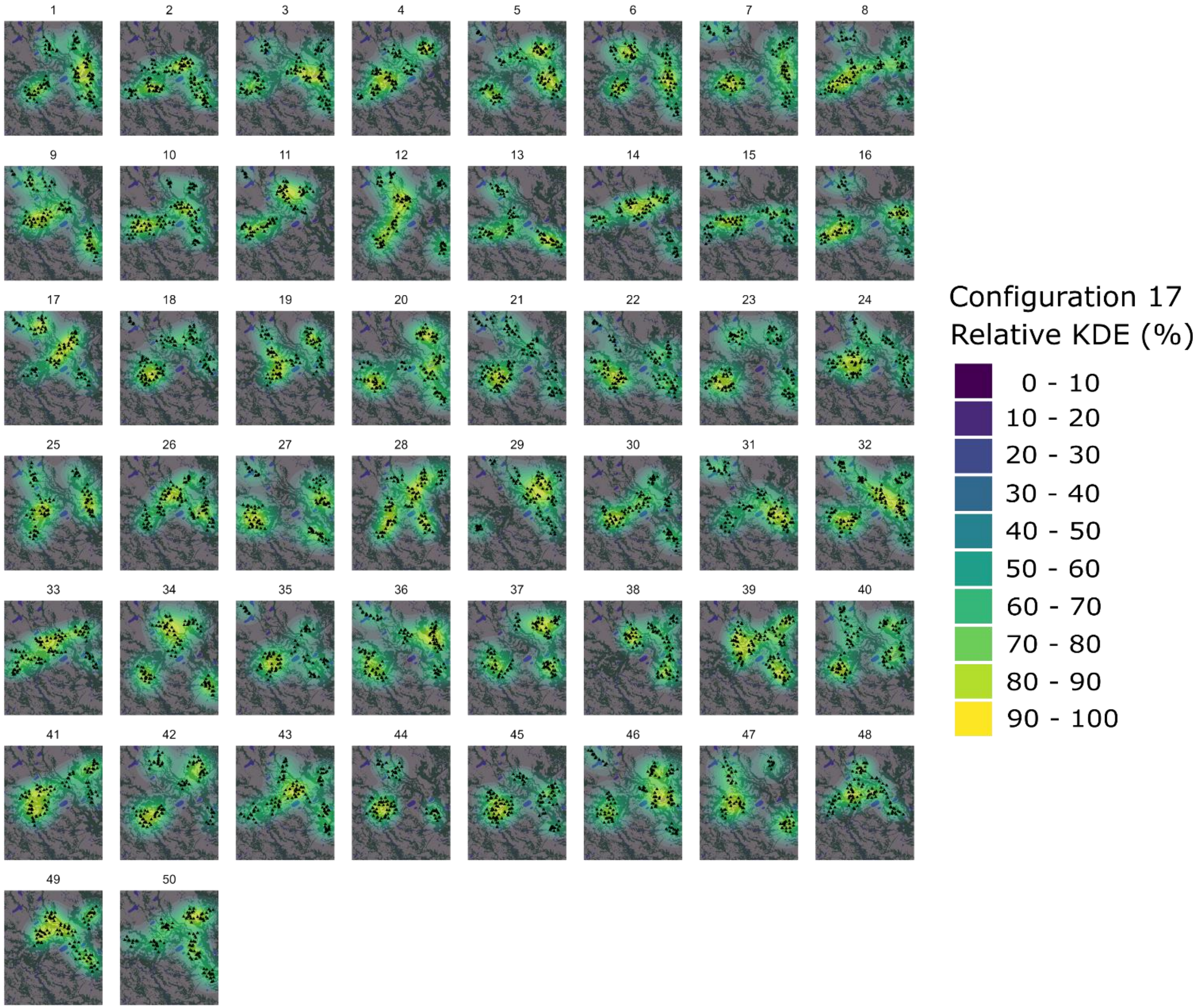

Figure S17: Kernal density maps for each of the 50 simulation runs conducted for Parameter Combination 17. Black triangles reflect the position of a settlement agent spawned during the simulation run.

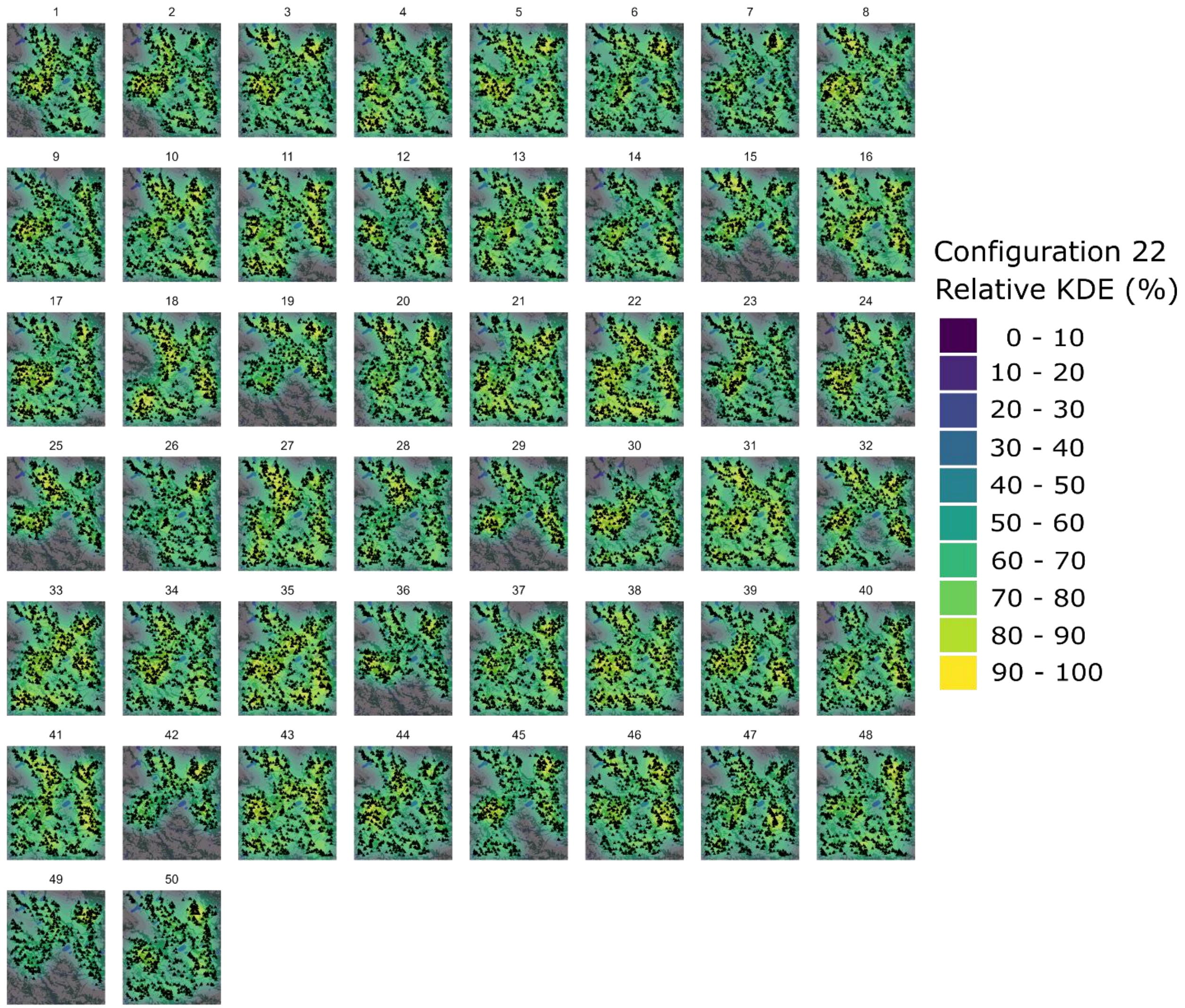

Figure S18: Kernal density maps for each of the 50 simulation runs conducted for Parameter Combination 22. Black triangles reflect the position of a settlement agent spawned during the simulation run.

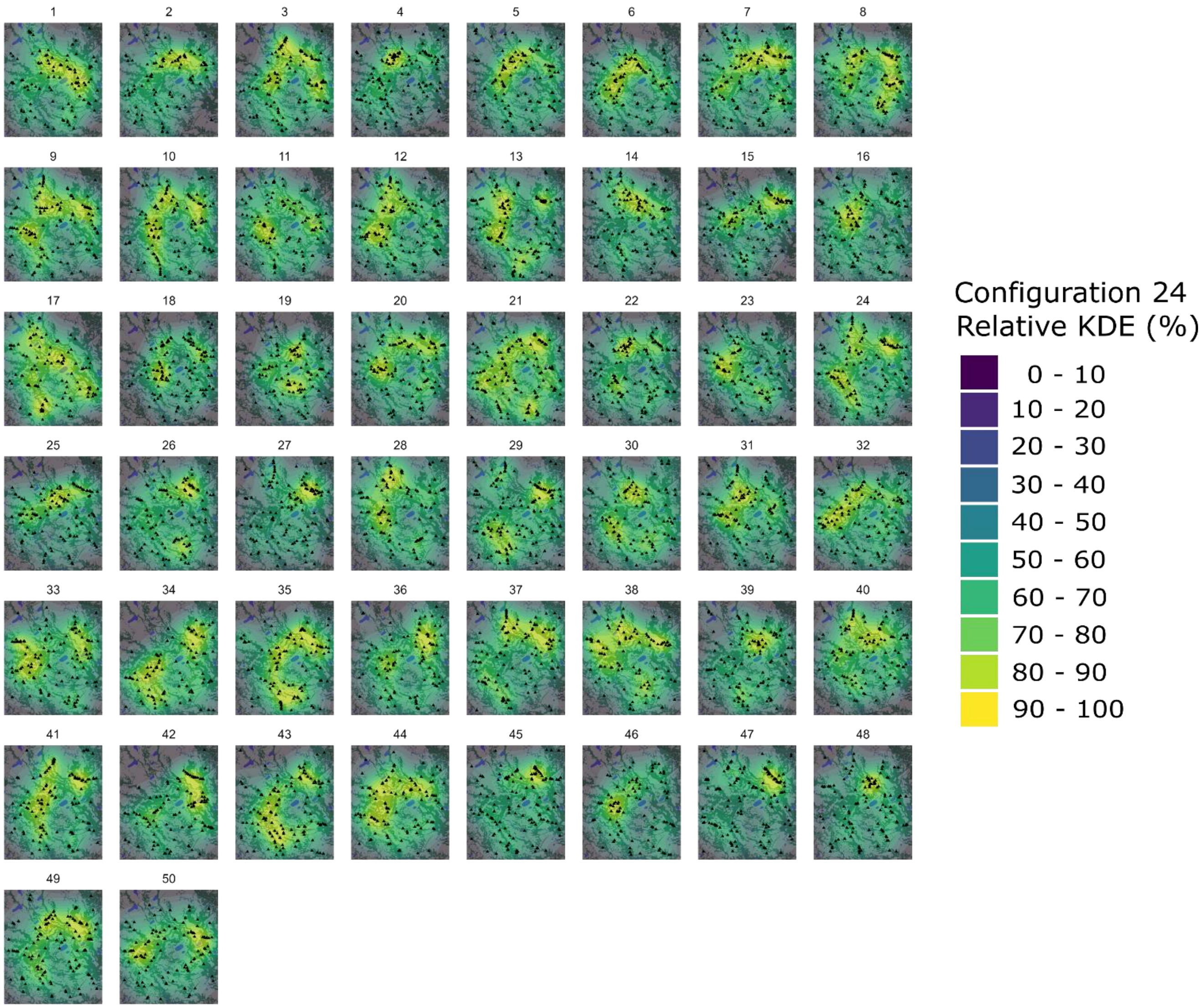

Figure S19: Kernal density maps for each of the 50 simulation runs conducted for Parameter Combination 24. Black triangles reflect the position of a settlement agent spawned during the simulation run.

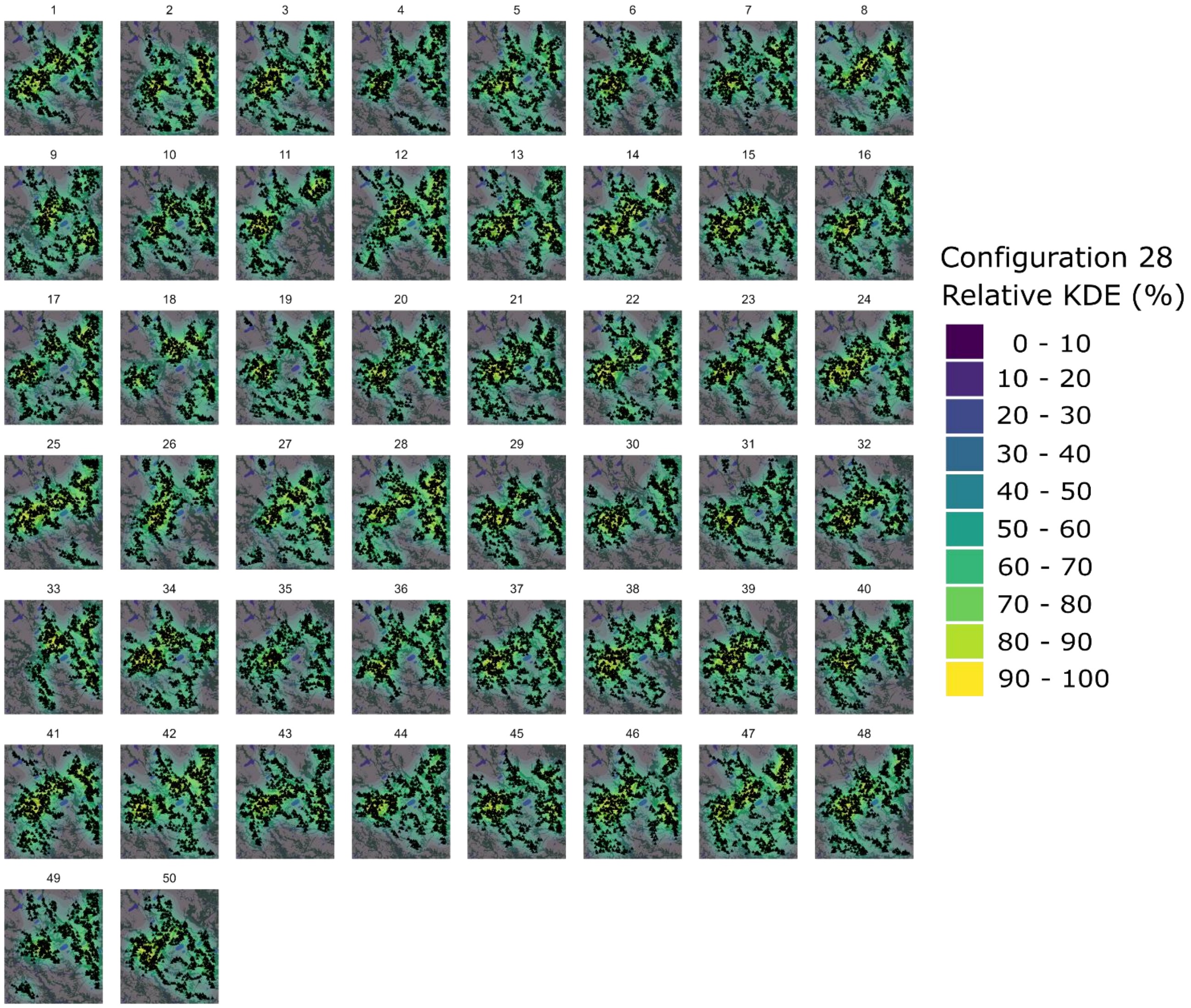

Figure S20: Kernal density maps for each of the 50 simulation runs conducted for Parameter Combination 28. Black triangles reflect the position of a settlement agent spawned during the simulation run.

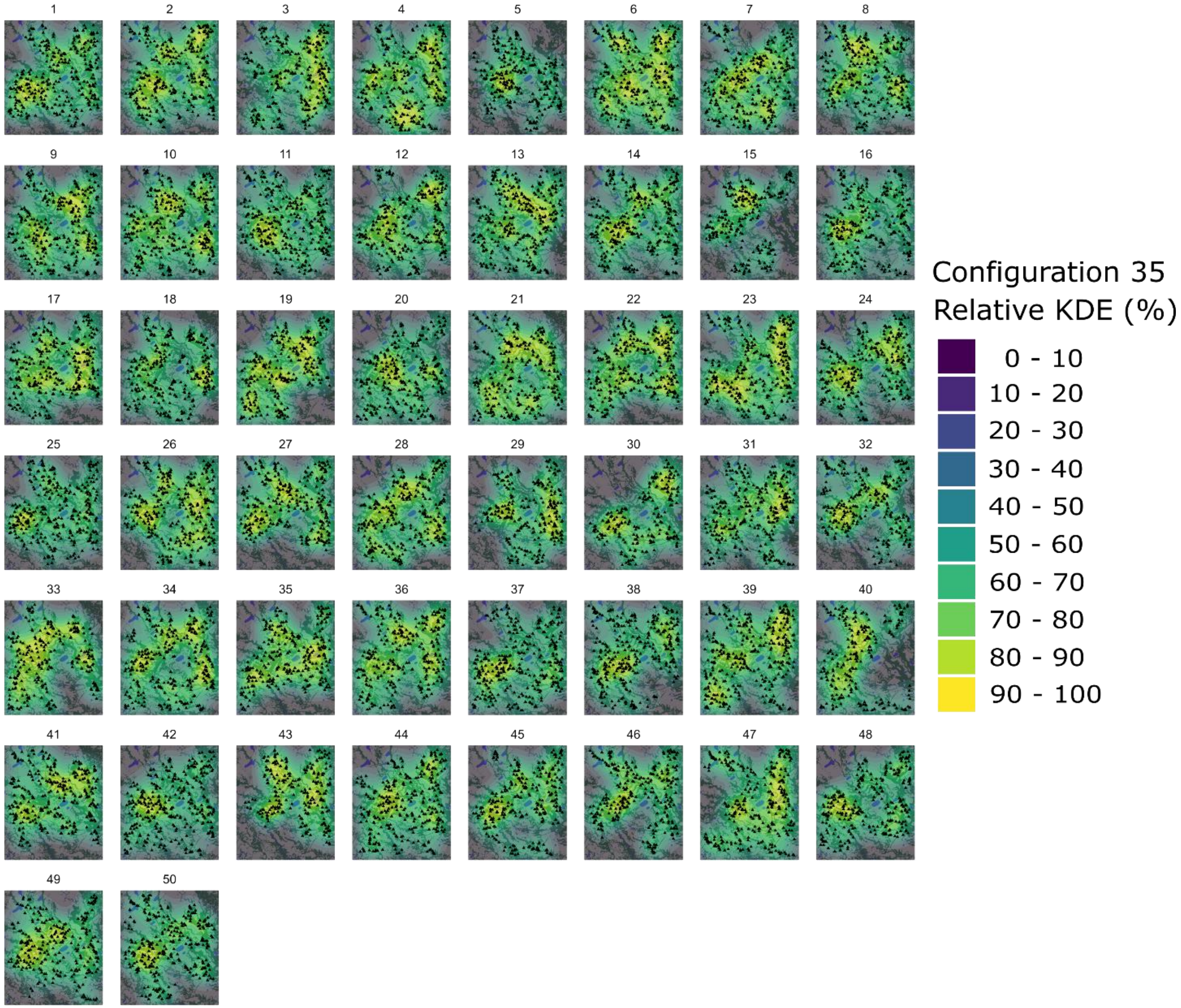

Figure S21: Kernal density maps for each of the 50 simulation runs conducted for Parameter Combination 35. Black triangles reflect the position of a settlement agent spawned during the simulation run.
